# Trends and levels of the global, regional, and national burden of appendicitis between 1990 and 2021: findings from the Global Burden of Disease Study 2021

**DOI:** 10.1016/S2468-1253(24)00157-2

**Published:** 2024-07-17

**Authors:** GBD 2021 Appendicitis Collaborator Group, Hannah Han, Ian D Letourneau, Yohannes Habtegiorgis Abate, Michael Abdelmasseh, Eman Abu-Gharbieh, Tigist Demssew Adane, Bright Opoku Ahinkorah, Aqeel Ahmad, Ali Ahmadi, Ayman Ahmed, Fadwa Naji Alhalaiqa, Salman Khalifah Al-Sabah, Yaser Mohammed Al-Worafi, Hubert Amu, Catalina Liliana Andrei, Amir Anoushiravani, Jalal Arabloo, Aleksandr Y Aravkin, Tahira Ashraf, Sina Azadnajafabad, Nayereh Baghcheghi, Sara Bagherieh, Berihun Bantie Bantie, Mainak Bardhan, Guido Basile, Nebiyou Simegnew Bayleyegn, Amir Hossein Behnoush, Alehegn Bekele, Vijayalakshmi S Bhojaraja, Ali Bijani, Antonio Biondi, Katrin Burkart, Dinh-Toi Chu, Isaac Sunday Chukwu, Natalia Cruz-Martins, Xiaochen Dai, Berecha Hundessa Demessa, Arkadeep Dhali, Daniel Diaz, Thanh Chi Do, Milad Dodangeh, Deepa Dongarwar, Haneil Larson Dsouza, Michael Ekholuenetale, Temitope Cyrus Ekundayo, Iman El Sayed, Muhammed Elhadi, Adeniyi Francis Fagbamigbe, Ildar Ravisovich Fakhradiyev, Pietro Ferrara, Getahun Fetensa, Florian Fischer, Mesfin Gebrehiwot, Melaku Getachew, Mahaveer Golechha, Vivek Kumar Gupta, Joseph R Habib, Najah R Hadi, Nils Haep, Teklehaimanot Gereziher Haile, Erin B Hamilton, Ikramul Hasan, Hamidreza Hasani, Sara Hassanzadeh, Johannes Haubold, Simon I Hay, Khezar Hayat, Olayinka Stephen Ilesanmi, Sumant Inamdar, Chidozie C D Iwu, Assefa N Iyasu, Umesh Jayarajah, Shubha Jayaram, Mohammad Jokar, Nabi Jomehzadeh, Abel Joseph, Nitin Joseph, Charity Ehimwenma Joshua, Ali Kabir, Himal Kandel, Joonas H Kauppila, Phillip M. Kemp Bohan, Himanshu Khajuria, Maseer Khan, Haitham Khatatbeh, Min Seo Kim, Adnan Kisa, Farzad Kompani, Hamid Reza Koohestani, Rakesh Kumar, Thao Thi Thu Le, Munjae Lee, Seung Won Lee, Ming-Chieh Li, Stephen S Lim, Chun-Han Lo, Raimundas Lunevicius, Kashish Malhotra, Andrea Maugeri, Rishi P Mediratta, Tuomo J Meretoja, Tomislav Mestrovic, Mohammad Mirza-Aghazadeh-Attari, Nouh Saad Mohamed, Ali H Mokdad, Lorenzo Monasta, Mohammad Ali Moni, Maryam Moradi, Vincent Mougin, George Duke Mukoro, Efren Murillo-Zamora, Christopher J L Murray, Mukhammad David Naimzada, Hastyar Hama Rashid Najmuldeen, Zuhair S Natto, Ionut Negoi, Hien Quang Nguyen, Taxiarchis Konstantinos Nikolouzakis, Isaac Iyinoluwa Olufadewa, Jagadish Rao Padubidri, Ashok Pandey, Romil R Parikh, Hoang Tran Pham, Richard Charles G Pollok, Mehran Rahimi, Vafa Rahimi-Movaghar, Mosiur Rahman, Shayan Rahmani, Mohammad-Mahdi Rashidi, Salman Rawaf, Jennifer Rickard, Hamidreza Rouientan, Simanta Roy, Basema Ahmad Saddik, Umar Saeed, Mohamed A Saleh, Sana Salehi, Abdallah M Samy, Juan Sanabria, Senthilkumar Sankararaman, Austin E Schumacher, Subramanian Senthilkumaran, Pritik A Shah, Sina Shool, Migbar Mekonnen Sibhat, Negussie Boti Sidamo, Jasvinder A Singh, Bogdan Socea, Yonatan Solomon, Saraswathy Sreeram, Seyyed Mohammad Tabatabaei, Ker-Kan Tan, Seyed Mohammad Tavangar, Yibekal Manaye Tefera, Nikhil Kenny Thomas, Jansje Henny Vera Ticoalu, Guesh Mebrahtom Tsegay, Dejen Tsegaye, Sana Ullah, Abachebissa Nuru Usman, Rohollah Valizadeh, Massimiliano Veroux, Georgios-Ioannis Verras, Theo Vos, Mei Wang, Song Wang, Dakshitha Praneeth Wickramasinghe, Galal Yahya, Iman Zare, Armin Zarrintan, Zhi-Jiang Zhang, M Ashworth Dirac

## Abstract

**Background:**

Appendicitis is a common surgical emergency that poses a large clinical and economic burden. Understanding the global burden of appendicitis is crucial for evaluating unmet needs and implementing and scaling up intervention services to reduce adverse health outcomes. This study aims to provide a comprehensive assessment of the global, regional, and national burden of appendicitis, by age and sex, from 1990 to 2021.

**Methods:**

Vital registration and verbal autopsy data, the Cause of Death Ensemble model (CODEm), and demographic estimates from the Global Burden of Diseases, Injuries, and Risk Factors Study (GBD) were used to estimate cause-specific mortality rates (CSMRs) for appendicitis. Incidence data were extracted from insurance claims and inpatient discharge sources and analysed with disease modelling meta-regression, version 2.1 (DisMod-MR 2.1). Years of life lost (YLLs) were estimated by combining death counts with standard life expectancy at the age of death. Years lived with disability (YLDs) were estimated by multiplying incidence estimates by an average disease duration of 2 weeks and a disability weight for abdominal pain. YLLs and YLDs were summed to estimate disability-adjusted life-years (DALYs).

**Findings:**

In 2021, the global age-standardised mortality rate of appendicitis was 0·358 (95% uncertainty interval [UI] 0·311–0·414) per 100 000. Mortality rates ranged from 1·01 (0·895–1·13) per 100 000 in central Latin America to 0·054 (0·0464–0·0617) per 100 000 in high-income Asia Pacific. The global age-standardised incidence rate of appendicitis in 2021 was 214 (174–274) per 100 000, corresponding to 17 million (13·8–21·6) new cases. The incidence rate was the highest in high-income Asia Pacific, at 364 (286–475) per 100 000 and the lowest in western sub-Saharan Africa, at 81·4 (63·9–109) per 100 000. The global age-standardised rates of mortality, incidence, YLLs, YLDs, and DALYs due to appendicitis decreased steadily between 1990 and 2021, with the largest reduction in mortality and YLL rates. The global annualised rate of decline in the DALY rate was greatest in children younger than the age of 10 years. Although mortality rates due to appendicitis decreased in all regions, there were large regional variations in the temporal trend in incidence. Although the global age-standardised incidence rate of appendicitis has steadily decreased between 1990 and 2021, almost half of GBD regions saw an increase of greater than 10% in their age-standardised incidence rates.

**Interpretation:**

Slow but promising progress has been observed in reducing the overall burden of appendicitis in all regions. However, there are important geographical variations in appendicitis incidence and mortality, and the relationship between these measures suggests that many people still do not have access to quality health care. As the incidence of appendicitis is rising in many parts of the world, countries should prepare their health-care infrastructure for timely, high-quality diagnosis and treatment. Given the risk that improved diagnosis may counterintuitively drive apparent rising trends in incidence, these efforts should be coupled with improved data collection, which will also be crucial for understanding trends and developing targeted interventions.

**Funding:**

Bill and Melinda Gates Foundation.

## Introduction

Appendicitis is a widespread surgical emergency, often occurring in the second and third decades of life.^1^, ^2^ Timely treatment is crucial to prevent severe and potentially life-threatening complications. Appendicitis has long been treated with open appendicectomy, usually in a hospital setting. In recent decades, a growing number of countries have begun adopting laparoscopic appendicectomy.^3^ Laparoscopic appendicectomy can be done safely and effectively in an outpatient or day-surgery setting and is associated with a shorter recovery time and a lower risk of postoperative complications.^4^, ^5^, ^6^ Non-operative management of appendicitis with antibiotic therapy alone has also received attention,^7^, ^8^ although appendicectomy remains the dominant form of treatment for appendicitis worldwide.^3^

To reduce the morbidity and mortality associated with appendicitis, early and accurate diagnosis and timely treatment delivery are key.^9^ A large proportion of the world's population, however, does not have access to any quality health care, let alone surgical care,^10^, ^11^, ^12^ posing a considerable clinical and economic burden that could otherwise be prevented.^13^, ^14^, ^15^, ^16^ In countries with scarce resources, deciding which health concerns to prioritise for maximum impact is of paramount importance. Reducing mortality from infections that pose a substantial threat to public health (such as tuberculosis, HIV, and malaria) has been a longstanding priority. This emphasis has started to shift in recent years to target non-communicable diseases (NCDs) such as cancer and cardiovascular diseases. Some argue that global efforts to address the rising burden of NCDs have overlooked certain surgically treated conditions, including appendicitis and especially in low-income and middle-income countries, despite these diseases probably constituting a substantial proportion of the total disease burden.^17^, ^18^, ^19^

**Research in context**

**Evidence before this study**

Several global estimates of appendicitis have been published previously based on findings from the Global Burden of Diseases, Injuries, and Risk Factors Study (GBD). The last publication was in 2023 by Guan and colleagues, which was based on results from GBD 2019. This study showed a slight increase in the global age-standardised incidence rate of appendicitis between 1990 and 2019. Outside GBD, the only comparable systematic review published on the global burden of appendicitis is by Ferris and colleagues in 2017, which found that the pooled incidence of appendicitis began stabilising in high-income countries in the late 20th century, while it increased in newly industrialised countries in Asia, the Middle East, South America, and Africa. In addition to these studies, epidemiological studies have been published to examine the incidence of appendicitis or appendicectomy in different communities. Epidemiological data are limited, however, in many parts of the world, particularly in areas where resources are scarce.

**Added value of this study**

The present study, which is based on results from GBD 2021, provides the most up-to-date global, regional, and national estimates of morbidity and mortality due to appendicitis, by age and sex, for 204 countries and territories between 1990 and 2021. Our study builds on previous work, with estimates for additional locations and years, and a comprehensive assessment of appendicitis incidence incorporating information from both inpatient and outpatient care. This is the first formal report prepared by the GBD Appendicitis Collaborators, providing a detailed description of input data sources, data processing steps, and a nuanced discussion of the strengths and limitations of the data sources and methodology used.

**Implications of all the available evidence**

This study demonstrates a decreasing global trend in age-standardised disability-adjusted life-year (DALY) rates of appendicitis between 1990 and 2021. The downward trend was more rapid in the rates of mortality and years of life lost (YLLs) compared to incidence and years lived with disability (YLDs). This observation suggests improved survival outcomes in patients with appendicitis on a global scale. Despite the promising trend in mortality rates across all 21 GBD regions, we observed notable variations in the temporal trends of incidence and YLDs. In particular, the incidence of appendicitis is on the rise in many lower-income countries where access to quality health care is scarce. This study highlights the need for investment into context-specific, targeted strategies to address the disparate trends in the burden of appendicitis in various regions.

Understanding the levels and trends of appendicitis and other conditions that do not receive a considerable amount of global health focus is a key step to guide effective policy and planning. Burden estimates enable stakeholders and policy makers to identify data gaps, evaluate unmet needs, and implement and scale up intervention services appropriately. Previous studies have noted geographical variation in the incidence of appendicitis, with higher rates in high-income countries and lower rates in low-income and middle-income countries (LMICs).^20^, ^21^, ^22^, ^23^, ^24^, ^25^ A systematic review published by Ferris and colleagues^20^ highlights an upward trend in appendicitis incidence in LMICs, but stabilising incidence rates in many high-income countries. Epidemiological data on appendicitis, however, remain scarce in many parts of the world, particularly in lower-income countries where access to quality health care is scare. This limitation makes it difficult to understand the burden of appendicitis in low-resource settings and create effective diagnosis and management strategies to curb the disease burden.

In an effort to provide a comprehensive assessment of the global burden of appendicitis, we estimated the fatal and non-fatal burden of appendicitis as part of the Global Burden of Diseases, Injuries, and Risk Factors Study (GBD). GBD is a large-scale scientific effort to systematically quantify the levels of, and trends in, morbidity and mortality due to 371 diseases and injuries in 204 countries and territories.^26^, ^27^ GBD uses geo-temporal modelling tools and algorithms to estimate the disease burden in both data-rich and data-scarce locations. Annual iterations of burden estimation enable researchers to revisit model assumptions and update input data, data processing, and model parameters to reflect the evolving scientific understanding of disease epidemiology. The last GBD report on the global burden of appendicitis, based on results from GBD 2019, was published in 2023 by Guan and colleagues.^28^ The goal of the current report is to present the findings from GBD 2021. We aimed to summarise the incidence, mortality, years lived with disability (YLDs), years of life lost (YLLs), and disability-adjusted life-years (DALYs) due to appendicitis from 1990 to 2021 for 21 GBD regions, 204 countries and territories, and globally. This manuscript was produced as part of the GBD Collaborator Network and in accordance with the GBD Protocol.

## Methods

### Overview

The overall GBD study aims, data sources, and modelling approach and tools have been described elsewhere.^27^, ^29^, ^30^ Details of input data and the modelling framework pertaining specifically to appendicitis are summarised below. We defined appendicitis using the following International Classification of Diseases version 9 (ICD-9) and version 10 (ICD-10) codes: acute appendicitis (ICD-9: 540; ICD-10: K35), unspecified appendicitis (ICD-9: 541; ICD-10: K37), and other appendicitis (ICD-9: 542; ICD-10: K36). For mortality estimation, we also included the following ICD-10 codes: fistula of appendix (K38.3), other specified diseases of appendix (K38.8), and disease of appendix, unspecified (K38.9).

The analyses presented here comply with the Guidelines for Accurate and Transparent Health Estimates Reporting^31^ (GATHER; appendix table S1). The results are presented to three significant figures.

### Mortality estimation

Cause of death (CoD) data were extracted from vital registration systems and verbal autopsy records from more than 870 sources in 134 countries and territories. Each data source covers 1–36 years of data starting from Jan 1, 1980 (global data availability maps are included in the appendix figure S2). Detailed descriptions of the methods for mapping, processing, and standardising CoD data have been published previously.^27^ In brief, CoD data are adjusted for different coding practices, age-sex aggregations, and misclassification of deaths. They are then mapped to each GBD-defined disease via the ICD codes. Garbage codes—codes to which deaths were assigned in primary data sources that cannot or should not be considered as the underlying cause of death—were redistributed to GBD-defined diseases, including appendicitis.^32^ Last, we used a Bayesian noise reduction algorithm to address stochastic temporal or geographical trends that are commonly observed in data for rare causes of death or small sample sizes.

The processed CoD data were analysed with the Cause of Death Ensemble model (CODEm), which has been described in detail previously,^27^, ^33^, ^34^, ^35^, ^36^ and is summarised in the appendix (section 4.3). The predictive covariates that were tested for inclusion for appendicitis are age-sex-specific scaled exposure variables for low fruit consumption and low vegetable consumption, Healthcare Access and Quality Index (HAQ Index), Socio-demographic Index (SDI), education attainment, and lag-distributed income per capita (appendix table S4). The details of input data and modelling processes of these covariates in GBD have been published previously.^22^, ^37^, ^38^, ^39^ The outputs from CODEm were scaled to fit into the GBD estimates of all-cause mortality rates^38^ within each age, sex, year, and location combination through the cause of death correct procedure (CoDCorrect; see appendix 1 section 3.3.2 of the study by the GBD 2019 Diseases and Injuries Collaborators^27^).

### Incidence estimation

Incidence data were extracted from two main sources: health insurance claims from the USA, Poland, and Taiwan (province of China); and inpatient discharge records from 50 countries (global data availability maps are included in the appendix figure S4). Details of these data sources and their processing have been described in detail elsewhere.^27^ Briefly, insurance claims data for the USA were obtained from the Truven database of USA private health insurance. The dataset included more than 12 billion claims spanning 9 years (2000 and 2010–17). Insurance claims data from Poland and Taiwan (province of China) were obtained from national insurance programmes, with about 99% population coverage from Poland (2003–12) and 90% of population coverage from Taiwan (province of China; 2016).^23^, ^24^, ^25^ The benefit of using insurance claims data was that these data included claims from encounters in multiple settings (inpatient, hospital day services, outpatient clinics, and emergency rooms) tagged with multiple diagnosis codes per visit. Additionally, unique enrollee IDs allowed us to link multiple claims to a single individual and follow them up over time and across settings. An individual was extracted from insurance claims data as an incident case if the person had at least one encounter with an appropriate ICD code as any diagnosis in any setting. To ensure individuals who came in for follow-up visits were not double counted, subsequent encounters within 1 year from the first diagnosis were assumed to relate to a single episode.

Inpatient discharge data were obtained from 95 different sources in 50 countries, covering 1– 26 years of data, starting from as early as 1988 Jan 1, 1988. Most sources of inpatient data were indexed by encounter (not linked to individuals) and provided a code only for primary discharge diagnosis. Due to variability in admission and day-procedure practices and record-keeping across settings, we standardised data by restricting inpatient discharges to encounters with a minimum length of stay of 24 h. More details are provided in the appendix (section 5.2.1). Records of inpatient discharges with an appendicitis code as the primary diagnosis from facilities covering a defined catchment area were combined with population estimates for the corresponding year, age, sex, and location to directly obtain estimates of appendicitis inpatient discharge rates. For data from facilities not covering a defined location, inpatient discharges with an appendicitis code as a primary diagnosis were divided by the total inpatient discharges for each age, sex, year, and source to produce cause fractions. These cause fractions were then multiplied by estimates of the hospital admission rate per person^27^ for each unique source, age, sex, and year combination to estimate population-level rates of inpatient discharges for appendicitis. To capture cases with an appendicitis code applied as a non-primary diagnosis and cases diagnosed in an inpatient encounter lasting less than 24 h, in an emergency room or other non-inpatient hospital service, or in an outpatient encounter, we applied a modelled correction factor. The correction factor was obtained by calculating the ratio of total cases (diagnosed in any health-care encounter, with appendicitis code in any diagnostic position) to inpatient discharges with a primary diagnosis of appendicitis as observed in the health insurance claims data described above. These ratios were modelled as a function of age, sex, and HAQ Index^40^ by use of a mixed-effects model in the meta-regression—Bayesian, regularised, trimmed tool (MR-BRT).^37^, ^41^ Details of modelling correction factors for inpatient data used in GBD can be found in a previous GBD publication^27^ and in the appendix (section 5.2.1).

Specifically for appendicitis, we considered claims data from Poland and Taiwan (province of China) and inpatient discharge data (corrected as described above) as reference data. We considered claims data from the USA as non-reference data due to a systematic bias associated with commercial health insurance status. We adjusted these non-reference data towards reference data by matching the reference and non-reference datapoints by age, sex, and location within 5-year intervals; calculating differences in logit of incidence; and modelling the logit differences in MR-BRT as a function of age and sex. The reference data from the USA were the corrected inpatient discharge data from the Healthcare Cost and Utilization Project. Details of adjustment for commercial bias in appendicitis claims data are given in the appendix (pp 11–12).

Last, given the heterogeneity within the clinical administrative data, we also systematically excluded data series with age-standardised incidence rates greater or less than two median absolute deviations from the median of the age-standardised incidence rates of all data sources.

Cause-specific mortality rate (CSMR) estimates drawn from the fatal estimation process described above were used to estimate incidence. Excess mortality rates (EMRs)—the number of deaths due to appendicitis among those who have appendicitis—were estimated by matching CSMR estimates to adjusted incidence data points from claims and inpatient sources (both described above), dividing them, and modelling those ratios as a function of age, sex, and HAQ Index with MR-BRT. The modelling of EMR as an input to non-fatal estimation is described in the appendix (section 5.2.2).

We estimated the year-age-sex-location-specific incidence of appendicitis using the disease modelling meta-regression (DisMod-MR) tool, version 2.1.^27^, ^42^ DisMod-MR is a Bayesian mixed-effects meta-regression modelling tool that uses a compartmental framework to leverage all available epidemiological data and produce internally consistent incidence, prevalence, remission, and mortality estimates. It first fits a global model based on all data. The global estimates are then passed down to models for the seven GBD super-regions as Bayesian priors. This process is repeated serially down to the most detailed location. The estimates are then aggregated back up to the global level to produce the final incidence estimates. This Bayesian hierarchical approach allows the models to borrow strength from predictive covariates and regional levels estimated from locations with data to inform the estimates in locations where there are few or no data. In addition to the input data, we used a Bayesian prior on remission that reflects an average disease duration of 2 weeks. We also included fixed-effect covariates on incidence and EMR to capture geographical variations based on the level of fibre consumption and HAQ Index.

### Estimation of YLLs, YLDs, and DALYs

We calculated YLLs by multiplying the CoDCorrected number of deaths by the reference standard life expectancy at the age of death.^38^ We calculated YLDs by multiplying incident appendicitis cases by an average duration of 2 weeks and a disability weight of 0·32 (95% uncertainty interval [UI] 0·22–0·44).^43^, ^44^, ^45^ DALYs were calculated by summing YLLs and YLDs due to appendicitis.

### Uncertainty intervals

The 95% UIs of the final burden estimates were calculated by producing 500–1000 draws of the posterior distribution at every modelling step, carrying out draw-level calculations for any subsequent scaling, and reporting the draws corresponding to the 2·5th percentile and 97·5th percentile of the distribution for each quantity reported. Specifically, for fatal burden estimates, the uncertainty intervals take into account sampling error and the uncertainty of garbage code redistribution in the underlying CoD data, as well as the uncertainty from regression parameters and heterogeneity of submodels within CODEm and from all-cause mortality envelopes in CoDCorrect.^27^, ^33^ Similarly, for non-fatal burden estimates, the 95% UIs take into account sampling error in the underlying incidence data; uncertainty in model-fitting and between-study heterogeneity when estimating correction factors for incidence data, EMR inputs, and CSMR inputs; uncertainty in the regression parameters in DisMod; and uncertainty in data and modelling for disability weight and severity distributions. Covariates used in CODEm and DisMod are provided to the model as mean estimated values for each year-age-sex-location combination, without uncertainty, because it is not computationally feasible at present to bootstrap each covariate. After fatal and non-fatal estimation processes were complete, we performed final uncertainty propagation in the DALY estimation process. Age-sex-year-location-specific DALY estimates were calculated by summing draws of YLLs and YLDs to generate samples of DALY distribution. We assumed no correlation between the uncertainty in YLLs and YLDs, and the 95% UI is reported as the 2·5th percentile and 97·5th percentile of the distribution.

### Socio-demographic Index and Healthcare Access and Quality Index

The national socioeconomic development and access to quality health care were derived from GBD's SDI and HAQ Index estimates. SDI is a composite indicator of a country's lag-distributed income per capita, average years of schooling, and the total fertility rate in females younger than 25 years. The HAQ Index is estimated from national mortality rates of causes that are considered amenable to personal health-care access and quality. Details of input data and estimation processes for these two indicators have been described previously.^38^, ^40^

### Role of the funding source

The funder of this study had no role in study design, data collection, data analysis, data interpretation, the writing of the manuscript, or the decision to submit the manuscript for publication.

## Results

**Figure 1:**
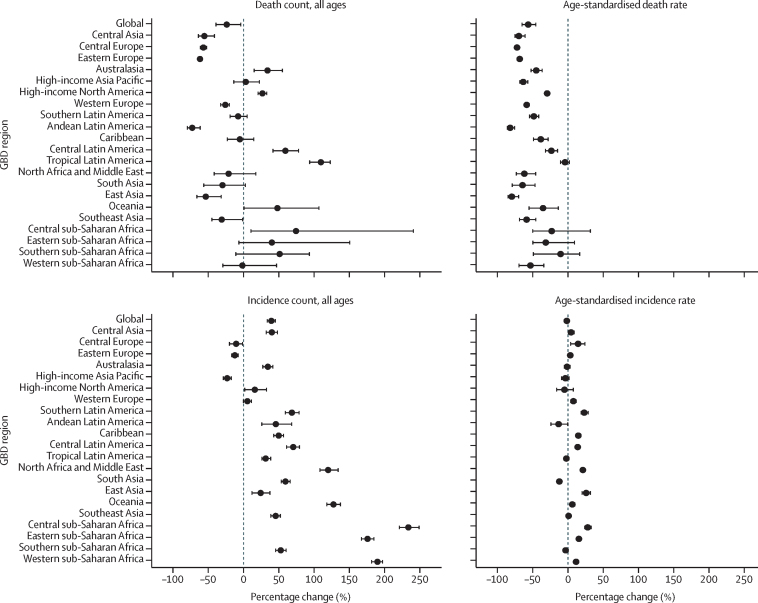
Percentage change in deaths and incidence due to appendicitis between 1990 and 2021, globally and for 21 GBD regions, for both sexes combined Circles represent the mean percentage change between 1990 and 2021. The lines represent the 95% uncertainty interval of the mean. GBD=Global Burden of Diseases, Injuries, and Risk Factors Study.

In 2021, there were an estimated 29 300 (95% UI 25 500 to 34 000) deaths from appendicitis worldwide, corresponding to a global age-standardised mortality rate of 0·358 (0·311 to 0·414) per 100 000 (table 1). Mortality rates ranged from 1·01 (0·895 to 1·13) per 100 000 in central Latin America to 0·0540 (0·0464 to 0·0617) per 100 000 in high-income Asia Pacific. The global age-standardised mortality rates halved between 1990 and 2021 (–57·3% [–66·5 to –46·0]), and a downward trend was seen in all 21 GBD regions (figure 1). The biggest reduction was observed in Andean Latin America (–83·1% [–87·3 to –76·3]), followed by east Asia (–80·7% [–86·3 to –71·0]) and central Europe (–73·2% [–75·9 to –70·1]). Absolute number of deaths, however, decreased in only seven of 21 GBD regions: Andean Latin America, eastern Europe, central Europe, central Asia, east Asia, southeast Asia, and western Europe.

**Table 1:** Global, super-regional, regional, and national mortality due to appendicitis in 1990 and 2021

		**Death counts in 1990 (95% UI)**	**Age-standardised mortality rates per 100 000 in 1990 (95% UI)**	**Death counts in 2021 (95% UI)**	**Age-standardised mortality rates per 100 000 in 2021 (95% UI)**	**Percentage change in death counts between 1990 and 2021, % (95% UI)**	**Percentage change in age-standardised mortality rates between 1990 and 2021, % (95% UI)**
**Global**		**38 700 (30 800 to 49 100)**	**0·838 (0·672 to 1·08)**	**29 300 (25 500 to 34 000)**	**0·358 (0·311 to 0·414)**	**−24·1% (−40·0 to −2·96)**	**−57·3% (−66·5 to −46·0)**
**Central Europe, eastern Europe, and central Asia**		**2190 (2090 to 2270)**	**0·489 (0·465 to 0·509)**	**878 (810 to 967)**	**0·149 (0·137 to 0·167)**	**−59·9% (−62·7 to −56·4)**	**−69·5% (−71·7 to −66·3)**
Central Asia		304 (274 to 332)	0·511 (0·461 to 0·555)	134 (111 to 181)	0·150 (0·125 to 0·198)	−55·8% (−64·2 to −42·6)	−70·5% (−75·9 to −62·3)
	Armenia	14·1 (11·9 to 16·7)	0·463 (0·387 to 0·547)	4·78 (3·79 to 6·02)	0·121 (0·0967 to 0·152)	−66·2% (−74·9 to −53·9)	−73·8% (−80·4 to −64·2)
	Azerbaijan	35·0 (26·3 to 43·3)	0·557 (0·414 to 0·691)	12·6 (7·97 to 21·3)	0·122 (0·0782 to 0·204)	−64·0% (−77·7 to −39·3)	−78·1% (−86·4 to −62·8)
	Georgia	15·1 (13·6 to 16·6)	0·255 (0·230 to 0·280)	5·09 (3·80 to 6·61)	0·0974 (0·0731 to 0·126)	−66·3% (−75·1 to −54·9)	−61·8% (−71·7 to −48·6)
	Kazakhstan	72·0 (61·2 to 83·9)	0·496 (0·418 to 0·580)	22·4 (18·2 to 29·6)	0·119 (0·0966 to 0·155)	−68·9% (−76·8 to −57·8)	−76·1% (−82·1 to −67·7)
	Kyrgyzstan	23·5 (20·6 to 26·8)	0·637 (0·557 to 0·724)	8·56 (6·87 to 10·6)	0·149 (0·119 to 0·185)	−63·7% (−71·9 to −53·3)	−76·6% (−81·9 to −70·0)
	Mongolia	17·6 (9·91 to 26·8)	1·10 (0·641 to 1·69)	10·6 (7·31 to 14·9)	0·390 (0·270 to 0·537)	−39·5% (−64·7 to 11·8)	−64·6% (−78·6 to −36·7)
	Tajikistan	34·1 (21·4 to 46·8)	0·700 (0·464 to 0·935)	27·5 (15·3 to 59·0)	0·314 (0·180 to 0·619)	−19·4% (−56·4 to 64·3)	−51·1% (−71·8 to −8·41)
	Turkmenistan	16·7 (15·0 to 18·7)	0·579 (0·514 to 0·658)	7·16 (5·50 to 9·31)	0·151 (0·115 to 0·194)	−57·1% (−68·1 to −43·3)	−74·0% (−80·8 to −65·1)
	Uzbekistan	76·1 (65·3 to 88·0)	0·453 (0·389 to 0·522)	35·7 (27·4 to 46·4)	0·119 (0·0914 to 0·154)	−53·1% (−65·8 to −37·3)	−73·7% (−80·7 to −64·9)
Central Europe		613 (577 to 647)	0·451 (0·424 to 0·477)	262 (235 to 291)	0·121 (0·109 to 0·135)	−57·3% (−61·6 to −52·4)	−73·2% (−75·9 to −70·1)
	Albania	7·04 (4·99 to 9·55)	0·321 (0·228 to 0·436)	3·16 (2·05 to 4·73)	0·0825 (0·0538 to 0·123)	−55·1% (−73·2 to −26·9)	−74·3% (−84·5 to −58·4)
	Bosnia and Herzegovina	20·8 (14·8 to 28·0)	0·541 (0·382 to 0·729)	8·15 (5·33 to 12·2)	0·136 (0·0889 to 0·205)	−60·7% (−76·4 to −35·5)	−74·9% (−85·0 to −58·2)
	Bulgaria	48·1 (41·0 to 55·8)	0·482 (0·415 to 0·556)	23·5 (17·9 to 31·3)	0·177 (0·135 to 0·234)	−51·2% (−64·6 to −31·4)	−63·3% (−73·3 to −49·2)
	Croatia	22·0 (19·0 to 25·5)	0·407 (0·351 to 0·470)	9·67 (7·40 to 12·3)	0·106 (0·0821 to 0·133)	−56·0% (−67·2 to −43·2)	−74·0% (−80·4 to −66·7)
	Czechia	63·9 (56·6 to 72·1)	0·485 (0·432 to 0·544)	24·8 (19·8 to 30·0)	0·115 (0·0945 to 0·138)	−61·1% (−69·3 to −51·5)	−76·2% (−81·0 to −70·6)
	Hungary	78·2 (67·3 to 90·3)	0·583 (0·502 to 0·677)	30·8 (23·5 to 39·9)	0·159 (0·122 to 0·206)	−60·6% (−70·7 to −47·5)	−72·7% (−79·7 to −63·7)
	Montenegro	0·515 (0·357 to 0·730)	0·0840 (0·0585 to 0·119)	0·493 (0·337 to 0·715)	0·0556 (0·0381 to 0·0806)	−4·23% (−42·7 to 54·0)	−33·8% (−60·2 to 5·37)
	North Macedonia	2·78 (1·96 to 3·79)	0·156 (0·109 to 0·212)	2·10 (1·40 to 3·15)	0·0767 (0·0526 to 0·112)	−24·5% (−52·8 to 17·2)	−50·9% (−68·8 to −25·1)
	Poland	205 (191 to 218)	0·505 (0·468 to 0·538)	75·5 (67·0 to 83·6)	0·106 (0·0950 to 0·117)	−63·2% (−67·2 to −58·8)	−78·9% (−81·2 to −76·6)
	Romania	96·0 (81·7 to 111)	0·392 (0·335 to 0·454)	40·9 (30·8 to 54·2)	0·116 (0·0875 to 0·154)	−57·4% (−68·6 to −40·0)	−70·3% (−78·1 to −58·5)
	Serbia	34·5 (25·0 to 47·3)	0·368 (0·266 to 0·506)	24·9 (18·0 to 33·0)	0·152 (0·112 to 0·201)	−27·9% (−52·4 to 12·4)	−58·7% (−72·7 to −34·6)
	Slovakia	15·5 (11·4 to 20·1)	0·270 (0·198 to 0·346)	10·0 (7·26 to 13·9)	0·112 (0·0812 to 0·156)	−35·2% (−55·5 to −0·251)	−58·6% (−71·6 to −36·4)
	Slovenia	8·49 (7·13 to 10·1)	0·359 (0·303 to 0·428)	3·94 (2·89 to 5·01)	0·0828 (0·0613 to 0·106)	−53·6% (−66·0 to −38·2)	−76·9% (−82·8 to −69·4)
Eastern Europe		1270 (1210 to 1330)	0·489 (0·465 to 0·511)	482 (444 to 526)	0·149 (0·138 to 0·163)	−62·1% (−65·0 to −58·8)	−69·5% (−71·8 to −66·9)
	Belarus	46·3 (40·1 to 53·8)	0·382 (0·331 to 0·444)	19·5 (15·0 to 25·1)	0·131 (0·102 to 0·168)	−58·0% (−68·5 to −44·6)	−65·6% (−74·1 to −55·0)
	Estonia	7·72 (6·51 to 9·11)	0·407 (0·343 to 0·477)	1·74 (1·32 to 2·28)	0·0707 (0·0539 to 0·0927)	−77·5% (−83·8 to −69·3)	−82·6% (−87·4 to −76·3)
	Latvia	16·2 (13·7 to 19·3)	0·491 (0·415 to 0·585)	3·99 (2·96 to 5·35)	0·112 (0·0834 to 0·150)	−75·3% (−82·6 to −65·0)	−77·3% (−83·8 to −67·6)
	Lithuania	20·2 (17·5 to 23·0)	0·475 (0·417 to 0·538)	7·11 (5·38 to 9·33)	0·134 (0·104 to 0·171)	−64·8% (−74·3 to −52·9)	−71·9% (−78·8 to −63·1)
	Moldova	27·6 (22·9 to 32·6)	0·655 (0·547 to 0·772)	8·53 (6·22 to 11·3)	0·158 (0·116 to 0·208)	−69·1% (−78·1 to −57·8)	−75·9% (−82·9 to −67·3)
	Russia	874 (827 to 916)	0·516 (0·488 to 0·541)	382 (351 to 417)	0·172 (0·158 to 0·188)	−56·3% (−59·9 to −52·5)	−66·7% (−69·3 to −63·9)
	Ukraine	279 (255 to 304)	0·435 (0·399 to 0·473)	59·3 (44·1 to 77·3)	0·0911 (0·0683 to 0·118)	−78·7% (−84·3 to −71·6)	−79·1% (−84·5 to −72·4)
**High income**		**2310 (2170 to 2420)**	**0·204 (0·192 to 0·213)**	**2130 (1880 to 2310)**	**0·0976 (0·0883 to 0·105)**	**−7·74% (−14·5 to −2·50)**	**−52·1% (−54·6 to −49·8)**
Australasia		34·7 (31·3 to 38·0)	0·153 (0·138 to 0·168)	46·4 (38·9 to 53·0)	0·0830 (0·0704 to 0·0945)	33·6% (14·0 to 55·1)	−45·9% (−53·8 to −37·1)
	Australia	29·3 (26·2 to 32·3)	0·155 (0·139 to 0·171)	36·9 (30·4 to 42·9)	0·0780 (0·0652 to 0·0903)	26·0% (5·40 to 48·4)	−49·7% (−57·7 to −40·8)
	New Zealand	5·44 (4·84 to 6·06)	0·144 (0·128 to 0·160)	9·49 (7·97 to 10·9)	0·110 (0·0934 to 0·126)	74·5% (47·3 to 106)	−23·3% (−34·8 to −9·93)
High-income Asia Pacific		269 (228 to 297)	0·152 (0·129 to 0·168)	277 (223 to 319)	0·0540 (0·0464 to 0·0617)	2·89% (−14·9 to 23·3)	−64·4% (−69·7 to −57·6)
	Brunei	0·358 (0·248 to 0·475)	0·260 (0·185 to 0·349)	0·513 (0·397 to 0·656)	0·166 (0·127 to 0·210)	43·3% (0·653 to 129)	−36·0% (−55·0 to −1·78)
	Japan	112 (103 to 118)	0·0743 (0·0679 to 0·0781)	184 (147 to 208)	0·0438 (0·0381 to 0·0483)	64·7% (43·1 to 80·3)	−41·1% (−45·0 to −37·2)
	Singapore	3·97 (3·58 to 4·36)	0·178 (0·160 to 0·197)	3·53 (2·92 to 4·10)	0·0461 (0·0383 to 0·0534)	−11·0% (−26·2 to 5·31)	−74·2% (−78·3 to −69·5)
	South Korea	153 (112 to 180)	0·547 (0·411 to 0·658)	88·6 (64·7 to 118)	0·106 (0·0766 to 0·140)	−42·1% (−57·8 to −18·8)	−80·7% (−85·9 to −72·8)
High-income North America		538 (501 to 563)	0·158 (0·148 to 0·165)	680 (607 to 728)	0·110 (0·100 to 0·117)	26·5% (19·9 to 32·5)	−30·3% (−33·3 to −27·3)
	Canada	47·8 (42·6 to 53·3)	0·153 (0·137 to 0·169)	62·1 (52·1 to 72·1)	0·0879 (0·0747 to 0·101)	29·9% (10·6 to 53·4)	−42·7% (−50·9 to −33·0)
	Greenland	0·0720 (0·0526 to 0·100)	0·196 (0·142 to 0·261)	0·137 (0·0414 to 0·194)	0·208 (0·0656 to 0·294)	90·2% (−51·1 to 251)	6·13% (−66·5 to 92·8)
	USA	490 (455 to 514)	0·159 (0·148 to 0·166)	618 (553 to 661)	0·113 (0·103 to 0·120)	26·2% (19·4 to 32·2)	−28·9% (−32·2 to −25·9)
Southern Latin America		241 (225 to 257)	0·523 (0·487 to 0·558)	222 (195 to 250)	0·265 (0·234 to 0·300)	−8·06% (−19·2 to 4·56)	−49·3% (−55·3 to −42·5)
	Argentina	150 (139 to 162)	0·475 (0·439 to 0·511)	139 (122 to 159)	0·257 (0·227 to 0·293)	−7·56% (−19·5 to 6·29)	−45·9% (−52·8 to −38·0)
	Chile	72·4 (66·3 to 79·0)	0·675 (0·617 to 0·737)	67·3 (57·5 to 77·5)	0·277 (0·238 to 0·318)	−7·11% (−21·3 to 8·94)	−58·9% (−65·0 to −51·9)
	Uruguay	18·4 (16·8 to 20·1)	0·506 (0·464 to 0·552)	15·4 (13·4 to 17·6)	0·300 (0·261 to 0·340)	−15·9% (−27·5 to −2·13)	−40·8% (−48·7 to −31·3)
Western Europe		1230 (1140 to 1300)	0·219 (0·203 to 0·231)	908 (780 to 1000)	0·0887 (0·0785 to 0·0969)	−26·2% (−32·7 to −20·1)	−59·5% (−62·5 to −56·4)
	Andorra	0·0776 (0·0445 to 0·116)	0·159 (0·0922 to 0·236)	0·110 (0·0739 to 0·152)	0·0676 (0·0453 to 0·0932)	41·6% (−16·1 to 169)	−57·6% (−74·2 to −22·0)
	Austria	27·1 (24·3 to 29·7)	0·234 (0·210 to 0·255)	15·8 (13·3 to 18·1)	0·0797 (0·0680 to 0·0910)	−41·7% (−50·2 to −32·2)	−65·9% (−70·6 to −60·5)
	Belgium	24·0 (21·1 to 26·8)	0·161 (0·142 to 0·179)	16·3 (13·5 to 18·9)	0·0633 (0·0539 to 0·0733)	−32·0% (−42·3 to −20·6)	−60·7% (−66·5 to −54·3)
	Cyprus	1·57 (0·832 to 2·52)	0·278 (0·153 to 0·468)	1·25 (0·932 to 1·64)	0·0701 (0·0531 to 0·0929)	−20·4% (−56·2 to 55·0)	−74·8% (−86·4 to −52·7)
	Denmark	29·7 (26·6 to 32·9)	0·375 (0·339 to 0·414)	23·4 (19·7 to 27·4)	0·184 (0·156 to 0·216)	−21·4% (−33·4 to −6·61)	−50·9% (−58·1 to −41·9)
	Finland	16·7 (14·7 to 18·7)	0·243 (0·215 to 0·272)	12·3 (10·2 to 14·5)	0·0922 (0·0778 to 0·108)	−26·1% (−38·3 to −10·7)	−62·0% (−68·0 to −54·8)
	France	209 (189 to 228)	0·247 (0·224 to 0·267)	140 (116 to 163)	0·0862 (0·0734 to 0·100)	−33·0% (−42·2 to −22·2)	−65·1% (−69·8 to −59·7)
	Germany	391 (347 to 429)	0·311 (0·280 to 0·340)	214 (180 to 251)	0·102 (0·0881 to 0·118)	−45·2% (−53·4 to −35·2)	−67·1% (−71·6 to −61·4)
	Greece	14·3 (12·7 to 15·9)	0·101 (0·0906 to 0·112)	14·5 (12·2 to 16·7)	0·0507 (0·0434 to 0·0584)	1·29% (−14·0 to 19·1)	−49·7% (−56·9 to −40·8)
	Iceland	0·359 (0·318 to 0·398)	0·124 (0·111 to 0·138)	0·287 (0·242 to 0·335)	0·0483 (0·0414 to 0·0560)	−20·1% (−32·2 to −5·92)	−61·1% (−66·8 to −54·5)
	Ireland	6·18 (5·51 to 6·96)	0·156 (0·139 to 0·175)	3·70 (3·05 to 4·44)	0·0462 (0·0386 to 0·0553)	−40·1% (−50·6 to −26·9)	−70·4% (−75·4 to −63·9)
	Israel	7·36 (6·59 to 8·21)	0·159 (0·141 to 0·178)	7·92 (6·53 to 9·28)	0·0597 (0·0501 to 0·0698)	7·70% (−10·5 to 28·1)	−62·4% (−68·4 to −55·8)
	Italy	105 (96·4 to 111)	0·129 (0·119 to 0·136)	85·7 (71·3 to 96·0)	0·0528 (0·0454 to 0·0587)	−18·3% (−26·8 to −10·8)	−59·0% (−62·4 to −55·4)
	Luxembourg	1·45 (1·29 to 1·62)	0·285 (0·254 to 0·318)	1·13 (0·920 to 1·35)	0·0989 (0·0816 to 0·119)	−22·2% (−36·5 to −5·73)	−65·3% (−71·5 to −57·9)
	Malta	0·594 (0·520 to 0·673)	0·148 (0·130 to 0·167)	0·557 (0·446 to 0·670)	0·0581 (0·0475 to 0·0691)	−6·12% (−25·1 to 14·8)	−60·7% (−68·2 to −52·4)
	Monaco	0·0419 (0·0290 to 0·0555)	0·0600 (0·0419 to 0·0789)	0·0362 (0·0270 to 0·0474)	0·0357 (0·0269 to 0·0463)	−13·5% (−37·7 to 22·6)	−40·4% (−56·6 to −16·5)
	Netherlands	48·6 (43·1 to 53·6)	0·248 (0·222 to 0·273)	39·4 (33·0 to 45·2)	0·105 (0·0894 to 0·121)	−18·9% (−30·5 to −6·70)	−57·5% (−63·5 to −51·3)
	Norway	11·2 (10·2 to 12·0)	0·158 (0·145 to 0·169)	10·1 (8·35 to 11·5)	0·0890 (0·0758 to 0·101)	−9·79% (−20·2 to 1·37)	−43·5% (−49·6 to −36·8)
	Portugal	30·3 (27·4 to 33·2)	0·248 (0·225 to 0·273)	24·4 (20·6 to 28·1)	0·0903 (0·0777 to 0·103)	−19·6% (−32·1 to −4·58)	−63·6% (−68·9 to −57·7)
	San Marino	0·0690 (0·0507 to 0·0922)	0·193 (0·143 to 0·255)	0·0698 (0·0421 to 0·100)	0·0699 (0·0430 to 0·101)	1·18% (−36·9 to 50·1)	−63·7% (−76·8 to −45·9)
	Spain	114 (103 to 124)	0·222 (0·201 to 0·242)	102 (83·2 to 122)	0·0907 (0·0760 to 0·106)	−10·0% (−25·9 to 8·38)	−59·1% (−65·5 to −51·8)
	Sweden	25·6 (22·8 to 28·4)	0·168 (0·150 to 0·184)	18·8 (15·4 to 22·1)	0·0753 (0·0630 to 0·0882)	−26·6% (−38·5 to −14·0)	−55·1% (−62·2 to −47·6)
	Switzerland	19·7 (17·6 to 21·8)	0·192 (0·173 to 0·212)	18·0 (14·6 to 21·7)	0·0863 (0·0719 to 0·104)	−8·72% (−24·3 to 9·98)	−55·1% (−62·5 to −46·4)
	UK	146 (138 to 151)	0·169 (0·160 to 0·175)	157 (140 to 168)	0·118 (0·107 to 0·126)	7·96% (0·812 to 13·7)	−30·0% (−34·3 to −26·6)
**Latin America and Caribbean**		**4620 (4110 to 5080)**	**1·41 (1·28 to 1·53)**	**5000 (4570 to 5470)**	**0·822 (0·750 to 0·902)**	**8·23% (−4·39 to 23·1)**	**−41·7% (−47·8 to −34·9)**
Andean Latin America		1850 (1440 to 2230)	4·74 (3·76 to 5·66)	499 (395 to 640)	0·804 (0·637 to 1·03)	−73·1% (−80·0 to −60·7)	−83·1% (−87·3 to −76·3)
	Bolivia	302 (114 to 482)	4·73 (1·85 to 7·31)	140 (85·9 to 209)	1·37 (0·814 to 2·05)	−53·7% (−71·7 to −2·34)	−71·0% (−81·2 to −42·5)
	Ecuador	314 (290 to 340)	3·53 (3·27 to 3·80)	121 (95·0 to 150)	0·718 (0·564 to 0·884)	−61·5% (−69·7 to −51·7)	−79·7% (−84·0 to −74·6)
	Peru	1240 (848 to 1550)	5·34 (3·83 to 6·57)	238 (171 to 330)	0·683 (0·491 to 0·947)	−80·8% (−87·5 to −65·8)	−87·2% (−91·5 to −78·9)
Caribbean		431 (324 to 531)	1·35 (1·05 to 1·62)	407 (322 to 505)	0·817 (0·637 to 1·03)	−5·51% (−23·1 to 16·5)	−39·3% (−49·8 to −27·4)
	Antigua and Barbuda	0·417 (0·371 to 0·463)	0·738 (0·659 to 0·821)	0·534 (0·478 to 0·593)	0·551 (0·492 to 0·613)	28·2% (10·0 to 48·1)	−25·3% (−35·6 to −14·0)
	The Bahamas	1·34 (1·19 to 1·50)	0·679 (0·602 to 0·764)	1·86 (1·45 to 2·34)	0·469 (0·367 to 0·592)	38·7% (7·13 to 80·6)	−30·9% (−46·6 to −9·94)
	Barbados	1·57 (1·41 to 1·75)	0·567 (0·508 to 0·630)	1·26 (0·940 to 1·61)	0·279 (0·210 to 0·359)	−19·9% (−41·0 to 3·62)	−50·8% (−63·8 to −35·5)
	Belize	1·11 (0·984 to 1·25)	0·674 (0·604 to 0·750)	1·76 (1·49 to 2·08)	0·515 (0·432 to 0·612)	58·5% (28·1 to 94·7)	−23·5% (−37·6 to −7·21)
	Bermuda	0·350 (0·317 to 0·385)	0·588 (0·532 to 0·644)	0·294 (0·240 to 0·368)	0·245 (0·201 to 0·305)	−16·0% (−32·9 to 7·75)	−58·4% (−66·8 to −46·6)
	Cuba	96·8 (89·6 to 105)	0·943 (0·870 to 1·02)	119 (100 to 140)	0·670 (0·567 to 0·792)	22·6% (2·60 to 46·5)	−28·9% (−40·6 to −15·3)
	Dominica	0·510 (0·428 to 0·592)	0·813 (0·678 to 0·948)	0·382 (0·280 to 0·484)	0·512 (0·379 to 0·647)	−25·2% (−44·9 to −1·47)	−37·0% (−53·8 to −17·8)
	Dominican Republic	111 (74·4 to 136)	1·69 (1·15 to 2·02)	67·6 (41·3 to 97·1)	0·650 (0·396 to 0·931)	−39·1% (−59·8 to −10·5)	−61·6% (−74·7 to −44·9)
	Grenada	0·465 (0·408 to 0·531)	0·582 (0·509 to 0·661)	0·400 (0·338 to 0·467)	0·386 (0·327 to 0·446)	−13·9% (−29·8 to 3·86)	−33·7% (−45·4 to −20·4)
	Guyana	11·9 (10·3 to 13·5)	2·16 (1·90 to 2·45)	9·29 (6·93 to 12·4)	1·36 (1·02 to 1·78)	−21·8% (−42·4 to 5·78)	−37·2% (−53·9 to −15·9)
	Haiti	151 (54·6 to 240)	2·72 (1·12 to 4·13)	160 (76·2 to 249)	1·66 (0·862 to 2·61)	6·11% (−29·4 to 93·1)	−39·1% (−57·2 to −4·89)
	Jamaica	13·6 (12·2 to 15·3)	0·645 (0·581 to 0·718)	11·1 (8·32 to 14·4)	0·363 (0·272 to 0·471)	−18·6% (−38·4 to 6·55)	−43·7% (−57·4 to −25·8)
	Puerto Rico	10·8 (9·92 to 11·8)	0·308 (0·283 to 0·337)	7·31 (6·00 to 8·80)	0·117 (0·0971 to 0·140)	−32·4% (−45·4 to −16·5)	−62·2% (−69·4 to −53·6)
	Saint Kitts and Nevis	0·470 (0·424 to 0·519)	1·26 (1·14 to 1·40)	0·396 (0·321 to 0·478)	0·645 (0·528 to 0·767)	−15·7% (−32·5 to 5·03)	−49·0% (−58·9 to −36·4)
	Saint Lucia	1·04 (0·952 to 1·14)	1·06 (0·969 to 1·15)	1·19 (0·960 to 1·45)	0·555 (0·447 to 0·680)	14·2% (−9·48 to 41·8)	−47·6% (−58·1 to −35·6)
	Saint Vincent and the Grenadines	0·502 (0·445 to 0·563)	0·577 (0·514 to 0·648)	0·591 (0·497 to 0·696)	0·459 (0·386 to 0·541)	17·7% (−2·69 to 45·6)	−20·5% (−34·3 to −2·38)
	Suriname	3·38 (2·40 to 3·98)	1·06 (0·760 to 1·24)	3·00 (2·20 to 3·95)	0·500 (0·368 to 0·657)	−11·3% (−36·4 to 31·2)	−52·9% (−66·1 to −32·2)
	Trinidad and Tobago	8·96 (8·14 to 9·85)	0·932 (0·845 to 1·03)	6·88 (5·15 to 8·86)	0·399 (0·301 to 0·512)	−23·2% (−43·5 to 0·987)	−57·2% (−68·5 to −43·9)
	Virgin Islands	0·674 (0·538 to 0·856)	0·733 (0·586 to 0·929)	0·366 (0·248 to 0·530)	0·303 (0·215 to 0·434)	−45·6% (−64·5 to −19·8)	−58·7% (−71·9 to −40·2)
Central Latin America		1580 (1510 to 1650)	1·33 (1·28 to 1·39)	2510 (2220 to 2820)	1·01 (0·895 to 1·13)	59·0% (40·3 to 77·9)	−24·3% (−33·0 to −15·3)
	Colombia	227 (209 to 244)	1·01 (0·927 to 1·10)	368 (306 to 436)	0·686 (0·569 to 0·815)	62·3% (33·6 to 94·8)	−32·3% (−44·1 to −18·7)
	Costa Rica	13·0 (11·8 to 14·4)	0·620 (0·560 to 0·687)	30·2 (26·0 to 34·8)	0·565 (0·486 to 0·648)	132% (95·3 to 174)	−8·96% (−23·8 to 7·38)
	El Salvador	93·7 (75·7 to 113)	2·06 (1·72 to 2·47)	53·5 (39·1 to 69·0)	0·836 (0·612 to 1·08)	−43·0% (−60·1 to −22·1)	−59·5% (−71·5 to −45·5)
	Guatemala	311 (286 to 340)	4·19 (3·90 to 4·50)	225 (187 to 264)	1·78 (1·48 to 2·10)	−27·7% (−40·2 to −14·3)	−57·4% (−64·3 to −49·7)
	Honduras	99·5 (62·2 to 143)	2·61 (1·73 to 3·89)	119 (69·4 to 175)	1·69 (0·962 to 2·48)	19·5% (−22·7 to 78·2)	−35·5% (−61·0 to −4·74)
	Mexico	587 (565 to 612)	1·11 (1·06 to 1·16)	1410 (1240 to 1570)	1·13 (0·994 to 1·25)	140% (111 to 168)	1·39% (−10·3 to 13·2)
	Nicaragua	34·2 (27·8 to 42·1)	1·14 (0·942 to 1·40)	32·4 (23·4 to 41·7)	0·613 (0·448 to 0·776)	−5·37% (−33·2 to 32·1)	−46·4% (−61·8 to −27·2)
	Panama	15·7 (13·9 to 17·6)	0·852 (0·752 to 0·960)	23·6 (18·4 to 28·9)	0·536 (0·418 to 0·654)	50·3% (15·5 to 86·0)	−37·1% (−51·7 to −22·4)
	Venezuela	197 (185 to 210)	1·44 (1·34 to 1·54)	251 (178 to 335)	0·884 (0·627 to 1·18)	27·1% (−11·1 to 69·9)	−38·4% (−57·0 to −18·6)
Tropical Latin America		756 (718 to 791)	0·669 (0·631 to 0·704)	1580 (1470 to 1680)	0·636 (0·590 to 0·677)	109% (94·1 to 123)	−4·81% (−11·2 to 1·42)
	Brazil	726 (689 to 759)	0·661 (0·623 to 0·695)	1530 (1420 to 1630)	0·631 (0·584 to 0·672)	111% (94·8 to 125)	−4·45% (−11·0 to 1·70)
	Paraguay	30·1 (24·4 to 37·1)	0·936 (0·760 to 1·19)	52·1 (37·4 to 70·0)	0·851 (0·609 to 1·14)	72·9% (17·0 to 146)	−9·14% (−39·4 to 30·0)
**North Africa and Middle East**		**1080 (669 to 1490)**	**0·496 (0·294 to 0·751)**	**845 (576 to 1120)**	**0·184 (0·123 to 0·244)**	**−21·5**%**(−41·8 to 18·7)**	**−62·8**%**(−74·4 to −45·1)**
North Africa and Middle East		1080 (669 to 1490)	0·496 (0·294 to 0·751)	845 (576 to 1120)	0·184 (0·123 to 0·244)	−21·5% (−41·8 to 18·7)	−62·8% (−74·4 to −45·1)
	Afghanistan	123 (52·4 to 212)	1·75 (0·748 to 3·17)	134 (67·1 to 211)	0·912 (0·453 to 1·46)	8·91% (−32·4 to 75·1)	−47·8% (−69·8 to −15·2)
	Algeria	78·5 (46·1 to 109)	0·542 (0·308 to 0·753)	64·9 (36·1 to 100)	0·206 (0·113 to 0·307)	−17·4% (−47·6 to 34·6)	−62·1% (−75·7 to −42·5)
	Bahrain	0·823 (0·640 to 1·08)	0·452 (0·348 to 0·596)	1·39 (0·939 to 2·18)	0·202 (0·133 to 0·316)	69·4% (1·21 to 188)	−55·3% (−73·6 to −22·2)
	Egypt	111 (68·2 to 134)	0·327 (0·181 to 0·393)	48·2 (35·3 to 67·4)	0·0782 (0·0573 to 0·108)	−56·4% (−70·1 to −26·0)	−76·1% (−83·4 to −57·1)
	Iran	223 (167 to 327)	0·631 (0·464 to 1·01)	125 (97·6 to 172)	0·171 (0·133 to 0·233)	−44·1% (−64·9 to −10·6)	−72·8% (−83·3 to −59·3)
	Iraq	11·8 (6·41 to 17·7)	0·0929 (0·0508 to 0·149)	10·2 (7·18 to 13·9)	0·0376 (0·0267 to 0·0503)	−13·7% (−50·4 to 85·8)	−59·6% (−80·2 to −15·4)
	Jordan	4·67 (2·74 to 6·50)	0·257 (0·157 to 0·366)	5·49 (3·87 to 7·84)	0·0710 (0·0501 to 0·100)	17·6% (−30·1 to 140)	−72·4% (−83·7 to −45·4)
	Kuwait	0·862 (0·750 to 1·02)	0·0944 (0·0825 to 0·110)	1·33 (1·08 to 1·64)	0·0445 (0·0355 to 0·0543)	54·3% (22·7 to 91·6)	−52·9% (−62·2 to −42·1)
	Lebanon	3·76 (1·65 to 5·92)	0·174 (0·0765 to 0·275)	3·85 (2·96 to 4·88)	0·0607 (0·0466 to 0·0771)	2·48% (−37·7 to 154)	−65·1% (−78·9 to −15·0)
	Libya	13·5 (5·68 to 30·5)	0·549 (0·219 to 1·30)	14·2 (6·53 to 23·8)	0·277 (0·121 to 0·476)	4·92% (−61·6 to 119)	−49·5% (−81·4 to 7·02)
	Morocco	118 (62·4 to 195)	0·673 (0·343 to 1·15)	93·8 (54·4 to 128)	0·297 (0·169 to 0·404)	−20·7% (−47·6 to 15·1)	−55·9% (−71·8 to −36·3)
	Oman	2·29 (1·38 to 4·17)	0·227 (0·133 to 0·448)	1·38 (0·909 to 2·35)	0·0607 (0·0389 to 0·0921)	−39·8% (−65·6 to 27·8)	−73·2% (−85·7 to −46·7)
	Palestine	7·28 (3·01 to 14·9)	0·751 (0·282 to 1·54)	4·55 (2·08 to 9·35)	0·189 (0·0811 to 0·361)	−37·5% (−66·0 to 3·22)	−74·8% (−87·9 to −56·6)
	Qatar	0·654 (0·449 to 0·917)	0·501 (0·345 to 0·724)	1·02 (0·667 to 1·68)	0·111 (0·0720 to 0·183)	55·7% (−8·28 to 205)	−77·9% (−88·3 to −58·9)
	Saudi Arabia	20·6 (11·5 to 40·3)	0·239 (0·129 to 0·515)	21·3 (13·6 to 32·8)	0·0863 (0·0571 to 0·132)	3·67% (−52·0 to 120)	−64·0% (−83·1 to −22·9)
	Sudan	122 (45·1 to 228)	0·912 (0·302 to 2·09)	110 (45·6 to 236)	0·428 (0·173 to 0·959)	−9·87% (−47·2 to 76·3)	−53·1% (−71·6 to −14·3)
	Syria	42·2 (27·6 to 57·5)	0·530 (0·356 to 0·765)	28·9 (19·0 to 43·3)	0·253 (0·167 to 0·373)	−31·4% (−62·8 to 36·8)	−52·2% (−74·8 to −8·39)
	Tunisia	23·0 (11·9 to 36·5)	0·422 (0·206 to 0·700)	18·9 (8·96 to 33·0)	0·160 (0·0740 to 0·279)	−18·0% (−51·9 to 31·6)	−62·0% (−77·4 to −39·8)
	Türkiye	110 (57·3 to 175)	0·263 (0·139 to 0·425)	73·7 (54·9 to 96·2)	0·0854 (0·0635 to 0·112)	−32·7% (−62·9 to 49·7)	−67·5% (−82·1 to −29·1)
	United Arab Emirates	1·39 (0·653 to 2·22)	0·193 (0·0889 to 0·332)	3·06 (2·11 to 4·74)	0·0953 (0·0641 to 0·139)	120% (25·9 to 435)	−50·6% (−72·7 to 27·8)
	Yemen	57·6 (22·3 to 114)	0·854 (0·285 to 2·19)	79·4 (36·6 to 127)	0·479 (0·213 to 0·793)	37·7% (−21·4 to 155)	−43·8% (−73·1 to 4·05)
**South Asia**		**18 500 (13 800 to 25 300)**	**2·38 (1·74 to 3·44)**	**12 900 (9860 to 16 800)**	**0·823 (0·623 to 1·08)**	**−30·1% (−57·5 to 3·54)**	**−65·4% (−80·3 to −47·3)**
South Asia		18 500 (13 800 to 25 300)	2·38 (1·74 to 3·44)	12 900 (9860 to 16 800)	0·823 (0·623 to 1·08)	−30·1% (−57·5 to 3·54)	−65·4% (−80·3 to −47·3)
	Bangladesh	1750 (1080 to 2800)	2·53 (1·61 to 4·23)	1110 (706 to 1860)	0·773 (0·493 to 1·25)	−36·7% (−62·9 to 18·3)	−69·4% (−82·0 to −44·5)
	Bhutan	8·40 (4·04 to 20·2)	2·09 (1·07 to 5·40)	5·52 (3·16 to 13·2)	0·848 (0·493 to 1·97)	−34·3% (−61·7 to 24·8)	−59·5% (−76·3 to −27·2)
	India	15 300 (11 500 to 19 300)	2·47 (1·84 to 3·23)	10 000 (6770 to 13 300)	0·806 (0·535 to 1·08)	−34·5% (−63·1 to −0·167)	−67·3% (−82·7 to −48·7)
	Nepal	344 (178 to 886)	2·56 (1·26 to 7·15)	228 (149 to 389)	0·923 (0·607 to 1·54)	−33·6% (−61·8 to 20·0)	−64·0% (−80·1 to −34·2)
	Pakistan	1110 (566 to 2730)	1·52 (0·781 to 3·95)	1570 (1060 to 2230)	0·975 (0·659 to 1·37)	41·7% (−41·9 to 157)	−35·9% (−74·6 to 15·9)
**Southeast Asia, east Asia, and Oceania**		**7280 (5170 to 8980)**	**0·603 (0·424 to 0·762)**	**4180 (3430 to 5100)**	**0·181 (0·149 to 0·221)**	**−42·5% (−54·5 to −21·4)**	**−69·9% (−76·6 to −59·5)**
East Asia		3700 (2680 to 4470)	0·480 (0·344 to 0·586)	1710 (1310 to 2220)	0·0927 (0·0714 to 0·120)	−53·8% (−67·1 to −31·6)	−80·7% (−86·3 to −71·0)
	China	3620 (2610 to 4380)	0·489 (0·350 to 0·600)	1620 (1230 to 2140)	0·0917 (0·0693 to 0·121)	−55·2% (−68·4 to −32·9)	−81·3% (−86·8 to −71·5)
	North Korea	46·9 (26·8 to 68·0)	0·331 (0·191 to 0·490)	57·5 (28·8 to 95·4)	0·198 (0·0990 to 0·334)	22·7% (−30·7 to 114)	−40·3% (−66·7 to 2·68)
	Taiwan (province of China)	28·0 (25·6 to 30·8)	0·204 (0·185 to 0·226)	26·5 (22·1 to 30·9)	0·0638 (0·0537 to 0·0743)	−5·55% (−22·1 to 12·1)	−68·8% (−74·2 to −63·1)
Oceania		14·4 (7·65 to 38·8)	0·366 (0·200 to 0·979)	21·2 (12·9 to 44·1)	0·233 (0·145 to 0·497)	47·7% (−0·684 to 104)	−37·4% (−56·9 to −16·8)
	American Samoa	0·130 (0·0954 to 0·173)	0·479 (0·352 to 0·654)	0·133 (0·0986 to 0·175)	0·311 (0·229 to 0·411)	2·24% (−32·1 to 56·1)	−35·0% (−58·1 to −2·24)
	Cook Islands	0·0193 (0·0132 to 0·0256)	0·134 (0·0917 to 0·178)	0·0126 (0·00677 to 0·0187)	0·0572 (0·0311 to 0·0837)	−34·8% (−63·6 to 16·7)	−57·2% (−75·7 to −23·1)
	Federated States of Micronesia	0·444 (0·228 to 0·648)	0·694 (0·355 to 1·03)	0·302 (0·180 to 0·442)	0·394 (0·243 to 0·563)	−32·0% (−58·6 to 14·0)	−43·2% (−67·2 to −6·39)
	Fiji	2·38 (1·77 to 3·42)	0·514 (0·362 to 0·783)	2·92 (1·99 to 4·00)	0·400 (0·275 to 0·543)	22·8% (−27·2 to 94·5)	−22·2% (−56·3 to 28·3)
	Guam	0·123 (0·0978 to 0·154)	0·158 (0·124 to 0·198)	0·148 (0·115 to 0·188)	0·0827 (0·0650 to 0·105)	20·3% (−10·8 to 64·3)	−47·6% (−61·6 to −27·8)
	Kiribati	0·299 (0·113 to 0·457)	0·608 (0·234 to 0·979)	0·393 (0·186 to 0·607)	0·451 (0·219 to 0·675)	31·5% (−16·2 to 112)	−25·8% (−52·5 to 16·9)
	Marshall Islands	0·170 (0·0888 to 0·254)	0·706 (0·382 to 1·11)	0·172 (0·0934 to 0·256)	0·450 (0·260 to 0·641)	1·08% (−44·4 to 64·0)	−36·3% (−65·0 to 1·73)
	Nauru	0·0383 (0·0141 to 0·0588)	0·673 (0·250 to 1·02)	0·0297 (0·0110 to 0·0458)	0·408 (0·158 to 0·623)	−22·4% (−52·0 to 24·5)	−39·5% (−65·9 to −5·40)
	Niue	0·00798 (0·00542 to 0·0108)	0·346 (0·234 to 0·471)	0·00458 (0·00320 to 0·00617)	0·255 (0·177 to 0·342)	−42·5% (−63·2 to −13·1)	−26·5% (−53·4 to 12·0)
	Northern Mariana Islands	0·0321 (0·0198 to 0·0459)	0·144 (0·0913 to 0·197)	0·0403 (0·0227 to 0·0508)	0·111 (0·0617 to 0·144)	25·4% (−16·7 to 105)	−23·1% (−49·0 to 24·3)
	Palau	0·0401 (0·0219 to 0·0582)	0·359 (0·210 to 0·506)	0·0395 (0·0218 to 0·0553)	0·218 (0·118 to 0·302)	−1·61% (−37·1 to 58·3)	−39·2% (−60·5 to −5·22)
	Papua New Guinea	7·74 (2·94 to 27·2)	0·294 (0·117 to 1·09)	12·9 (6·44 to 33·3)	0·193 (0·0939 to 0·543)	66·8% (−2·60 to 218)	−34·4% (−59·1 to 10·9)
	Samoa	0·369 (0·213 to 0·541)	0·375 (0·219 to 0·558)	0·389 (0·264 to 0·554)	0·258 (0·176 to 0·368)	5·36% (−26·4 to 61·8)	−31·2% (−52·0 to 4·08)
	Solomon Islands	0·724 (0·295 to 1·58)	0·411 (0·183 to 0·943)	1·30 (0·688 to 2·20)	0·285 (0·153 to 0·504)	79·2% (11·9 to 210)	−30·6% (−55·2 to 14·2)
	Tokelau	0·00639 (0·00395 to 0·0106)	0·501 (0·310 to 0·839)	0·00410 (0·00302 to 0·00549)	0·280 (0·207 to 0·373)	−35·8% (−58·6 to 6·14)	−44·1% (−64·4 to −6·59)
	Tonga	0·295 (0·183 to 0·604)	0·510 (0·307 to 1·06)	0·297 (0·193 to 0·435)	0·353 (0·229 to 0·515)	0·730% (−43·8 to 67·9)	−30·7% (−61·7 to 17·9)
	Tuvalu	0·0449 (0·0240 to 0·0653)	0·641 (0·359 to 0·942)	0·0364 (0·0246 to 0·0499)	0·356 (0·246 to 0·480)	−19·0% (−45·8 to 29·8)	−44·5% (−63·4 to −11·8)
	Vanuatu	0·580 (0·228 to 1·36)	0·718 (0·257 to 1·88)	1·13 (0·568 to 2·09)	0·543 (0·258 to 1·07)	94·7% (23·4 to 226)	−24·4% (−51·2 to 25·1)
Southeast Asia		3570 (2400 to 4890)	0·939 (0·656 to 1·40)	2460 (1900 to 3050)	0·380 (0·296 to 0·477)	−31·2% (−45·3 to −0·949)	−59·6% (−70·4 to −46·0)
	Cambodia	176 (86·2 to 256)	2·09 (1·10 to 3·33)	129 (79·1 to 217)	0·901 (0·566 to 1·46)	−26·6% (−53·1 to 42·5)	−57·0% (−73·1 to −25·5)
	Indonesia	1770 (1160 to 2560)	1·27 (0·786 to 1·99)	1220 (855 to 1570)	0·561 (0·392 to 0·717)	−31·0% (−51·3 to −0·499)	−55·7% (−71·7 to −37·7)
	Laos	44·3 (20·1 to 63·7)	1·30 (0·699 to 1·99)	29·5 (17·3 to 52·7)	0·497 (0·295 to 0·893)	−33·4% (−62·3 to 34·6)	−61·8% (−75·8 to −34·3)
	Malaysia	37·0 (26·4 to 58·7)	0·284 (0·199 to 0·461)	42·0 (26·5 to 67·1)	0·147 (0·0941 to 0·238)	13·7% (−17·6 to 56·0)	−48·3% (−62·4 to −29·8)
	Maldives	0·272 (0·116 to 0·411)	0·173 (0·0795 to 0·258)	0·151 (0·109 to 0·206)	0·0409 (0·0295 to 0·0544)	−44·6% (−67·8 to 62·1)	−76·4% (−86·0 to −40·7)
	Mauritius	1·46 (1·28 to 1·66)	0·176 (0·153 to 0·200)	1·10 (0·948 to 1·25)	0·0703 (0·0611 to 0·0801)	−25·0% (−38·7 to −8·97)	−60·0% (−67·3 to −51·2)
	Myanmar	679 (347 to 1010)	1·81 (0·973 to 2·73)	317 (207 to 504)	0·582 (0·387 to 0·914)	−53·3% (−71·5 to −18·8)	−67·8% (−79·4 to −48·2)
	Philippines	351 (250 to 432)	0·641 (0·478 to 0·877)	389 (318 to 469)	0·393 (0·324 to 0·479)	10·5% (−13·6 to 57·6)	−38·7% (−56·5 to −16·8)
	Seychelles	0·579 (0·482 to 0·681)	0·945 (0·782 to 1·11)	0·465 (0·375 to 0·577)	0·431 (0·349 to 0·529)	−19·8% (−35·4 to 4·58)	−54·4% (−63·2 to −40·5)
	Sri Lanka	41·1 (30·1 to 54·0)	0·322 (0·240 to 0·424)	15·4 (10·1 to 22·2)	0·0664 (0·0447 to 0·0959)	−62·6% (−77·7 to −36·6)	−79·4% (−87·6 to −65·4)
	Thailand	179 (123 to 289)	0·373 (0·256 to 0·630)	111 (63·6 to 205)	0·130 (0·0765 to 0·230)	−38·1% (−62·5 to 0·236)	−65·1% (−77·8 to −45·9)
	Timor-Leste	6·97 (3·84 to 14·9)	1·14 (0·616 to 3·16)	6·79 (3·90 to 15·1)	0·638 (0·359 to 1·46)	−2·67% (−40·5 to 95·9)	−43·8% (−63·1 to 0·0823)
	Viet Nam	276 (162 to 466)	0·540 (0·319 to 0·965)	189 (118 to 280)	0·203 (0·129 to 0·303)	−31·7% (−58·1 to 13·1)	−62·3% (−77·7 to −37·4)
**Sub-Saharan Africa**		**2690 (1710 to 4620)**	**0·798 (0·543 to 1·66)**	**3370 (2380 to 5820)**	**0·480 (0·348 to 0·853)**	**25·4% (−2·82 to 98·0)**	**−40·0% (−51·0 to −17·3)**
Central sub-Saharan Africa		259 (122 to 403)	0·735 (0·350 to 1·28)	452 (271 to 826)	0·561 (0·326 to 1·01)	74·6% (11·2 to 233)	−23·6% (−50·7 to 30·8)
	Angola	48·6 (17·4 to 83·2)	0·762 (0·322 to 1·42)	86·2 (46·0 to 140)	0·475 (0·251 to 0·761)	77·2% (9·30 to 259)	−37·7% (−59·4 to 7·66)
	Central African Republic	16·9 (7·11 to 27·2)	0·969 (0·404 to 1·68)	33·2 (14·6 to 56·2)	1·00 (0·477 to 1·69)	96·0% (24·6 to 263)	3·43% (−30·6 to 77·0)
	Congo (Brazzaville)	9·81 (5·36 to 13·9)	0·681 (0·357 to 1·00)	15·8 (10·2 to 23·9)	0·471 (0·289 to 0·685)	61·2% (4·83 to 255)	−30·9% (−53·9 to 41·8)
	Democratic Republic of the Congo	178 (80·1 to 287)	0·725 (0·325 to 1·28)	311 (172 to 611)	0·581 (0·314 to 1·20)	74·5% (0·329 to 242)	−19·8% (−53·2 to 44·2)
	Equatorial Guinea	2·95 (1·27 to 4·82)	1·06 (0·474 to 1·78)	2·51 (1·42 to 4·16)	0·303 (0·175 to 0·501)	−15·0% (−57·9 to 114)	−71·4% (−85·7 to −36·7)
	Gabon	2·63 (1·22 to 4·28)	0·380 (0·166 to 0·640)	3·74 (2·23 to 5·73)	0·305 (0·185 to 0·474)	42·2% (−11·2 to 168)	−19·8% (−49·8 to 48·0)
Eastern sub-Saharan Africa		1040 (513 to 2000)	0·851 (0·448 to 2·12)	1450 (865 to 3450)	0·577 (0·355 to 1·46)	40·0% (−6·49 to 156)	−32·2% (−50·2 to 7·08)
	Burundi	33·6 (11·9 to 61·4)	0·863 (0·296 to 2·06)	45·3 (18·2 to 118)	0·592 (0·212 to 1·76)	34·5% (−15·7 to 177)	−31·3% (−55·2 to 13·5)
	Comoros	2·27 (1·04 to 5·07)	0·748 (0·318 to 1·93)	3·05 (1·69 to 6·05)	0·550 (0·298 to 1·13)	34·0% (−13·6 to 163)	−26·5% (−54·3 to 28·1)
	Djibouti	1·84 (1·07 to 4·78)	0·794 (0·449 to 2·35)	4·32 (2·33 to 9·86)	0·554 (0·305 to 1·27)	135% (50·1 to 293)	−30·3% (−55·4 to 11·4)
	Eritrea	24·5 (10·5 to 44·0)	1·23 (0·581 to 2·94)	34·8 (19·3 to 58·6)	0·881 (0·493 to 1·56)	41·9% (−5·40 to 151)	−28·3% (−51·4 to 6·12)
	Ethiopia	310 (128 to 539)	1·04 (0·483 to 2·20)	310 (165 to 1050)	0·499 (0·247 to 1·80)	0·0367% (−45·5 to 190)	−52·1% (−73·2 to 28·4)
	Kenya	76·1 (48·0 to 243)	0·612 (0·352 to 2·34)	147 (91·7 to 334)	0·522 (0·319 to 1·19)	92·7% (25·6 to 187)	−14·8% (−52·5 to 26·1)
	Madagascar	66·8 (34·1 to 131)	0·845 (0·435 to 2·18)	112 (59·6 to 270)	0·661 (0·356 to 1·73)	67·1% (5·00 to 217)	−21·8% (−47·3 to 23·1)
	Malawi	65·9 (30·5 to 115)	0·941 (0·468 to 2·20)	73·1 (42·7 to 140)	0·643 (0·378 to 1·31)	11·0% (−28·4 to 102)	−31·7% (−52·5 to 5·19)
	Mozambique	104 (48·4 to 223)	1·06 (0·555 to 2·90)	173 (90·0 to 348)	0·971 (0·543 to 1·95)	67·2% (6·66 to 192)	−8·55% (−39·3 to 42·2)
	Rwanda	48·4 (18·2 to 72·3)	0·894 (0·306 to 1·59)	46·2 (21·7 to 136)	0·535 (0·224 to 1·59)	−4·45% (−51·2 to 196)	−40·2% (−67·2 to 49·6)
	Somalia	46·3 (16·7 to 104)	1·09 (0·428 to 3·35)	96·6 (32·6 to 243)	0·952 (0·321 to 2·85)	109% (30·1 to 228)	−12·9% (−43·3 to 27·7)
	South Sudan	32·5 (13·2 to 79·2)	0·830 (0·363 to 2·49)	49·1 (22·0 to 124)	0·800 (0·352 to 2·38)	51·3% (−0·878 to 148)	−3·71% (−35·6 to 52·6)
	Tanzania	132 (68·2 to 242)	0·713 (0·408 to 1·69)	205 (113 to 455)	0·526 (0·287 to 1·19)	55·1% (−6·74 to 210)	−26·3% (−51·7 to 23·6)
	Uganda	47·7 (19·6 to 92·6)	0·426 (0·152 to 1·10)	87·7 (49·8 to 144)	0·339 (0·180 to 0·594)	83·7% (13·3 to 296)	−20·6% (−51·8 to 51·4)
	Zambia	44·8 (26·1 to 65·9)	0·899 (0·632 to 1·50)	63·7 (40·3 to 91·2)	0·573 (0·373 to 0·805)	42·0% (−12·1 to 157)	−36·2% (−60·5 to 2·01)
Southern sub-Saharan Africa		197 (159 to 326)	0·474 (0·374 to 0·835)	297 (243 to 364)	0·421 (0·345 to 0·516)	50·7% (−11·4 to 95·6)	−11·3% (−50·1 to 18·3)
	Botswana	7·83 (4·82 to 15·5)	0·855 (0·533 to 1·83)	6·06 (4·15 to 9·67)	0·312 (0·215 to 0·481)	−22·6% (−56·8 to 29·8)	−63·5% (−80·5 to −39·1)
	Eswatini	4·56 (3·11 to 7·33)	0·830 (0·560 to 1·40)	4·53 (2·74 to 6·96)	0·506 (0·317 to 0·781)	−0·672% (−54·1 to 63·1)	−39·1% (−74·4 to 1·88)
	Lesotho	9·17 (5·56 to 21·5)	0·786 (0·470 to 1·95)	10·7 (6·41 to 16·0)	0·712 (0·428 to 1·09)	16·5% (−64·0 to 104)	−9·43% (−73·9 to 63·3)
	Namibia	8·48 (5·39 to 16·6)	0·872 (0·558 to 1·75)	7·76 (5·09 to 12·0)	0·415 (0·275 to 0·629)	−8·41% (−48·8 to 47·8)	−52·4% (−74·4 to −25·2)
	South Africa	135 (110 to 207)	0·431 (0·349 to 0·708)	205 (161 to 245)	0·391 (0·303 to 0·471)	51·8% (3·87 to 93·5)	−9·26% (−40·3 to 14·8)
	Zimbabwe	32·1 (22·0 to 60·6)	0·511 (0·348 to 0·952)	63·1 (35·6 to 109)	0·561 (0·321 to 0·971)	96·6% (−28·5 to 266)	9·78% (−60·3 to 98·3)
Western sub-Saharan Africa		1190 (746 to 1940)	0·876 (0·556 to 1·70)	1170 (814 to 1540)	0·402 (0·291 to 0·534)	−2·07% (−30·0 to 49·1)	−54·2% (−71·0 to −34·6)
	Benin	35·8 (21·2 to 64·2)	1·04 (0·615 to 2·29)	33·7 (22·1 to 50·5)	0·420 (0·273 to 0·645)	−5·88% (−38·0 to 52·4)	−59·5% (−74·2 to −36·3)
	Burkina Faso	70·1 (38·7 to 143)	0·989 (0·574 to 2·49)	71·8 (44·5 to 118)	0·480 (0·310 to 0·808)	2·43% (−34·9 to 73·6)	−51·5% (−69·4 to −24·1)
	Cabo Verde	0·376 (0·231 to 0·720)	0·137 (0·0854 to 0·278)	0·365 (0·256 to 0·482)	0·0825 (0·0568 to 0·110)	−2·95% (−57·6 to 58·8)	−39·8% (−76·1 to 0·319)
	Cameroon	52·9 (27·9 to 81·7)	0·777 (0·424 to 1·17)	57·2 (30·6 to 87·7)	0·311 (0·172 to 0·488)	8·13% (−35·1 to 80·2)	−59·9% (−77·7 to −35·0)
	Chad	43·5 (25·9 to 109)	1·02 (0·593 to 2·96)	57·6 (38·0 to 91·5)	0·587 (0·387 to 1·05)	32·3% (−22·2 to 111)	−43·0% (−66·8 to −6·49)
	Côte d'Ivoire	54·2 (27·8 to 86·9)	0·779 (0·419 to 1·38)	55·1 (36·9 to 74·6)	0·344 (0·240 to 0·473)	1·71% (−38·3 to 81·2)	−55·9% (−73·3 to −20·7)
	The Gambia	6·66 (3·95 to 13·9)	1·14 (0·636 to 2·53)	7·41 (4·77 to 10·5)	0·550 (0·345 to 0·783)	11·2% (−46·6 to 108)	−51·7% (−80·5 to −5·95)
	Ghana	99·6 (72·9 to 133)	1·13 (0·803 to 1·55)	149 (99·6 to 233)	0·771 (0·500 to 1·16)	49·5% (−4·70 to 158)	−32·1% (−60·6 to 14·0)
	Guinea	44·7 (20·4 to 78·9)	0·865 (0·424 to 1·83)	34·3 (19·7 to 51·2)	0·408 (0·227 to 0·618)	−23·2% (−54·0 to 25·4)	−52·9% (−74·3 to −22·5)
	Guinea-Bissau	10·3 (4·51 to 16·2)	1·55 (0·722 to 2·31)	7·23 (4·24 to 10·7)	0·674 (0·395 to 0·996)	−29·6% (−53·4 to 25·8)	−56·6% (−73·1 to −21·8)
	Liberia	17·8 (9·23 to 27·4)	0·932 (0·488 to 1·64)	11·8 (6·93 to 22·6)	0·393 (0·241 to 0·709)	−33·8% (−60·6 to 39·6)	−57·8% (−74·4 to −16·7)
	Mali	88·2 (42·6 to 151)	1·45 (0·802 to 3·03)	79·7 (50·4 to 131)	0·604 (0·389 to 1·07)	−9·64% (−39·0 to 46·4)	−58·8% (−71·5 to −35·5)
	Mauritania	11·6 (6·07 to 17·3)	0·865 (0·437 to 1·34)	7·46 (4·72 to 12·4)	0·286 (0·172 to 0·500)	−35·7% (−61·7 to 29·3)	−67·0% (−80·7 to −34·0)
	Niger	73·2 (36·7 to 145)	1·27 (0·705 to 3·56)	79·6 (42·8 to 207)	0·578 (0·303 to 1·73)	8·86% (−42·7 to 105)	−54·5% (−71·3 to −27·8)
	Nigeria	467 (278 to 749)	0·719 (0·409 to 1·36)	426 (247 to 626)	0·290 (0·184 to 0·409)	−8·76% (−43·9 to 43·6)	−59·6% (−77·2 to −40·3)
	São Tomé and Príncipe	0·610 (0·346 to 1·10)	0·658 (0·343 to 1·36)	0·270 (0·167 to 0·419)	0·212 (0·131 to 0·317)	−55·7% (−78·3 to −5·51)	−67·7% (−86·7 to −27·8)
	Senegal	63·5 (37·5 to 120)	1·19 (0·687 to 2·63)	40·6 (28·0 to 58·1)	0·413 (0·278 to 0·592)	−36·0% (−64·6 to 26·7)	−65·3% (−83·7 to −27·4)
	Sierra Leone	27·9 (14·4 to 45·7)	0·861 (0·471 to 1·66)	23·2 (15·3 to 33·7)	0·425 (0·282 to 0·634)	−17·0% (−48·6 to 50·7)	−50·7% (−70·1 to −8·67)
	Togo	24·3 (15·8 to 40·1)	1·14 (0·716 to 2·11)	25·1 (16·9 to 37·9)	0·534 (0·352 to 0·821)	3·16% (−41·3 to 72·6)	−53·0% (−76·6 to −19·8)

Data in parentheses are 95% uncertainty intervals (UIs). Count data and rates are presented to three significant figures.

**Figure 2:**
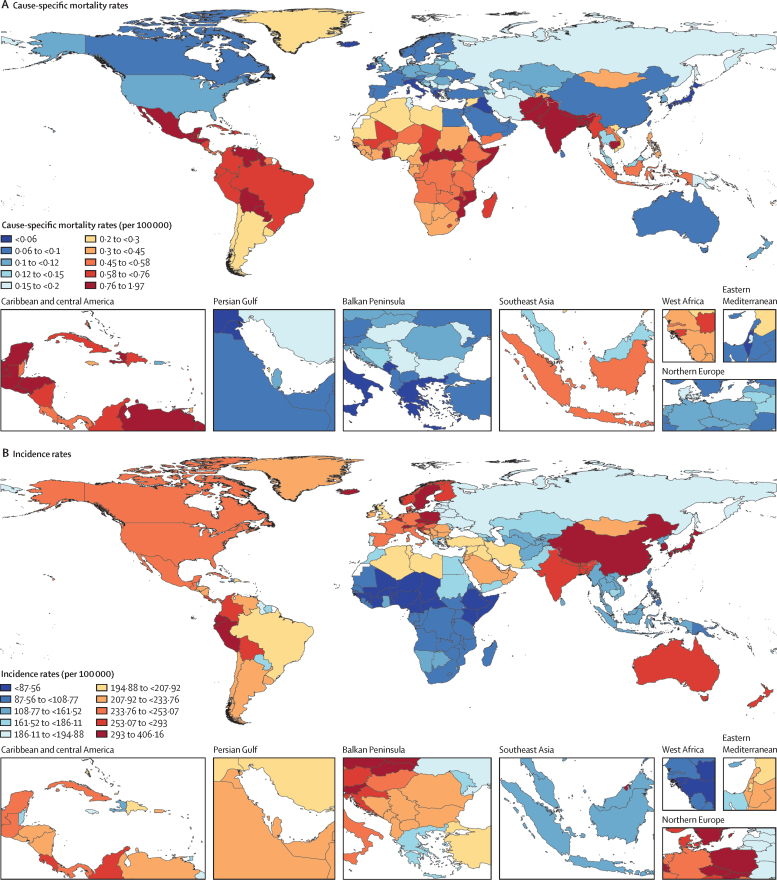
Spatial distribution of the appendicitis burden at all ages and in both sexes, 2021 (A) Age-standardised cause-specific mortality rates of appendicitis in 2021, per 100 000. (B) Age-standardised incidence rates of appendicitis in 2021, per 100 000.

Age-standardised mortality rates ranged from 1·78 (95% UI 1·48–2·10) per 100 000 in Guatemala to 0·0357 (0·0269–0·0463) per 100 000 in Monaco in 2021 (figure 2A). The majority of countries and territories had mean age-standardised mortality rates lower than 1·0 per 100 000. Only nine countries had mean mortality rates higher than 1·0 per 100 000: Guatemala, Haiti, Honduras, Guyana, Bolivia, Mexico, and Central African Republic.

Globally, we estimated 17·0 million (95% UI 13·8–21·6) new cases of appendicitis in 2021 (table 2). The global age-standardised incidence rate was 214 (174–274) per 100 000 in 2021, reflecting a reduction of 2·26% (0·693–3·82) from 1990. The incidence rate of appendicitis was the highest in the second and third decades of life globally and across all seven GBD super-regions (results are accessible through the GBD Compare tool).

**Table 2:** Global, super-regional, regional, and national incidence of appendicitis in 1990 and 2021

		**Incident cases in 1990 (95% UI)**	**Age-standardised incidence rate per 100 000 in 1990 (95% UI)**	**Incident cases in 2021 (95% UI)**	**Age-standardised incidence rate per 100 000 in 2021 (95% UI)**	**Percentage change in incident cases between 1990 and 2021, % (95% UI)**	**Percentage change in age-standardised incidence rates between 1990 and 2021, % (95% UI)**
**Global**		**12 200 000 (9 730 000 to 15 800 000)**	**219 (176 to 283)**	**17 000 000 (13 800 000 to 21 600 000)**	**214 (174 to 274)**	**39·1% (33·3 to 44·7)**	**−2·26% (−3·82 to −0·693)**
**Central Europe, eastern Europe, and central Asia**		**825 000 (660 000 to 1070 000)**	**196 (157 to 257)**	**785 000 (643 000 to 988 000)**	**202 (165 to 261)**	**−4·78% (−8·99 to 0·271)**	**3·17% (0·758 to 6·29)**
Central Asia		112 000 (88 300 to 149 000)	156 (124 to 204)	157 000 (124 000 to 208 000)	162 (128 to 215)	39·9% (31·9 to 47·8)	3·81% (0·0740 to 8·15)
	Armenia	5550 (4280 to 7430)	156 (122 to 208)	5010 (3880 to 6660)	176 (137 to 238)	−9·69% (−15·9 to −1·32)	13·0% (7·87 to 19·0)
	Azerbaijan	11 600 (9000 to 15 400)	148 (117 to 194)	17 200 (13 500 to 23 000)	159 (124 to 214)	47·7% (35·8 to 60·3)	7·74% (0·730 to 15·1)
	Georgia	7710 (5940 to 10 300)	140 (108 to 189)	5430 (4310 to 7070)	168 (132 to 225)	−29·6% (−34·6 to −24·0)	20·0% (13·5 to 27·2)
	Kazakhstan	27 200 (21 300 to 35 000)	159 (126 to 203)	31 200 (24 700 to 41 900)	168 (131 to 226)	14·9% (7·36 to 24·2)	5·32% (−0·599 to 12·7)
	Kyrgyzstan	6810 (5280 to 9070)	149 (117 to 196)	11 400 (8760 to 15 300)	162 (124 to 214)	68·1% (56·0 to 79·1)	8·64% (0·864 to 15·5)
	Mongolia	6490 (5520 to 7790)	279 (240 to 327)	7390 (6040 to 9230)	221 (178 to 275)	13·8% (1·32 to 28·9)	−20·8% (−28·5 to −11·3)
	Tajikistan	8100 (6280 to 10 800)	147 (116 to 194)	16 200 (12 500 to 21 400)	151 (118 to 199)	99·6% (87·8 to 113)	2·94% (−2·86 to 7·72)
	Turkmenistan	5780 (4450 to 7640)	149 (118 to 194)	8440 (6550 to 11 300)	157 (122 to 210)	46·1% (36·9 to 56·3)	5·36% (−0·552 to 11·8)
	Uzbekistan	32 900 (25 200 to 44 500)	151 (119 to 201)	54 500 (42 700 to 73 500)	155 (121 to 209)	65·8% (51·3 to 80·0)	2·58% (−3·83 to 8·87)
Central Europe		286 000 (231 000 to 376 000)	231 (185 to 305)	255 000 (218 000 to 303 000)	263 (221 to 318)	−10·7% (−20·1 to −1·65)	13·8% (3·24 to 23·6)
	Albania	6980 (5330 to 9540)	195 (152 to 265)	5280 (4140 to 6810)	216 (167 to 285)	−24·2% (−30·8 to −16·3)	10·4% (5·72 to 16·6)
	Bosnia and Herzegovina	9610 (7630 to 12 800)	206 (164 to 273)	6160 (4940 to 7850)	214 (166 to 285)	−35·9% (−39·9 to −31·3)	3·54% (−0·804 to 8·05)
	Bulgaria	17 300 (13 800 to 22 700)	210 (165 to 280)	13 000 (10 600 to 16 300)	226 (177 to 299)	−25·0% (−30·0 to −19·4)	7·70% (2·93 to 12·5)
	Croatia	13 900 (11 900 to 15 900)	311 (261 to 359)	9170 (8760 to 9590)	284 (268 to 299)	−34·2% (−41·1 to −23·9)	−8·67% (−19·1 to 6·79)
	Czechia	22 500 (18 100 to 29 300)	221 (174 to 291)	26 400 (21 800 to 30 500)	321 (259 to 381)	17·3% (0·963 to 38·3)	45·1% (24·3 to 74·1)
	Hungary	22 300 (18 000 to 29 200)	220 (174 to 290)	19 500 (15 900 to 24 600)	235 (186 to 309)	−12·7% (−17·8 to −7·18)	6·83% (1·92 to 11·5)
	Montenegro	1370 (1070 to 1820)	215 (168 to 286)	1260 (994 to 1660)	225 (173 to 303)	−7·82% (−13·0 to −2·59)	4·71% (0·00670 to 9·45)
	North Macedonia	4110 (3160 to 5470)	201 (155 to 267)	4260 (3360 to 5590)	217 (167 to 292)	3·70% (−5·85 to 11·5)	8·09% (3·18 to 13·9)
	Poland	105 000 (83 900 to 140 000)	278 (221 to 371)	96 400 (83 400 to 111 000)	295 (251 to 344)	−8·35% (−22·5 to 5·60)	6·16% (−9·27 to 22·6)
	Romania	46 300 (36 800 to 60 800)	199 (158 to 264)	34 700 (27 800 to 44 700)	217 (169 to 288)	−25·1% (−30·6 to −18·9)	8·58% (3·39 to 15·0)
	Serbia	15 900 (12 500 to 21 000)	167 (130 to 223)	16 800 (14 400 to 19 400)	219 (185 to 256)	5·83% (−9·28 to 20·9)	31·3% (11·9 to 51·4)
	Slovakia	11 500 (8870 to 15 500)	218 (168 to 293)	13 600 (11 100 to 16 500)	305 (243 to 373)	17·8% (3·03 to 32·8)	39·7% (23·6 to 55·8)
	Slovenia	4430 (3470 to 5880)	228 (176 to 303)	4970 (4600 to 5290)	315 (287 to 340)	12·2% (−12·4 to 39·9)	38·5% (7·05 to 74·8)
Eastern Europe		427 000 (340 000 to 555 000)	187 (149 to 246)	373 000 (294 000 to 478 000)	192 (154 to 255)	−12·7% (−17·0 to −7·86)	2·68% (−0·0890 to 5·56)
	Belarus	16 500 (13 100 to 21 700)	160 (126 to 210)	16 600 (13 100 to 21 000)	193 (151 to 255)	0·509% (−7·32 to 8·62)	20·7% (14·6 to 26·9)
	Estonia	2530 (2010 to 3290)	167 (131 to 219)	2240 (1770 to 2890)	194 (152 to 257)	−11·7% (−16·8 to −5·86)	16·2% (10·7 to 22·3)
	Latvia	4490 (3600 to 5870)	173 (138 to 231)	3050 (2400 to 3940)	191 (150 to 255)	−32·2% (−36·2 to −27·1)	10·3% (4·99 to 15·8)
	Lithuania	6170 (4930 to 8110)	168 (133 to 225)	4590 (3660 to 5890)	192 (151 to 254)	−25·6% (−30·5 to −19·7)	14·0% (8·43 to 20·8)
	Moldova	7360 (5890 to 9810)	165 (131 to 219)	6280 (4920 to 8110)	186 (147 to 247)	−14·7% (−20·8 to −7·68)	13·0% (7·53 to 18·8)
	Russia	286 000 (227 000 to 371 000)	186 (149 to 245)	265 000 (210 000 to 339 000)	194 (155 to 256)	−7·11% (−11·4 to −2·30)	3·90% (1·16 to 6·46)
	Ukraine	104 000 (82 900 to 134 000)	200 (158 to 265)	74 600 (59 000 to 97 000)	189 (149 to 250)	−28·2% (−33·1 to −22·7)	−5·69% (−10·4 to −0·506)
	High income	2 350 000 (1 870 000 to 3 070 000)	260 (205 to 343)	2 420 000 (2 040 000 to 2 950 000)	254 (210 to 317)	3·12% (−4·41 to 11·4)	−2·36% (−8·46 to 4·50)
Australasia		59 900 (46 200 to 78 700)	290 (223 to 384)	80 400 (63 800 to 104 000)	285 (222 to 378)	34·1% (27·0 to 41·2)	−1·82% (−5·56 to 2·42)
	Australia	49 200 (37 600 to 64 600)	287 (219 to 379)	66 600 (52 300 to 86 800)	285 (221 to 382)	35·4% (27·7 to 42·8)	−0·485% (−4·93 to 4·81)
	New Zealand	10 700 (8390 to 13 900)	307 (241 to 398)	13 700 (11 200 to 16 800)	284 (229 to 354)	27·9% (19·5 to 37·4)	−7·65% (−12·9 to −1·66)
High-income Asia Pacific		675 000 (524 000 to 897 000)	381 (293 to 510)	516 000 (420 000 to 654 000)	364 (286 to 475)	−23·6% (−28·9 to −17·7)	−4·28% (−9·69 to 1·14)
	Brunei	900 (662 to 1230)	299 (226 to 404)	1460 (1110 to 1950)	300 (227 to 400)	62·2% (51·7 to 74·7)	0·200% (−4·33 to 5·60)
	Japan	493 000 (386 000 to 653 000)	404 (311 to 543)	345 000 (284 000 to 433 000)	373 (296 to 480)	−30·0% (−36·1 to −23·2)	−7·79% (−15·8 to 0·479)
	Singapore	11 800 (8830 to 16 100)	336 (255 to 455)	16 600 (13 000 to 21 700)	343 (264 to 462)	39·8% (24·3 to 57·2)	2·04% (−3·03 to 7·67)
	South Korea	169 000 (128 000 to 230 000)	329 (253 to 445)	152 000 (119 000 to 199 000)	348 (264 to 474)	−9·70% (−17·5 to 0·291)	5·78% (0·0786 to 12·2)
High-income North America		697 000 (564 000 to 893 000)	248 (201 to 320)	806 000 (718 000 to 905 000)	234 (207 to 266)	15·7% (1·30 to 32·1)	−5·55% (−16·8 to 7·06)
	Canada	66 700 (53 300 to 87 400)	241 (192 to 317)	81 400 (64 400 to 104 000)	235 (184 to 309)	22·0% (15·7 to 29·0)	−2·30% (−6·10 to 2·25)
	Greenland	125 (94·4 to 169)	203 (156 to 273)	114 (91·2 to 149)	210 (167 to 277)	−8·57% (−14·1 to −0·814)	3·35% (−1·62 to 8·37)
	USA	630 000 (511 000 to 807 000)	249 (202 to 319)	725 000 (651 000 to 807 000)	234 (209 to 262)	15·0% (−0·771 to 32·9)	−5·91% (−18·4 to 8·13)
Southern Latin America		88 600 (69 500 to 119 000)	177 (139 to 237)	149 000 (118 000 to 197 000)	217 (171 to 287)	68·2% (58·9 to 78·3)	22·6% (18·1 to 28·2)
	Argentina	56 200 (43 600 to 75 600)	171 (133 to 230)	97 800 (77 400 to 130 000)	211 (166 to 280)	74·1% (64·5 to 86·3)	23·0% (17·4 to 29·9)
	Chile	26 800 (20 900 to 36 200)	189 (150 to 254)	43 800 (34 300 to 58 000)	230 (180 to 307)	63·4% (51·3 to 75·2)	21·5% (15·3 to 27·2)
	Uruguay	5540 (4360 to 7350)	180 (142 to 240)	7300 (5870 to 9440)	220 (174 to 291)	31·7% (23·2 to 40·6)	22·1% (15·9 to 29·3)
Western Europe		829 000 (654 000 to 1 090 000)	223 (174 to 297)	871 000 (710 000 to 1 100 000)	239 (190 to 310)	5·08% (−0·383 to 11·0)	7·13% (3·25 to 11·2)
	Andorra	129 (98·8 to 171)	223 (171 to 294)	176 (142 to 224)	239 (184 to 318)	37·1% (26·8 to 51·2)	6·90% (1·77 to 12·3)
	Austria	17 800 (13 600 to 23 500)	236 (182 to 318)	20 400 (16 600 to 25 400)	277 (217 to 351)	14·8% (4·81 to 27·0)	17·0% (7·01 to 28·7)
	Belgium	25 900 (20 100 to 34 300)	275 (211 to 369)	35 000 (31 200 to 38 900)	369 (326 to 412)	35·3% (11·4 to 64·9)	34·0% (10·5 to 65·7)
	Cyprus	1090 (833 to 1490)	138 (105 to 190)	1770 (1430 to 2260)	152 (122 to 197)	62·0% (48·5 to 79·0)	10·0% (2·18 to 22·5)
	Denmark	12 400 (9750 to 16 300)	248 (193 to 333)	13 900 (11 100 to 18 000)	265 (203 to 356)	12·4% (7·40 to 18·2)	6·91% (1·60 to 11·2)
	Finland	14 500 (11 900 to 17 600)	308 (249 to 377)	14 100 (11 800 to 16 600)	293 (241 to 351)	−2·87% (−7·50 to 2·89)	−5·01% (−8·95 to −0·139)
	France	123 000 (95 800 to 163 000)	220 (169 to 298)	134 000 (106 000 to 176 000)	235 (179 to 319)	8·85% (3·58 to 14·3)	7·10% (1·74 to 12·1)
	Germany	168 000 (132 000 to 218 000)	222 (171 to 296)	165 000 (131 000 to 213 000)	236 (180 to 316)	−1·69% (−6·84 to 2·62)	5·95% (1·66 to 11·2)
	Greece	16 000 (12 300 to 21 200)	160 (121 to 216)	13 600 (10 700 to 17 500)	172 (131 to 229)	−15·0% (−20·7 to −8·43)	7·56% (1·30 to 14·8)
	Iceland	553 (416 to 748)	213 (161 to 288)	939 (738 to 1180)	297 (229 to 377)	69·8% (49·1 to 104)	39·6% (23·5 to 66·2)
	Ireland	7910 (5980 to 10 700)	214 (163 to 290)	10 200 (7910 to 13 400)	231 (176 to 309)	29·1% (20·8 to 38·8)	7·74% (3·24 to 12·7)
	Israel	10 600 (8010 to 14 700)	209 (158 to 288)	20 500 (15 400 to 27 700)	224 (168 to 305)	92·5% (83·1 to 104)	7·52% (3·35 to 12·8)
	Italy	133 000 (103 000 to 175 000)	235 (182 to 317)	104 000 (86 600 to 123 000)	244 (199 to 297)	−21·9% (−32·8 to −9·43)	3·66% (−11·8 to 20·8)
	Luxembourg	872 (687 to 1150)	240 (186 to 320)	1760 (1560 to 1940)	328 (286 to 365)	102% (62·8 to 150)	36·5% (8·97 to 72·0)
	Malta	645 (495 to 852)	179 (136 to 238)	806 (715 to 871)	237 (208 to 259)	25·0% (−2·90 to 61·6)	32·8% (2·70 to 72·1)
	Monaco	54·7 (43·5 to 71·0)	217 (165 to 293)	66·6 (52·5 to 85·1)	229 (175 to 310)	21·7% (16·0 to 27·6)	5·54% (1·07 to 10·2)
	Netherlands	34 100 (26 400 to 45 100)	229 (177 to 306)	35 900 (28 400 to 46 500)	240 (184 to 323)	5·26% (−0·819 to 12·2)	4·91% (0·679 to 10·3)
	Norway	13 400 (10 400 to 17 800)	321 (250 to 433)	14 800 (12 000 to 18 600)	292 (233 to 378)	10·5% (0·723 to 20·1)	−9·12% (−15·8 to −2·10)
	Portugal	14 200 (10 900 to 18 900)	141 (108 to 188)	15 900 (13 000 to 19 200)	194 (152 to 249)	11·8% (−1·62 to 28·1)	37·9% (22·6 to 63·9)
	San Marino	53·0 (41·0 to 70·1)	222 (171 to 295)	64·6 (51·2 to 83·0)	235 (180 to 317)	21·9% (14·2 to 31·1)	6·01% (1·61 to 11·1)
	Spain	85 200 (65 900 to 114 000)	218 (168 to 292)	90 300 (71 600 to 116 000)	239 (184 to 324)	5·90% (−3·67 to 16·0)	9·83% (4·02 to 14·5)
	Sweden	25 200 (19 800 to 32 900)	316 (247 to 421)	29 800 (23 900 to 38 600)	318 (252 to 421)	18·3% (12·2 to 24·1)	0·693% (−3·56 to 5·26)
	Switzerland	21 400 (18 300 to 24 400)	338 (288 to 389)	26 900 (22 900 to 30 600)	363 (307 to 419)	25·7% (19·9 to 31·2)	7·62% (2·86 to 12·2)
	UK	103 000 (82 300 to 133 000)	191 (150 to 252)	121 000 (99 400 to 151 000)	200 (162 to 253)	17·8% (11·4 to 25·0)	4·70% (−0·205 to 9·86)
**Latin America and Caribbean**		**935 000 (777 000 to 1150 000)**	**223 (186 to 274)**	**1 410 000 (1 160 000 to 1 760 000)**	**232 (191 to 292)**	**50·5**%**(43·5 to 57·0)**	**4·01**%**(1·33 to 7·62)**
Andean Latin America		165 000 (143 000 to 192 000)	401 (351 to 461)	240 000 (189 000 to 302 000)	345 (273 to 433)	45·4% (25·6 to 67·9)	−14·0% (−24·8 to −0·941)
	Bolivia	17 500 (15 100 to 20 300)	269 (234 to 308)	33 500 (26 600 to 41 800)	269 (216 to 332)	91·8% (66·6 to 119)	0·129% (−11·9 to 12·9)
	Ecuador	34 100 (29 200 to 40 000)	317 (274 to 369)	64 400 (51 500 to 82 000)	341 (274 to 434)	89·0% (66·9 to 112)	7·47% (−3·71 to 19·5)
	Peru	113 000 (98 400 to 131 000)	478 (420 to 550)	142 000 (111 000 to 179 000)	372 (294 to 470)	25·2% (6·00 to 47·8)	−22·1% (−33·3 to −08·29)
Caribbean		61 500 (48 700 to 79 600)	167 (134 to 214)	91 900 (74 400 to 116 000)	191 (154 to 242)	49·5% (42·4 to 56·3)	14·2% (11·4 to 17·1)
	Antigua and Barbuda	107 (83·1 to 142)	169 (134 to 221)	194 (154 to 253)	211 (166 to 276)	81·5% (68·5 to 94·6)	24·2% (17·7 to 30·9)
	The Bahamas	451 (350 to 595)	159 (127 to 206)	802 (628 to 1040)	196 (154 to 255)	77·9% (67·5 to 88·9)	23·0% (16·7 to 29·7)
	Barbados	438 (341 to 579)	164 (130 to 215)	575 (452 to 734)	204 (160 to 268)	31·3% (22·7 to 41·6)	24·8% (18·0 to 30·8)
	Belize	267 (204 to 357)	142 (111 to 186)	869 (677 to 1120)	184 (146 to 235)	226% (204 to 251)	29·9% (22·8 to 37·3)
	Bermuda	110 (86·5 to 144)	171 (136 to 226)	131 (103 to 166)	230 (180 to 300)	18·8% (9·28 to 30·4)	34·5% (28·4 to 43·6)
	Cuba	23 000 (18 200 to 29 900)	195 (157 to 251)	26 400 (21 500 to 33 100)	239 (194 to 307)	14·4% (4·92 to 24·5)	22·6% (16·7 to 28·9)
	Dominica	109 (84·9 to 143)	147 (117 to 192)	118 (92·3 to 153)	173 (136 to 225)	8·37% (0·949 to 15·5)	17·4% (10·5 to 23·8)
	Dominican Republic	12 700 (9960 to 16 500)	163 (130 to 208)	23 500 (18 700 to 29 900)	202 (161 to 257)	84·8% (73·7 to 96·9)	24·4% (19·1 to 30·1)
	Grenada	122 (95·0 to 159)	142 (114 to 184)	195 (153 to 255)	184 (145 to 243)	60·4% (49·7 to 70·9)	29·2% (22·9 to 36·4)
	Guyana	1340 (1090 to 1660)	165 (135 to 199)	1540 (1260 to 1890)	194 (159 to 235)	15·0% (8·61 to 21·7)	17·9% (12·5 to 24·0)
	Haiti	7850 (6480 to 9550)	125 (105 to 150)	18 000 (14 600 to 22 100)	132 (108 to 159)	130% (116 to 143)	5·65% (0·598 to 10·8)
	Jamaica	3930 (3010 to 5210)	160 (126 to 209)	5800 (4500 to 7700)	193 (152 to 257)	47·6% (37·2 to 58·3)	20·1% (13·3 to 27·1)
	Puerto Rico	5840 (4580 to 7760)	158 (124 to 209)	6330 (4930 to 8260)	212 (163 to 284)	8·31% (0·795 to 16·1)	34·3% (26·8 to 41·8)
	Saint Kitts and Nevis	67·7 (52·9 to 86·8)	162 (132 to 203)	119 (94·3 to 157)	196 (156 to 261)	76·5% (60·9 to 94·5)	21·4% (14·0 to 30·9)
	Saint Lucia	217 (169 to 285)	156 (125 to 202)	357 (285 to 452)	199 (158 to 251)	64·5% (49·8 to 79·9)	27·2% (21·5 to 32·7)
	Saint Vincent and the Grenadines	162 (123 to 216)	141 (110 to 184)	203 (161 to 266)	180 (143 to 238)	24·9% (14·7 to 35·8)	27·7% (20·9 to 34·3)
	Suriname	578 (448 to 753)	141 (112 to 181)	985 (775 to 1280)	171 (133 to 223)	70·3% (58·5 to 81·0)	21·1% (15·0 to 27·2)
	Trinidad and Tobago	1850 (1440 to 2440)	147 (117 to 192)	2520 (1970 to 3310)	185 (144 to 245)	36·2% (25·7 to 46·3)	25·4% (19·1 to 31·4)
	Virgin Islands	213 (170 to 271)	197 (158 to 250)	172 (140 to 214)	227 (180 to 295)	−19·1% (−24·5 to −13·2)	15·0% (8·98 to 21·2)
Central Latin America		374 000 (305 000 to 476 000)	216 (179 to 272)	636 000 (524 000 to 801 000)	245 (202 to 308)	70·1% (60·7 to 78·9)	13·3% (10·9 to 16·0)
	Colombia	63 300 (50 000 to 84 600)	184 (148 to 243)	128 000 (103 000 to 163 000)	253 (205 to 326)	101% (86·0 to 119)	37·4% (30·7 to 45·6)
	Costa Rica	6840 (5470 to 9060)	218 (177 to 286)	12 400 (10 100 to 16 300)	257 (208 to 335)	81·2% (67·3 to 96·5)	17·7% (12·6 to 24·5)
	El Salvador	13 700 (11 100 to 17 000)	245 (204 to 299)	14 600 (11 700 to 18 900)	221 (178 to 285)	6·54% (−4·76 to 19·5)	−9·84% (−18·1 to −0·174)
	Guatemala	30 200 (26 500 to 34 900)	358 (317 to 407)	39 900 (32 700 to 48 800)	234 (192 to 286)	32·2% (15·9 to 52·5)	−34·6% (−41·6 to −25·4)
	Honduras	12 400 (10 400 to 14 900)	264 (226 to 310)	24 300 (20 000 to 29 900)	232 (194 to 281)	96·1% (77·8 to 115)	−12·4% (−19·2 to −5·42)
	Mexico	187 000 (150 000 to 239 000)	207 (169 to 262)	332 000 (276 000 to 411 000)	250 (207 to 309)	77·5% (64·8 to 91·0)	20·8% (17·1 to 25·2)
	Nicaragua	7320 (5770 to 9500)	189 (153 to 242)	15 500 (12 500 to 20 200)	221 (179 to 286)	112% (97·9 to 127)	16·6% (11·4 to 22·4)
	Panama	5350 (4310 to 7000)	210 (172 to 271)	10 300 (8340 to 13 400)	238 (192 to 309)	92·8% (82·2 to 104)	13·5% (8·47 to 19·9)
	Venezuela	48 300 (39 600 to 62 200)	248 (208 to 314)	60 300 (48 900 to 77 700)	232 (188 to 302)	25·0% (16·8 to 33·4)	−6·61% (−12·9 to 0·497)
	Tropical Latin America	335 000 (274 000 to 409 000)	201 (166 to 244)	439 000 (363 000 to 539 000)	196 (161 to 239)	31·0% (25·7 to 38·2)	−2·84% (−6·44 to 0·733)
	Brazil	326 000 (267 000 to 398 000)	201 (166 to 243)	425 000 (352 000 to 520 000)	196 (162 to 239)	30·2% (24·7 to 37·3)	−2·42% (−6·11 to 1·21)
	Paraguay	8750 (6960 to 11 200)	212 (170 to 271)	14 100 (11 100 to 18 400)	184 (146 to 239)	60·7% (50·2 to 70·7)	−13·2% (−17·6 to −8·43)
**North Africa and Middle East**		**571 000 (431 000 to 783 000)**	**159 (123 to 215)**	**1 250 000 (974 000 to 1 700 000)**	**192 (149 to 259)**	**119% (108 to 134)**	**20·6% (18·1 to 23·6)**
North Africa and Middle East		571 000 (431 000 to 783 000)	159 (123 to 215)	1250 000 (974 000 to 1 700 000)	192 (149 to 259)	119% (108 to 134)	20·6% (18·1 to 23·6)
	Afghanistan	13 100 (10 500 to 16 800)	142 (115 to 179)	48 500 (37 200 to 63 200)	150 (119 to 190)	270% (237 to 300)	5·46% (−0·223 to 11·1)
	Algeria	45 000 (34 100 to 61 500)	166 (129 to 225)	87 600 (68 700 to 119 000)	199 (154 to 270)	94·6% (75·7 to 111)	19·5% (13·2 to 27·2)
	Bahrain	1010 (744 to 1380)	173 (132 to 230)	3680 (2830 to 5020)	218 (169 to 297)	265% (239 to 294)	26·3% (19·7 to 33·6)
	Egypt	86 500 (64 500 to 119 000)	148 (111 to 202)	202 000 (156 000 to 280 000)	184 (143 to 253)	134% (120 to 154)	24·9% (18·5 to 34·1)
	Iran	111 000 (84 800 to 151 000)	190 (149 to 255)	176 000 (137 000 to 236 000)	200 (156 to 270)	58·7% (43·2 to 75·0)	5·09% (2·37 to 8·10)
	Iraq	30 400 (22 600 to 42 400)	154 (117 to 212)	91 100 (69 400 to 124 000)	201 (155 to 270)	199% (182 to 220)	29·9% (24·0 to 36·1)
	Jordan	6960 (5170 to 9670)	168 (128 to 233)	31 900 (24 000 to 43 800)	231 (177 to 310)	358% (314 to 429)	37·0% (26·3 to 58·2)
	Kuwait	3640 (2710 to 4980)	184 (141 to 246)	10 900 (8330 to 14 800)	219 (168 to 297)	200% (177 to 231)	19·1% (12·9 to 25·9)
	Lebanon	4960 (3750 to 6880)	161 (123 to 221)	12 100 (9270 to 16 600)	211 (162 to 289)	144% (125 to 168)	31·4% (25·1 to 38·5)
	Libya	7580 (5710 to 10 300)	167 (129 to 223)	15 000 (11 600 to 20 200)	199 (156 to 267)	97·5% (79·7 to 118)	19·2% (13·7 to 24·7)
	Morocco	39 900 (29 700 to 54 400)	146 (112 to 198)	68 000 (53 000 to 92 000)	178 (139 to 241)	70·6% (59·5 to 82·1)	21·9% (15·9 to 28·2)
	Oman	3670 (2740 to 5060)	173 (131 to 234)	11 400 (8620 to 15 800)	216 (165 to 292)	211% (183 to 245)	24·7% (18·1 to 33·2)
	Palestine	3610 (2700 to 5010)	172 (132 to 234)	11 600 (8780 to 16 000)	206 (161 to 279)	221% (200 to 244)	19·9% (13·7 to 26·7)
	Qatar	928 (687 to 1290)	174 (132 to 237)	7930 (5870 to 11 100)	224 (175 to 300)	754% (685 to 834)	28·3% (22·3 to 36·2)
	Saudi Arabia	27 900 (20 800 to 38 600)	160 (122 to 219)	89 900 (68 600 to 123 000)	208 (161 to 283)	222% (188 to 257)	30·4% (23·8 to 37·3)
	Sudan	29 400 (22 300 to 40 100)	142 (110 to 191)	84 000 (63 700 to 114 000)	175 (137 to 236)	185% (170 to 203)	23·7% (17·2 to 29·8)
	Syria	22 500 (17 100 to 30 600)	167 (129 to 225)	29 500 (22 600 to 39 400)	200 (156 to 270)	31·1% (21·0 to 42·3)	20·1% (12·9 to 25·8)
	Tunisia	14 900 (11 100 to 20 500)	164 (126 to 224)	23 300 (18 000 to 31 400)	202 (155 to 276)	57·0% (43·7 to 74·2)	23·3% (17·3 to 30·0)
	Türkiye	95 500 (70 900 to 133 000)	151 (115 to 208)	167 000 (130 000 to 222 000)	199 (154 to 266)	74·9% (61·9 to 91·4)	31·7% (25·2 to 39·0)
	United Arab Emirates	3680 (2740 to 5120)	170 (129 to 230)	20 900 (15 700 to 29 100)	211 (166 to 285)	468% (388 to 578)	24·4% (17·2 to 30·8)
	Yemen	19 100 (14 500 to 25 600)	147 (114 to 193)	60 500 (45 900 to 82 500)	168 (130 to 226)	218% (199 to 235)	14·4% (9·85 to 19·2)
**South Asia**		**3 100 000 (2 550 000 to 3 800 000)**	**282 (233 to 343)**	**4 940 000 (4 040 000 to 6 200 000)**	**245 (202 to 306)**	**59·1**%**(53·1 to 65·7)**	**−12·9**%**(−15·4 to −9·86)**
South Asia		3 100 000 (2 550 000 to 3 800 000)	282 (233 to 343)	4 940 000 (4 040 000 to 6 200 000)	245 (202 to 306)	59·1% (53·1 to 65·7)	−12·9% (−15·4 to −9·86)
	Bangladesh	261 000 (219 000 to 310 000)	244 (207 to 289)	495 000 (390 000 to 641 000)	280 (222 to 361)	89·5% (70·6 to 111)	14·5% (3·66 to 26·8)
	Bhutan	1390 (1150 to 1700)	209 (174 to 253)	2260 (1780 to 2910)	261 (209 to 335)	61·9% (45·9 to 78·8)	25·0% (14·2 to 35·2)
	India	2 580 000 (2 110 000 to 3 170 000)	296 (244 to 361)	3 940 000 (3 230 000 to 4 940 000)	254 (209 to 317)	53·1% (47·9 to 59·1)	−14·0% (−16·3 to −11·3)
	Nepal	51 000 (43 800 to 59 000)	272 (235 to 312)	85 800 (68 600 to 109 000)	254 (205 to 319)	68·2% (49·4 to 89·0)	−6·71% (−16·3 to 4·63)
	Pakistan	215 000 (174 000 to 264 000)	206 (169 to 251)	411 000 (340 000 to 502 000)	164 (136 to 199)	91·2% (83·0 to 101)	−20·1% (−23·3 to −17·3)
**Southeast Asia, east Asia, and Oceania**		**4 030 000 (3 100 000 to 5 410 000)**	**216 (171 to 286)**	**5 150 000 (4 130 000 to 6 630 000)**	**245 (196 to 322)**	**27·8% (16·5 to 39·7)**	**13·4% (9·00 to 17·9)**
East Asia		3 320 000 (2 550 000 to 4 480 000)	244 (192 to 325)	4 120 000 (3 290 000 to 5 300 000)	306 (243 to 405)	23·9% (11·7 to 37·1)	25·4% (19·7 to 31·4)
	China	3 280 000 (2 510 000 to 4 420 000)	249 (196 to 332)	4 040 000 (3 230 000 to 5 200 000)	312 (247 to 412)	23·4% (11·2 to 36·5)	25·3% (19·6 to 31·4)
	North Korea	26 500 (20 700 to 35 500)	124 (97·4 to 166)	42 400 (33 400 to 55 600)	160 (126 to 215)	60·1% (51·0 to 70·8)	29·4% (23·6 to 35·6)
	Taiwan (province of China)	17 800 (13 500 to 24 800)	80·5 (62·3 to 110)	30 700 (25 200 to 37 300)	131 (108 to 166)	72·3% (45·9 to 107)	63·1% (46·5 to 83·7)
Oceania		6300 (4830 to 8440)	94·0 (73·9 to 124)	14 300 (10 900 to 19 200)	99·2 (76·9 to 132)	132% (122 to 142)	5·58% (2·11 to 9·26)
	American Samoa	50·1 (38·2 to 68·1)	101 (78·2 to 135)	59·1 (46·8 to 77·0)	122 (94·9 to 158)	18·0% (9·90 to 28·4)	20·6% (14·2 to 27·7)
	Cook Islands	19·2 (14·6 to 25·9)	98·5 (75·8 to 131)	19·5 (15·4 to 25·6)	117 (91·0 to 157)	1·61% (−6·20 to 9·45)	18·9% (12·6 to 26·2)
	Federated States of Micronesia	108 (83·4 to 139)	107 (85·0 to 134)	129 (101 to 166)	119 (94·6 to 153)	19·4% (11·7 to 27·5)	11·3% (5·68 to 17·5)
	Fiji	810 (617 to 1100)	99·8 (78·2 to 133)	1070 (837 to 1400)	113 (88·6 to 148)	31·8% (22·5 to 40·5)	13·4% (7·10 to 19·8)
	Guam	147 (110 to 201)	96·1 (74·3 to 130)	170 (133 to 225)	111 (86·1 to 150)	15·6% (8·48 to 25·9)	15·6% (10·4 to 21·5)
	Kiribati	78·6 (60·9 to 101)	105 (83·5 to 133)	141 (112 to 177)	111 (89·2 to 138)	78·8% (69·0 to 89·3)	5·45% (−0·177 to 11·2)
	Marshall Islands	46·6 (36·3 to 61·4)	108 (86·2 to 139)	68·6 (53·5 to 89·5)	113 (89·0 to 146)	47·0% (38·0 to 57·3)	4·91% (−0·0316 to 9·83)
	Nauru	10·8 (8·46 to 14·1)	107 (84·6 to 137)	13·9 (10·8 to 17·8)	120 (95·6 to 151)	28·2% (22·2 to 35·1)	12·4% (7·27 to 18·2)
	Niue	2·24 (1·73 to 3·01)	104 (80·0 to 139)	1·84 (1·46 to 2·44)	116 (90·2 to 155)	−17·7% (−22·1 to −11·4)	11·6% (6·41 to 17·4)
	Northern Mariana Islands	57·3 (43·4 to 78·0)	105 (81·9 to 141)	56·0 (44·8 to 73·3)	118 (92·5 to 156)	−2·25% (−10·2 to 7·75)	12·7% (7·41 to 18·4)
	Palau	18·9 (14·5 to 25·0)	112 (87·4 to 147)	23·4 (18·7 to 29·6)	131 (104 to 169)	23·7% (11·7 to 37·3)	17·6% (10·5 to 24·7)
	Papua New Guinea	3790 (2880 to 5060)	90·2 (70·4 to 119)	10 400 (7820 to 14 100)	95·3 (73·2 to 128)	175% (161 to 190)	5·59% (0·709 to 10·6)
	Samoa	165 (125 to 224)	99·1 (77·3 to 131)	237 (185 to 318)	114 (89·3 to 151)	43·6% (34·8 to 53·1)	15·2% (10·0 to 20·7)
	Solomon Islands	316 (245 to 418)	98·0 (78·2 to 126)	758 (592 to 963)	109 (86·2 to 136)	140% (125 to 154)	10·7% (5·08 to 17·1)
	Tokelau	1·45 (1·12 to 1·95)	96·4 (74·9 to 128)	1·52 (1·19 to 2·00)	112 (87·6 to 148)	4·20% (−2·31 to 10·8)	16·4% (10·5 to 22·1)
	Tonga	98·1 (75·1 to 133)	105 (81·8 to 136)	119 (91·7 to 158)	117 (91·7 to 154)	21·6% (15·8 to 28·2)	12·1% (7·23 to 16·8)
	Tuvalu	8·95 (6·89 to 11·6)	96·4 (75·0 to 125)	14·8 (11·5 to 19·5)	117 (91·8 to 152)	65·8% (57·3 to 75·3)	21·6% (15·0 to 28·8)
	Vanuatu	165 (130 to 214)	114 (91·2 to 145)	366 (288 to 466)	116 (92·0 to 145)	121% (110 to 134)	1·43% (−3·21 to 6·36)
Southeast Asia		705 000 (555 000 to 927 000)	141 (112 to 183)	1 020 000 (822 000 to 1 340 000)	141 (114 to 185)	45·2% (38·4 to 52·0)	0·329% (−1·89 to 2·83)
	Cambodia	20 200 (17 100 to 23 900)	194 (166 to 227)	33 500 (27 200 to 42 000)	184 (151 to 228)	65·5% (49·7 to 81·0)	−5·11% (−12·7 to 2·28)
	Indonesia	345 000 (273 000 to 450 000)	173 (138 to 223)	463 000 (371 000 to 595 000)	158 (127 to 203)	34·1% (27·9 to 40·5)	−8·60% (−12·1 to −4·77)
	Laos	4120 (3250 to 5370)	101 (81·4 to 130)	9120 (7140 to 11 900)	114 (90·5 to 148)	121% (106 to 136)	12·8% (5·91 to 19·3)
	Malaysia	20 600 (15 600 to 28 100)	110 (85·5 to 146)	44 800 (35 000 to 59 700)	131 (103 to 174)	117% (102 to 133)	19·3% (13·2 to 26·9)
	Maldives	229 (174 to 311)	104 (80·0 to 137)	737 (553 to 1000)	126 (97·3 to 173)	221% (177 to 273)	21·6% (16·2 to 28·4)
	Mauritius	1350 (1010 to 1860)	111 (84·9 to 151)	1560 (1200 to 2080)	125 (97·0 to 172)	15·3% (6·34 to 26·7)	13·0% (7·32 to 18·8)
	Myanmar	55 800 (44 700 to 71 000)	126 (102 to 159)	82 700 (66 500 to 107 000)	140 (113 to 181)	48·3% (38·9 to 58·4)	11·0% (4·53 to 17·7)
	Philippines	64 900 (50 300 to 87 000)	101 (80·7 to 132)	129 000 (102 000 to 171 000)	106 (84·3 to 140)	98·1% (93·0 to 103)	5·05% (2·62 to 7·53)
	Seychelles	100 (78·7 to 131)	132 (105 to 171)	164 (134 to 212)	154 (123 to 202)	64·1% (50·1 to 77·5)	16·6% (11·4 to 21·8)
	Sri Lanka	20 500 (15 700 to 28 200)	110 (85·6 to 149)	28 400 (22 100 to 38 400)	129 (99·9 to 176)	38·8% (29·3 to 47·8)	17·4% (11·4 to 22·9)
	Thailand	84 800 (64 800 to 113 000)	132 (102 to 175)	89 000 (69 400 to 115 000)	143 (114 to 191)	4·89% (−5·38 to 16·3)	8·52% (3·03 to 14·7)
	Timor-Leste	796 (618 to 1050)	99·8 (79·4 to 129)	1660 (1280 to 2200)	113 (89·9 to 147)	109% (97·0 to 122)	13·4% (8·25 to 19·4)
	Viet Nam	85 400 (65 100 to 113 000)	120 (93·3 to 156)	139 000 (111 000 to 185 000)	138 (110 to 184)	63·2% (47·7 to 80·5)	15·4% (9·42 to 23·2)
**Sub-Saharan Africa**		**376 000 (290 000 to 503 000)**	**78·7 (62·4 to 103)**	**1 000 000 (774 000 to 1 350 000)**	**87·1 (68·5 to 115)**	**167% (161 to 172)**	**10·7% (8·82 to 12·7)**
Central sub-Saharan Africa		38 200 (29 600 to 50 600)	72·9 (57·9 to 94·3)	127 000 (98 500 to 169 000)	93·0 (72·9 to 121)	233% (221 to 248)	27·5% (23·3 to 32·4)
	Angola	7380 (5750 to 9540)	75·1 (59·6 to 95·4)	29 300 (22 400 to 38 700)	92·6 (72·7 to 121)	297% (276 to 317)	23·3% (17·4 to 29·7)
	Central African Republic	2000 (1590 to 2540)	77·7 (63·1 to 96·2)	4700 (3730 to 5910)	86·5 (69·9 to 106)	135% (120 to 148)	11·4% (5·37 to 17·3)
	Congo (Brazzaville)	1740 (1340 to 2280)	74·1 (58·7 to 94·7)	5370 (4170 to 7180)	95·1 (74·5 to 125)	209% (190 to 233)	28·2% (21·4 to 36·1)
	Democratic Republic of the Congo	26 100 (20 300 to 34 900)	71·9 (56·8 to 93·8)	84 500 (65 000 to 113 000)	93·1 (72·5 to 121)	224% (206 to 242)	29·6% (23·3 to 0·361)
	Equatorial Guinea	282 (223 to 372)	72·6 (57·9 to 94·3)	1620 (1210 to 2150)	97·5 (75·8 to 128)	472% (419 to 519)	34·3% (24·4 to 43·7)
	Gabon	699 (526 to 945)	72·7 (57·0 to 95·8)	1830 (1420 to 2440)	96·8 (75·7 to 127)	162% (149 to 179)	33·2% (27·3 to 39·6)
Eastern sub-Saharan Africa		139 000 (108 000 to 184 000)	76·1 (60·6 to 99·1)	383 000 (297 000 to 509 000)	87·5 (69·2 to 114)	175% (167 to 184)	15·0% (12·5 to 18·0)
	Burundi	4140 (3220 to 5370)	78·2 (62·0 to 99·8)	12 300 (9500 to 15 800)	93·2 (73·3 to 117)	196% (181 to 213)	19·2% (13·8 to 25·7)
	Comoros	386 (294 to 518)	87·8 (69·6 to 115)	767 (590 to 1030)	97·9 (76·4 to 130)	99·0% (85·8 to 113)	11·5% (6·06 to 17·3)
	Djibouti	363 (271 to 493)	83·4 (64·7 to 112)	1340 (1030 to 1810)	98·7 (76·8 to 131)	269% (241 to 300)	18·2% (11·6 to 24·2)
	Eritrea	2660 (2040 to 3420)	80·8 (63·2 to 102)	6530 (5090 to 8490)	94·8 (75·1 to 121)	146% (132 to 161)	17·4% (11·9 to 24·0)
	Ethiopia	29 600 (23 500 to 38 400)	62·4 (50·3 to 79·2)	78 400 (59 300 to 106 000)	68·8 (53·3 to 90·8)	165% (145 to 187)	10·3% (3·32 to 18·1)
	Kenya	17 200 (13 100 to 23 400)	79·0 (62·1 to 104)	43 700 (34 000 to 58 700)	82·6 (65·1 to 109)	154% (145 to 164)	4·53% (2·28 to 7·31)
	Madagascar	9670 (7390 to 12 900)	81·7 (64·0 to 108)	27 600 (21 100 to 36 100)	93·0 (73·0 to 120)	185% (169 to 207)	13·9% (8·79 to 20·9)
	Malawi	7920 (6100 to 10 400)	82·4 (65·0 to 105)	19 900 (15 500 to 26 800)	97·6 (77·0 to 126)	151% (139 to 168)	18·4% (12·9 to 25·3)
	Mozambique	10 400 (8130 to 13 500)	82·0 (65·1 to 105)	31 700 (24 900 to 40 600)	104 (83·8 to 129)	206% (188 to 227)	26·7% (19·6 to 35·1)
	Rwanda	6220 (4850 to 7830)	89·0 (71·2 to 111)	13 400 (10 400 to 17 800)	95·6 (75·6 to 124)	116% (98·5 to 136)	7·42% (0·130 to 16·1)
	Somalia	6040 (4750 to 7910)	81·3 (64·8 to 103)	18 100 (14 200 to 23 300)	86·8 (69·0 to 108)	199% (180 to 219)	6·73% (0·749 to 12·6)
	South Sudan	4440 (3380 to 6070)	75·8 (59·8 to 102)	8320 (6360 to 11 100)	87·8 (68·2 to 116)	87·6% (75·9 to 100)	15·9% (9·82 to 22·2)
	Tanzania	20 100 (15 300 to 27 400)	80·0 (62·3 to 106)	58 000 (45 000 to 77 400)	97·5 (77·2 to 127)	189% (173 to 209)	21·9% (16·1 to 29·3)
	Uganda	13 200 (10 100 to 17 900)	81·0 (63·8 to 107)	42 200 (32 900 to 55 400)	97·3 (77·0 to 125)	221% (201 to 243)	20·1% (13·3 to 27·6)
	Zambia	6580 (5110 to 8540)	85·3 (68·5 to 108)	20 100 (15 400 to 26 300)	100 (78·2 to 127)	205% (186 to 226)	17·4% (11·2 to 23·4)
Southern sub-Saharan Africa		60 900 (46 700 to 80 500)	111 (87·4 to 145)	92 800 (71 900 to 121 000)	107 (83·4 to 139)	52·5% (45·2 to 59·9)	−3·95% (−5·90 to −2·20)
	Botswana	1420 (1120 to 1890)	107 (83·8 to 138)	3010 (2340 to 3970)	113 (88·3 to 148)	111% (94·7 to 129)	5·41% (−0·414 to 11·2)
	Eswatini	796 (615 to 1050)	99·6 (78·5 to 131)	1400 (1090 to 1820)	110 (87·3 to 141)	76·0% (64·6 to 88·9)	10·2% (4·30 to 15·5)
	Lesotho	1390 (1080 to 1830)	93·5 (74·2 to 122)	2040 (1590 to 2640)	98·1 (77·9 to 126)	46·9% (37·7 to 56·4)	4·93% (−1·10 to 10·5)
	Namibia	1430 (1100 to 1900)	100 (79·0 to 132)	2980 (2280 to 3980)	113 (87·9 to 150)	108% (96·8 to 124)	12·4% (7·30 to 18·4)
	South Africa	45 800 (35 200 to 60 600)	116 (91·3 to 152)	67 000 (51 800 to 87 400)	108 (84·2 to 140)	46·2% (38·0 to 54·2)	−7·21% (−9·51 to −4·75)
	Zimbabwe	9980 (7600 to 13 200)	96·4 (74·3 to 125)	16 400 (12 600 to 21 500)	99·4 (78·0 to 129)	63·9% (53·3 to 76·4)	3·18% (−2·87 to 10·1)
Western sub-Saharan Africa		138 000 (106 000 to 187 000)	73·4 (57·8 to 97·5)	399 000 (304 000 to 548 000)	81·4 (63·9 to 109)	190% (181 to 197)	11·0% (8·18 to 13·3)
	Benin	3300 (2530 to 4500)	72·7 (57·0 to 96·7)	11 500 (8800 to 15 500)	85·4 (66·6 to 112)	250% (230 to 271)	17·4% (11·9 to 23·7)
	Burkina Faso	6700 (5230 to 8920)	76·1 (59·8 to 100)	19 000 (14 600 to 25 500)	85·1 (66·7 to 112)	184% (168 to 202)	11·8% (6·35 to 18·7)
	Cabo Verde	255 (187 to 354)	73·6 (54·9 to 99·6)	594 (451 to 793)	96·4 (74·6 to 128)	133% (113 to 158)	31·0% (24·4 to 39·2)
	Cameroon	7470 (5710 to 9940)	74·6 (58·6 to 97·8)	28 400 (21 600 to 39 300)	86·2 (67·3 to 117)	280% (258 to 305)	15·6% (9·35 to 22·4)
	Chad	4180 (3250 to 5550)	74·9 (59·7 to 98·7)	13 500 (10 300 to 18 200)	80·7 (63·1 to 107)	225% (207 to 244)	7·70% (2·42 to 13·8)
	Côte d'Ivoire	8690 (6510 to 11 900)	71·9 (55·8 to 95·9)	24 300 (18 700 to 32 800)	85·1 (66·2 to 113)	180% (165 to 197)	18·4% (12·6 to 24·4)
	The Gambia	753 (566 to 1020)	78·0 (60·9 to 103)	2260 (1700 to 3060)	89·8 (70·0 to 120)	199% (182 to 218)	15·1% (9·27 to 21·6)
	Ghana	13 900 (10 800 to 18 200)	94·2 (74·8 to 121)	38 700 (30 000 to 51 000)	109 (87·3 to 142)	178% (162 to 194)	15·8% (9·80 to 21·4)
	Guinea	4070 (3200 to 5440)	72·6 (57·6 to 95·6)	11 200 (8480 to 15 300)	83·4 (64·6 to 113)	176% (160 to 194)	14·8% (8·87 to 21·6)
	Guinea-Bissau	782 (614 to 1010)	79·8 (64·2 to 102)	1770 (1370 to 2380)	83·3 (65·9 to 110)	127% (112 to 142)	4·36% (−1·63 to 10·9)
	Liberia	1730 (1310 to 2340)	72·5 (56·0 to 96·0)	5240 (3980 to 7140)	91·8 (70·9 to 122)	202% (184 to 223)	26·7% (19·8 to 34·5)
	Mali	5810 (4470 to 7880)	71·8 (55·7 to 95·4)	19 400 (14 600 to 26 900)	83·1 (64·4 to 113)	235% (214 to 257)	15·8% (9·95 to 22·2)
	Mauritania	1520 (1180 to 2080)	76·9 (60·3 to 102)	4220 (3160 to 5800)	95·5 (73·7 to 129)	177% (161 to 193)	24·2% (16·5 to 31·0)
	Niger	5670 (4370 to 7480)	74·8 (58·8 to 97·1)	19 800 (15 000 to 26 700)	83·1 (64·8 to 111)	250% (228 to 277)	11·1% (5·17 to 18·2)
	Nigeria	61 600 (47 500 to 84 400)	69·3 (54·4 to 93·0)	169 000 (127 000 to 233 000)	73·1 (56·8 to 98·4)	174% (165 to 181)	5·60% (2·47 to 8·57)
	São Tomé and Príncipe	83·7 (63·0 to 116)	71·8 (55·0 to 97·7)	206 (155 to 279)	88·8 (68·2 to 119)	146% (129 to 163)	23·8% (18·2 to 30·0)
	Senegal	5620 (4320 to 7670)	76·7 (60·2 to 102)	14 400 (10 900 to 19 400)	87·8 (67·8 to 117)	156% (142 to 171)	14·5% (8·73 to 20·0)
	Sierra Leone	3050 (2310 to 4150)	74·1 (57·2 to 98·8)	8410 (6350 to 11 300)	89·7 (69·2 to 118)	176% (158 to 198)	21·1% (14·1 to 30·4)
	Togo	2640 (2000 to 3550)	74·0 (58·2 to 97·0)	7490 (5730 to 10 300)	87·0 (68·2 to 117)	184% (165 to 202)	17·7% (11·7 to 23·9)

Data in parentheses are 95% uncertainty intervals (UIs). Count data and rates are presented to three significant figures.

The spatial distribution of the age-standardised incidence rate of appendicitis in 2021 is shown in figure 2B. Of the seven GBD super-regions, the high-income super-region had the highest incidence rate, at 254 (95% UI 210–317) per 100 000, and the sub-Saharan Africa super-region had the lowest, at 87·1 (68·5–115) per 100 000 (table 2). High-income Asia Pacific had the highest age-standardised incidence rate, at 364 (286–475) per 100 000. The region with the second highest age-standardised incidence rate was Andean Latin America at 345 (273–433) per 100 000, followed by east Asia at 306 (243–405) per 100 000. The regions with the lowest age-standardised incidence rates were western sub-Saharan Africa (81·4 [63·9–109] per 100 000), eastern sub-Saharan Africa (87·5 [69·2–114] per 100 000), and central sub-Saharan Africa (93·0 [72·9–121] per 100 000). Age-standardised incidence rates also varied widely at the national level, ranging from 373 (296–480) per 100 000 in Japan to 68·8 (53·3–90·8) per 100 000 in Ethiopia. Of the 204 countries and territories, the mean incidence rates in 68 countries exceeded the global incidence rate of 214 (174–274) per 100 000; these were mainly the high-income and middle-income nations in high-income Asia Pacific, Australasia, western Europe, high-income North America, southern Latin America, Andean Latin America, central Latin America, east Asia, central Europe, and south Asia. Specifically, Japan had the highest age-standardised incidence rate of appendicitis in 2021 (373 [296–480] per 100 000), followed by Peru (372 [294–470] per 100 000), Belgium (369 [326–412] per 100 000), and Switzerland (363 [307–419] per 100 000). The bottom 25th percentile, 50 countries mostly in sub-Saharan Africa and Oceania, had incidence rates lower than 113 per 100 000. Specifically, Ethiopia, Nigeria, Chad, Kenya, Niger, and Mali had the lowest incidence rates of appendicitis in 2021, between 68·8 (53·3–90·8) per 100 000 in Ethiopia and 83·1 (64·4–113) per 100 000 in Mali.

While the global number of new cases increased substantially between 1990 and 2021, we observed a relatively stable global age-standardised incidence rate, from 219 (95% UI 176–283) per 100 000 in 1990 to 214 (174–274) per 100 000 in 2021. The temporal trends, however, varied widely across regions (figure 1). Of the 21 GBD regions, all except four (central Europe, eastern Europe, western Europe, and high-income Asia Pacific) saw a significant increase in the crude number of incident cases. The increase in the crude number of incident cases was the greatest in three regions in sub-Saharan Africa (central, western, and eastern sub-Saharan Africa). Central sub-Saharan Africa, in particular, had an increase of 233% (221–248). The age-standardised incidence rate decreased significantly in three regions (Andean Latin America, south Asia, and sub-Saharan Africa). The incidence rate increased most significantly in central sub-Saharan Africa (27·5% [23·3–32·4]) and east Asia (25·4% [19·4–31·4]). High-income North America was the only region in which, both the crude number and age-standardised rate of incidence remained largely unchanged across time.

**Figure 3:**
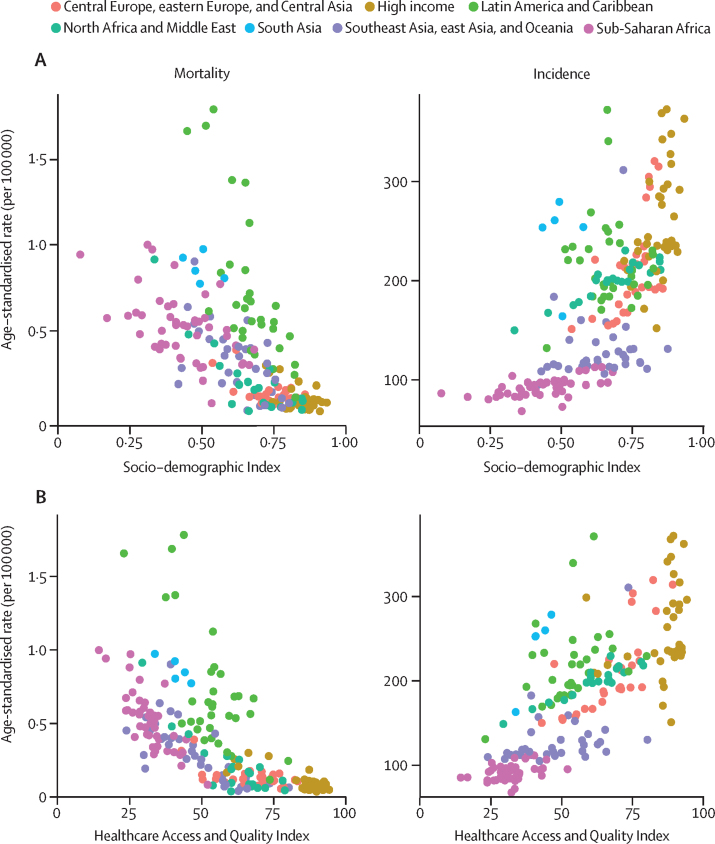
Age-standardised rates of appendicitis mortality and incidence in 2021 (A) Based on Socio-demographic Index, colour-coded by GBD super-region. (B) Based on Healthcare Access and Quality Index, colour-coded by GBD super-region. GBD=Global Burden of Diseases, Injuries, and Risk Factors Study.

There was a clear, high correlation between SDI and appendicitis burden in 2021 (figure 3). A similar pattern was found for HAQ Index. We observed a significant negative association between SDI and mortality due to appendicitis in 2021, where countries with a higher national SDI had lower mortality rates than those with a lower SDI. Conversely, there was a significant positive association between SDI and incidence of appendicitis in the same year, where countries with a higher national SDI had higher appendicitis incidence than those with a lower SDI. There was a similar geographical pattern of mortality and incidence against HAQ Index.

Globally, appendicitis resulted in 1·13 million (95% UI 0·953–1·31) YLLs, 203 000 YLDs (130 000–288 000), and 1·33 million (1·15–1·57) DALYs in 2021 (results are accessible through the GBD Compare tool). Whereas the global number of YLLs attributable to appendicitis decreased by 39·7% from 1·90 million (1·47–2·30) in 1990, the global number of YLDs increased by 40·9% from 144 000 (90 700–209 500) in 1990. When controlling for population growth and ageing, YLL rates decreased by 60·3% and YLD rates decreased by 1·64% between 1990 and 2021.

The rank of YLLs and YLDs differed considerably across regions (results are accessible through the GBD Compare tool). Whereas YLL rankings were higher in LMICs, YLD rankings were higher in high-income countries. The highest age-standardised rates of YLLs were observed in the Caribbean (36·6 [95% UI 25·8–50·3] per 100 000), Andean Latin America (33·9 [30·2–38·2] per 100 000), central Latin America (33·6 [26·3–43·4] per 100 000), and south Asia (28·9 [22·7–37·8] per 100 000; data from GBD compare tool). The lowest rates of YLLs were observed in high-income Asia Pacific (1·41 [1·23–1·63] per 100 000), Australasia (1·88 [1·65–2·13] per 100 000), and western Europe (2·03 [1·88–2·19] per 100 000). Of the high-income regions, southern Latin America had the highest rate of YLLs, at 8·34 (7·36–9·53) per 100 000, which was higher than that of Oceania. For YLDs, high-income Asia Pacific had the highest age-standardised rate of YLDs, at 4·34 (2·65–6·31) per 100 000, whereas western, eastern, southern, and central sub-Saharan Africa and Oceania had the lowest YLD rates, all lower than 1·5 per 100 000.

**Figure 4:**
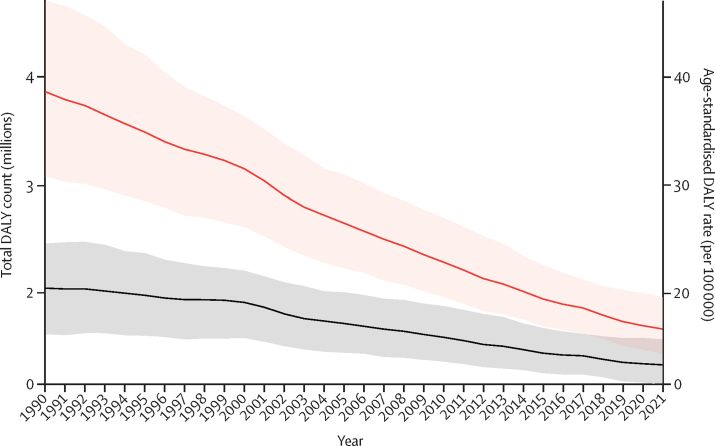
Global temporal trend of DALYs due to appendicitis between 1990 and 2021 The black line represents the total DALY counts in millions, with the grey shading representing the 95% uncertainty intervals. The red line represents the total DALY counts in millions, with the light red shading representing the 95% uncertainty intervals. DALYs=disability-adjusted life-years.

**Figure 5:**
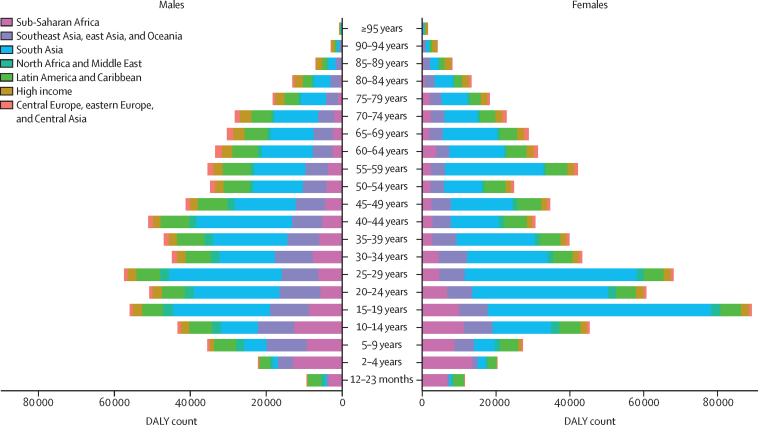
Distribution of absolute DALYs due to appendicitis in 2021 by age group, sex, and GBD super-region DALYs=disability-adjusted life-years. GBD=Global Burden of Diseases, Injuries, and Risk Factors Study.

Despite the geographical variation in YLLs and YLDs, we found that both the absolute number and age-standardised rate of DALYs, which is the sum of YLLs and YLDs, decreased steadily between 1990 and 2021, showing improvement in overall appendicitis-related health (figure 4). Overall, the annualised rate of decline was greatest in children aged younger than 10 years for both males and females (table 3). Although appendicitis incidence was greatest in the second and third decades of life, there were geographical variations in terms of which age group carried the greatest appendicitis-related burden (figure 5). In sub-Saharan Africa, the largest burden, as expressed in DALY counts, was concentrated in children aged younger than 10 years, whereas in high-income regions, people aged older than 80 years carried the greatest burden.

**Table 3:** Annualised rate of change of disability-adjusted life-years due to appendicitis between 1990 and 2021, by sex and age group

	**Male, % (95% UI)**	**Female, % (95% UI)**
12 to 23 months	−5·77% (−7·22 to −3·94)	−4·80% (−6·53 to −3·31)
2 to 4 years	−4·92% (−6·32 to −2·56)	−4·60% (−6·35 to −2·81)
5 to 9 years	−4·42% (−5·35 to −2·68)	−3·85% (−4·78 to −2·45)
10 to 14 years	−2·95% (−3·65 to −1·82)	−2·35% (−3·09 to −1·35)
15 to 19 years	−2·65% (−3·45 to −1·39)	−2·14% (−3·29 to −0·849)
20 to 24 years	−2·30% (−3·20 to −0·924)	−1·95% (−3·19 to −0·661)
25 to 29 years	−2·49% (−3·33 to −1·20)	−2·19% (−3·46 to −1·02)
30 to 34 years	−2·06% (−2·79 to −1·01)	−1·99% (−3·03 to −1·01)
35 to 39 years	−2·30% (−3·19 to –1·21)	−2·32% (−3·54 to −1·15)
40 to 44 years	−2·52% (−3·39 to −1·21)	−2·12% (−3·20 to −1·05)
45 to 49 years	−2·72% (−3·59 to −1·54)	−2·50% (−3·86 to −1·21)
50 to 54 years	−2·87% (−3·69 to −1·77)	−2·52% (−3·70 to −1·38)
55 to 59 years	−2·80% (−3·81 to −1·54)	−2·27% (−4·07 to −0·815)
60 to 64 years	−2·77% (−3·79 to −1·56)	−2·30% (−3·90 to −1·04)
65 to 69 years	−2·71% (−3·84 to −1·51)	−2·22% (−3·95 to −0·938)
70 to 74 years	−2·67% (−4·01 to −1·42)	−2·34% (−3·92 to −1·14)
75 to 79 years	−2·48% (−3·69 to −1·39)	−2·11% (−3·62 to −0·975)
80 to 84 years	−2·38% (−3·77 to −1·28)	−2·05% (−3·57 to −0·935)
85 to 89 years	−2·09% (−3·46 to −1·04)	−2·08% (−3·36 to −1·02)
90 to 94 years	−1·96% (−3·33 to −0·899)	−1·92% (−3·32 to −0·950)
≥95 years	−2·04% (−3·40 to −1·03)	−1·60% (−3·29 to −0·620)

Data are presented to three significant figures.

## Discussion

This study provides the most up-to-date, comprehensive assessment of appendicitis mortality and morbidity between 1990 and 2021 at the global, regional, and national levels. As measured by changes in DALYs, our research shows an overall decline in the global burden of appendicitis over the past three decades, with the mortality rate having decreased more than the incidence rate. Despite the encouraging trends in the global burden, substantial variation exists across countries. In 2021, the countries with the highest incidence rates of appendicitis were primarily located in high-income Asia Pacific, western Europe, and Andean Latin America, and those with the lowest incidence were concentrated in sub-Saharan Africa and Oceania. Consistent with previous studies,^20^, ^21^, ^22^, ^23^, ^24^, ^25^ we generally observed higher incidence rates for appendicitis in wealthier countries, which have a higher SDI. By contrast, higher mortality rates for appendicitis, although also seen in some countries with moderate or high incidence in Latin America and the Caribbean, were largely clustered in low-incidence countries of south Asia and sub-Saharan Africa, typically where SDI is low. This contrast between incidence and mortality in temporal trends and SDI pattern could be due to variation in underlying determinants, inadequate detection of cases when and where resources are scarce, or a combination of both. To better guide policy formulation, it is necessary to comprehend the drivers of these temporal and national patterns.

The exact cause of appendicitis is unknown. There are, however, genetic, lifestyle, and environmental factors that might explain some of the spatial and temporal variations in appendicitis incidence observed in this study. Earlier studies have found that appendicitis incidence is higher in individuals with a family history of appendicitis, suggesting a genetic contribution to the disease,^46^, ^47^ which could be irregularly distributed globally. The adoption of western dietary habits—characterised by low fibre and high sugar intake—has also been associated with an increased risk of appendicitis,^48^, ^49^, ^50^ potentially contributing to the high incidence of appendicitis observed in many developed countries, as well as the rising incidence in rapidly developing nations, including those in sub-Saharan Africa. Beyond these genetic and dietary factors, various lifestyle and environmental factors, including exposure to cigarette smoke,^51^ ambient air pollution,^52^, ^53^ and warmer temperature,^54^, ^55^, ^56^ might also influence the observed geographical and temporal variations in the incidence of appendicitis.

By contrast, higher mortality rates in low-incidence, low SDI countries suggest higher case fatality due to health-systems factors, such as inadequate hospital care, pre-hospital delays (ie, delays in accessing care), and insufficient diagnostic tools,^57^, ^58^ as well as poor surgical facilities and surgical outcomes for those who are diagnosed and treated. In areas with poor access to quality health care, people might be less inclined to seek medical care for symptoms of appendicitis than those in high-income areas for various reasons, such as long distances to health facilities, absence of reliable transportation, and low financial resources.^59^, ^60^, ^61^, ^62^ Studies have found that a high proportion of people with appendicitis presenting to hospitals in LMICs had a prolonged pre-hospital delay.^59^, ^63^, ^64^, ^65^ A study conducted in Malawi, for instance, showed that only 38% of patients with appendicitis presented to the hospital within 3 days of symptom onset, and nearly 50% of patients had a pre-hospital delay of more than 1 week.^66^ Evidence has consistently shown that delayed clinical presentation substantially increases illness severity and the risk of complications. Therefore, even when a surgical operation is performed, the risk of postoperative complications and mortality is often much higher in lower-income countries than in higher-income countries.^59^, ^63^, ^64^, ^65^, ^67^, ^68^ These plausible drivers of mortality rate differences across countries could also drive the improving mortality rates observed over time in most countries and might reflect improving and more timely access to health care, a rise in accurate and early diagnosis, enhanced safety of appendicectomy, and rising access to minimally invasive treatments. Notably, these health-systems limitations have a special impact on mortality in younger age groups: paediatric appendicitis is particularly challenging to diagnose for reasons such as non-specific symptoms and difficulty obtaining a reliable medical history and physical examination.^69^, ^70^ In the absence of imaging capabilities, these challenges make diagnosis even more difficult in resource-constrained areas with younger populations.

Although the appendicitis burden is not on the same order of magnitude as those infectious diseases that are frequently targeted by vertical elimination programmes (appendicitis contributed 0·05% of global DALYs in 2019 in contrast to 1·7% contributed by HIV, as per the GBD Compare Tool), it should be noted that appendicitis typifies a group of gastrointestinal and genitourinary diseases amenable to surgical cure that collectively make an appreciable contribution to global DALYs (0·7% in 2019 from gallbladder and biliary diseases; urolithiasis; appendicitis; inguinal, femoral, and abdominal hernia; paralytic ileus and intestinal obstruction; and vascular intestinal disorders combined) and are all rising in incidence in low SDI countries. Policy makers and donors need estimates of the levels and trends of these disorders to weigh the advantages of disease-specific vertical programmes against investments aimed at enhancing adaptable and responsive health systems—such as access to primary and urgent care, adequate imaging equipment and expertise in interpretation, pharmacy supply chains, and equipment and trained personnel for anaesthesia and surgery—to adopt comprehensive health-care management strategies capable of addressing a diverse range of conditions.

Although the geographical and temporal trends in appendicitis burden in our study generally follow the socioeconomic status of a country, a few notable exceptions exist. Our analysis shows that Andean Latin America, composed of three countries (Bolivia, Ecuador, and Peru), has one of the highest incidence and mortality rates across all GBD regions; however, this region also had the biggest success in reducing the disease burden (ie, the DALY rate) during the study period. The DALY rate similarly decreased in south Asia. Further epidemiological research in these regions would help shed light on the role of underlying risk factors and clinical practice in the heterogeneity of the disease burden.

Despite the similar spatial pattern, the incidence rates estimated in our study were generally higher than those previously reported. The difference in the magnitude could be explained partly by the efforts we made to ensure that inpatient data sources were standardised and corrected to account for patients who did not require an inpatient admission lasting at least 24 h. Several studies have found that, in high-income countries, a considerable proportion of patients are diagnosed and treated in outpatient and same-day hospital settings. In the USA, for instance, about 20–37% of paediatric laparoscopic appendicectomies were found to be done in outpatient care and patients were discharged on the same day.^71^, ^72^ Likewise, in the UK, 45% of adult patients with acute appendicitis were treated and discharged from outpatient care on the same day.^73^ A similar pattern was observed in our claims data sources, which distinguished inpatient claims (lasting at least 24 h and having a room and board claim) from outpatient claims (which included same-day care from clinics and hospital services); in our sources, 56% of total appendicitis cases were captured in the outpatient claims file only (data not shown). As outpatient treatments and procedures are becoming more common to treat non-perforated appendicitis, especially in high-income countries, it is important to take into consideration outpatient cases to accurately estimate the total disease burden.

There are several limitations to this study, primarily stemming from the limited quantity and high heterogeneity of input data, which our analyses were only partially able to overcome. Although we amassed a large base of international vital registration, verbal autopsy, and clinical administrative data, there were still many locations and years for which primary data were unavailable, particularly for countries in Latin America, central Asia, southeast Asia, central Europe, sub-Saharan Africa, and north Africa and the Middle East. High-quality primary data are key in advancing scientific knowledge of appendicitis, but without modelling there is a risk that the absence of primary data might be misconstrued as an absence of burden. Thus, where mortality data were absent, CODEm generated estimates from regression models that incorporated information from predictive covariates, nearby countries, and adjacent years, selecting the models that best predicted mortality for held-out data. The higher uncertainty in estimates for locations and years without data must be borne in mind and serve as an impetus for improved data systems.

As mentioned, incidence data are even more sparse and heterogeneous than mortality data. To address sparsity, we use DisMod, which combines regression (to leverage information from predictive covariates and regional patterns) with compartmental disease model logic (to leverage information about the relationship between incidence and mortality) to produce incidence estimates for locations and years without data. This approach relies on the comparability of incidence data sources and a predictable relationship between incidence and mortality across locations and years. Our incidence data come from clinical administrative databases and uses common classification systems (ICD-9 and ICD-10), but can differ by various factors, such as health-care-seeking behaviours, health-care access, coding practices, levels of misclassification, average length of stay for appendicitis treatment, and how same-day care provided by hospitals is categorised, rather than true differences in the underlying incidence of appendicitis.^74^, ^75^, ^76^, ^77^, ^78^ We aimed to overcome some of this heterogeneity by restricting inpatient discharge data to only encounters lasting more than 24 h, and leveraging a smaller claims dataset to estimate the ratio between inpatient discharges and all appendicitis cases receiving care across all health-care settings, and applying this ratio to inpatient discharge data. This technique might account for variation in whether less-severe cases are included in inpatient databases but does not attempt to account for variation in whether more-severe cases make it to inpatient care (eg, variation in pre-hospital mortality). Any undercounting of incident cases due to pre-hospital mortality would be carried forward into the production of EMR inputs for DisMod modelling as well, resulting in over-estimation of EMR and further contributing to under-estimation of incidence in some data-sparse locations. Both estimation of the ratio of inpatient discharges to total cases and of EMR require some tradeoff between the comparability of input data and range of health-care systems they represent; in future iterations, sensitivity analyses could be performed to assess the influence of this tradeoff. Even after these steps to correct sources of heterogeneity, some implausible values remained, and we systematically excluded as outliers any data sources with age-standardised incidence rates greater or less than two median absolute deviations from the median of the age-standardised incidence rates of all data—an approach that does not differentiate between heterogeneity stemming from the data sources and heterogeneity originating from the underlying disease itself.

Data describing disease duration and severity, required for YLD estimation, are the most sparse data components of all. In our non-fatal burden estimation, we applied a uniform duration and disability weight across all demographics: age, sex, year, and location. We suspect, however, that the variation in health-care access and quality that affects appendicitis mortality rates might also drive variation in the duration and severity of the disease over time and between locations. In future iterations of GBD, it would be beneficial to identify and incorporate information about temporal and spatial variation in disease duration and severity.

The 95% UIs on final burden estimates included sampling error in underlying incidence and causes of death data: sampling error, between-study heterogeneity, and statistical uncertainty in model-fitting and prediction for data-processing steps (garbage code redistribution, inpatient data correction factors, and production of CSMR and EMR inputs to DisMod models); statistical uncertainty in model-fitting and prediction for incidence and mortality estimates; and propagation of uncertainty through post-regression scaling and weighting steps (CoDCorrect, disability weighting, and comorbidity adjustment). However, it was too computationally intensive to incorporate uncertainty from covariate and population estimates, which have their own modelling strategies to produce age-sex-year-location-specific values.

A final limitation relates to ICD-based case definitions. We used a broader set of ICD codes to define appendicitis cases in this analysis compared with earlier studies.^78^ This approach might have resulted in inclusion of other forms of appendiceal diseases. Furthermore, in our analysis we combined non-perforating and perforating appendicitis together due to the inability to differentiate these two types of appendicitis in the available input data. We acknowledge that these two types of appendicitis have unique differences in severity, and hence morbidity and mortality.^79^, ^80^, ^81^ In future iterations of GBD, consideration should be given to distinguishing between these two types of appendicitis.

In conclusion, our study shows that the global burden of appendicitis has been declining steadily in the past three decades. However, important geographical variations exist in the fatal and non-fatal burden of appendicitis that highlight the disparities in health-care access that persist in many parts of the world. The rising incidence of appendicitis in many LMICs will require improved health-care access and an infrastructure that supports rapid and accurate diagnosis and timely delivery of treatment.

### GBD 2021 Appendicitis Collaborator Group

### Affiliations

### Contributors

### Data sharing

This study follows the Guidelines for Accurate and Transparent Health Estimates Reporting (GATHER). To download the data used in these analyses, please visit the Global Health Data Exchange (GHDx). https://ghdx.healthdata.org/gbd-2021

## Declaration of interests

M Lee reports support for the present manuscript from the Ministry of Education of the Republic of Korea and the National Research Foundation of Korea (NRF-2023S1A3A2A05095298) and Bio-convergence Technology Education Program through the Korea Institute for Advancement Technology (KIAT) funded by the Ministry of Trade, Industry and Energy (No. P0017805). M-C Li reports grants or contracts from the National Science and Technology Council in Taiwan (NSTC 112-2410-H-003-031); leadership or fiduciary roles in board, society, committee or advocacy groups, paid or unpaid with the *Journal of the American Heart Association* as a Technical Editor; outside the submitted work. L Monasta reports support for the present manuscript from the Italian Ministry of Health (Ricerca Corrente 34/2017), payments made to the Institute for Maternal and Child Health IRCCS Burlo Garofolo. J Sanabria reports support for attending meetings and/or travel from Marshall University School of Medicine; Participation on a Data Safety Monitoring Board or Advisory Board from Marshall University School of Medicine; leadership or fiduciary roles in board, society, committee or advocacy groups, paid or unpaid with SSO, SSAT, AASLD, IHPBA, ACS, and ABS; outside the submitted work. JA Singh reports consulting fees from ROMTech, Atheneum, Clearview healthcare partners, American College of Rheumatology, Yale, Hulio, Horizon Pharmaceuticals, DINORA, Frictionless Solutions, Schipher, Crealta/Horizon, Medisys, Fidia, PK Med, Two labs, Adept Field Solutions, Clinical Care options, Putnam associates, Focus forward, Navigant consulting, Spherix, MedIQ, Jupiter Life Science, UBM LLC, Trio Health, Medscape, WebMD, and Practice Point communications; and the National Institutes of Health; payment or honoraria for lectures, presentations, speakers bureaus, manuscript writing or educational events on the speakers bureau of Simply Speaking; support for attending meetings and/or travel from OMERACT as a steering committee member; participation on a data safety monitoring board or advisory board with the FDA Arthritis Advisory Committee; leadership or fiduciary role in other board, society, committee or advocacy group, paid as a past steering committee member of the OMERACT, an international organization that develops measures for clinical trials and receives arm's length funding from 12 pharmaceutical companies, unpaid as Chair of the Veterans Affairs Rheumatology Field Advisory Committee, and unpaid as the Editor and Director of the UAB Cochrane Musculoskeletal Group Satellite Center on Network Meta-analysis; stock or stock options in Atai life sciences, Kintara therapeutics, Intelligent Biosolutions, Acumen pharmaceutical, TPT Global Tech, Vaxart pharmaceuticals, Atyu biopharma, Adaptimmune Therapeutics, GeoVax Labs, Pieris Pharmaceuticals, Enzolytics, Seres Therapeutics, Tonix Pharmaceuticals Holding Corp, Aebona Pharmaceuticals, and Charlotte's Web Holdings, and previously owned stock options in Amarin, Viking, and Moderna Pharmaceuticals outside the submitted work. JHV Ticoalu reports other financial or non-financial interests as a co-founder of Benang Merah Research Center outside the submitted work. The other authors declare no competing interests.
