# Supplementary material for: Trends and levels of the global, regional, and national burden of appendicitis between 1990 and 2021: findings from the Global Burden of Disease Study 2021
Source: Lancet Gastroenterol Hepatol. 2024 Jul 17;9(9):825–58. doi: 10.1016/S2468-1253(24)00157-2 (PMC11306195; doi:10.1016/S2468-1253(24)00157-2)
Supplement: Supplementary appendix [file mmc1.pdf]

THE LANCET
Gastroenterology & Hepatology

Supplementary appendix

This appendix formed part of the original submission and has been peer reviewed. We post it as supplied by the authors.

Supplement to: GBD 2021 Appendicitis Collaborator Group. Trends and levels of the global, regional, and national burden of appendicitis between 1990 and 2021: findings from the Global Burden of Disease Study 2021. *Lancet Gastroenterol Hepatol* 2024; published online July 17. https://doi.org/10.1016/S2468-1253(24)00157-2.

Appendix 1: Supplementary methods and results to "The global, regional, and national burden of appendicitis between 1990 and 2021: a systematic analysis for the Global Burden of Disease Study 2021"

Table of Contents

Section 1. Statement of GATHER compliance 3

Table S1. Guidelines for Accurate and Transparent Health Estimates Reporting (GATHER) checklist 3

Section 2. Demographics estimated in the Global Burden of Disease Study 4

Table S2. Geographical hierarchy 4

Section 3. Standard population in the Global Burden of Disease Study 5

Section 4. Fatal estimation 5

4.1 Flowchart 5

Figure S1. Flowchart of mortality and YLL estimation of appendicitis 5

4.2 Data identification and processing 5

Figure S2. Data coverage for estimating mortality of appendicitis 6

Table S3. International Classification of Diseases (ICD) codes mapped to Global Burden of Disease cause list for appendicitis mortality data 6

4.3 Modelling 6

Table S4. Covariates used in appendicitis mortality modelling 7

4.4 Summary statistical metrics on model performance 9

Table S5. Summary statistical metrics on ensemble model performance of appendicitis 9

Section 5. Non-fatal estimation 9

5.1 Flowchart 9

Figure S3. Flow diagram of non-fatal health outcome estimation for appendicitis 9

5.2 Data identification and processing 10

5.2.1 Incidence data 10

Figure S4. Data coverage for estimating incidence of appendicitis 10

Table S6. International Classification of Diseases (ICD) codes mapped to Global Burden of Disease cause list for appendicitis incidence data 11

Table S7. MR-BRT crosswalk adjustment factors for appendicitis 12

5.2.2 Excess mortality estimates 13

5.2.3 Cause-specific mortality rates 13

5.3 Modelling 13

Table S8. Summary of covariates used in the appendicitis DisMod-MR meta-regression model 14

5.4 Summary statistical metrics on model performance 14
Table S9. Summary statistical metrics on DisMod model performance of appendicitis 14
5.5 Disability weight 14
Table S10. Disability weight and health state of appendicitis 14
Section 6. Additional Figures 15
Figure S5. Global temporal trend of years of life lost (YLLs) of appendicitis between 1990 and 2021 15
Figure S6. Global temporal trend of years lived with disability (YLDs) of appendicitis between 1990 and 2021 15
References 16

Section 1. Statement of GATHER compliance

This study complies with the Guideline for Accurate and Transparent Health Estimates Reporting (GATHER) recommendations. See table S1 below for the GATHER checklist. The GATHER recommendations can be found on the GATHER website (https://www.who.int/data/gather).

Table S1. Guidelines for Accurate and Transparent Health Estimates Reporting (GATHER) checklist

Item #	Checklist item	Reported on page #
**Objectives and funding**		
**1**	Define the indicator(s), populations (including age, sex, and geographic entities), and time period(s) for which estimates were made.	Appendix pp 4-5
**2**	List the funding sources for the work.	pp 12
Data Inputs		
For all data inputs from multiple sources that are synthesized as part of the study:		
3	Describe how the data were identified and how the data were accessed.	pp 7, 9-10
4	Specify the inclusion and exclusion criteria. Identify all ad-hoc exclusions.	pp 10
5	Provide information on all included data sources and their main characteristics. For each data source used, report reference information or contact name/institution, population represented, data collection method, year(s) of data collection, sex and age range, diagnostic criteria or measurement method, and sample size, as relevant.	Global Health Data Exchange (GHDx) (http://ghdx.healthdata.org/)
6	Identify and describe any categories of input data that have potentially important biases (e.g., based on characteristics listed in item 5).	pp 7, 9-10
For data inputs that contribute to the analysis but were not synthesized as part of the study:		
7	Describe and give sources for any other data inputs.	None
For all data inputs:		
8	Provide all data inputs in a file format from which data can be efficiently extracted (e.g., a spreadsheet rather than a PDF), including all relevant meta-data listed in item 5. For any data inputs that cannot be shared because of ethical or legal reasons, such as third-party ownership, provide a contact name or the name of the institution that retains the right to the data.	Global Health Data Exchange (GHDx) (http://ghdx.healthdata.org/)<br>Epi Visualization | Viz Hub (http://ihmeuw.org/61ls)<br>CoD Visualization | Viz Hub (http://ihmeuw.org/61ii)
Data analysis		
9	Provide a conceptual overview of the data analysis method. A diagram may be helpful.	Appendix pp 6, 9<br>Reference 1
10	Provide a detailed description of all steps of the analysis, including mathematical formulae. This description should cover, as relevant, data cleaning, data pre-processing, data adjustments and weighting of data sources, and mathematical or statistical model(s).	pp 8, 11<br>Appendix pp 7-11<br>Reference 1
11	Describe how candidate models were evaluated and how the final model(s) were selected.	pp 8, 11<br>Appendix pp 7-11<br>References 1, 2, 3, 15
12	Provide the results of an evaluation of model performance, if done, as well as the results of any relevant sensitivity analysis.	pp 8, 11<br>Appendix pp 7-11<br>References 1, 2, 3, 15
13	Describe methods for calculating uncertainty of the estimates. State which sources of uncertainty were, and were not, accounted for in the uncertainty analysis.	pp 6
14	State how analytic or statistical source code used to generate estimates can be accessed.	Global Health Data Exchange (GHDx) (https://ghdx.healthdata.org/gbd-2019/code)
**Results and Discussion**		
15	Provide published estimates in a file format from which data can be efficiently extracted.	GBD Compare | Viz Hub (http://ihmeuw.org/61lu)
16	Report a quantitative measure of the uncertainty of the estimates (e.g. uncertainty intervals).	95% uncertainty intervals provided for all reported estimates in text and tables
17	Interpret results in light of existing evidence. If updating a previous set of estimates, describe the reasons for changes in estimates.	pp 5-6
18	Discuss limitations of the estimates. Include a discussion of any modelling assumptions or data limitations that affect interpretation of the estimates.	pp 444-499

Section 2. Demographics estimated in the Global Burden of Disease Study

In GBD, we estimated the burden of appendicitis for age groups between 1 and 99+ years, for males and females, between 1990 and 2021, for 204 countries and territories, and for subnational locations within a subset of countries. Country-level results are reported in this manuscript. A list of countries and how they fit into the GBD geographical hierarchy is shown in below. Countries marked with * are estimated at a subnational level; subnational estimates can be accessed via GBD Compare (http://ihmeuw.org/61lw), GBD Results Tool (https://vizhub.healthdata.org/gbd-results/), and model-specific visualisations (Epi Visualization http://ihmeuw.org/61ls and CoD Visualization http://ihmeuw.org/61ii).

Table S2. Geographical hierarchy

Super-region	Region	Country or territory
Central Europe, eastern Europe, and central Asia	Central Asia	Armenia, Azerbaijan, Georgia, Kazakhstan, Kyrgyzstan, Mongolia, Tajikistan, Turkmenistan, Uzbekistan
	Central Europe	Albania, Bosnia and Herzegovina, Bulgaria, Croatia, Czech Republic, Hungary, Montenegro, North Macedonia, Poland*, Romania, Serbia, Slovakia, Slovenia
	Eastern Europe	Belarus, Estonia, Latvia, Lithuania, Moldova, Russia*, Ukraine
High-income	Australasia	Australia, New Zealand* (subnational Māori + non-Māori)
	High-income Asia Pacific	Brunei, Japan*, Singapore, South Korea
	High-income North America	Canada, Greenland, United States*
	Southern Latin America	Argentina, Chile, Uruguay
	Western Europe	Andorra, Austria, Belgium, Cyprus, Denmark, Finland, France, Germany, Greece, Iceland, Ireland, Israel, Italy*, Luxembourg, Malta, Monaco, Netherlands, Norway*, Portugal, San Marino, Spain, Switzerland, Sweden*, United Kingdom*
Latin America and Caribbean	Andean Latin America	Bolivia, Ecuador, Peru
	Caribbean	Antigua and Barbuda, Bahamas, Barbados, Belize, Bermuda, Cuba, Dominica, Dominican Republic, Grenada, Guyana, Haiti, Jamaica, Puerto Rico, Saint Kitts and Nevis, Saint Lucia, Saint Vincent and the Grenadines, Suriname, Trinidad and Tobago, Virgin Islands
	Central Latin America	Colombia, Costa Rica, El Salvador, Guatemala, Honduras, Mexico*, Nicaragua, Panama, Venezuela
	Tropical Latin America	Brazil*, Paraguay
North Africa and Middle East	North Africa and Middle East	Afghanistan, Algeria, Bahrain, Egypt, Iran*, Iraq, Jordan, Kuwait, Lebanon, Libya, Morocco, Oman, Palestine, Qatar, Saudi Arabia, Sudan, Syria, Tunisia, Türkiye, United Arab Emirates, Yemen
South Asia	South Asia	Bangladesh, Bhutan, India*, Nepal, Pakistan*
Southeast Asia, east Asia, Oceania	East Asia	China, North Korea, Taiwan (province of China)
	Oceania	American Samoa, Cook Islands, Federated States of Micronesia, Fiji, Guam, Kiribati, Marshall Islands, Nauru, Niue, Northern Mariana Islands, Palau, Papua New Guinea, Samoa, Solomon Islands, Tokelau, Tonga, Tuvalu, Vanuatu
	Southeast Asia	Cambodia, Indonesia*, Laos, Malaysia, Maldives, Mauritius, Myanmar, Philippines*, Seychelles, Sri Lanka, Thailand, Timor-Leste, Viet Nam
Sub-Saharan Africa	Central sub-Saharan Africa	Angola, Central African Republic, Congo (Brazzaville), Democratic Republic of the Congo, Equatorial Guinea, Gabon
	Eastern sub-Saharan Africa	Burundi, Comoros, Djibouti, Eritrea, Ethiopia*, Kenya*, Madagascar, Malawi, Mozambique, Rwanda, Somalia, South Sudan, Tanzania, Uganda, Zambia
	Southern sub-Saharan Africa	Botswana, Eswatini, Lesotho, Namibia, South Africa*, Zimbabwe
	Western sub-Saharan Africa	Benin, Burkina Faso, Cape Verde, Cameroon, Chad, Côte d'Ivoire, Gambia, Ghana, Guinea, Guinea-Bissau, Liberia, Mali, Mauritania, Niger, Nigeria*, São Tomé and Príncipe, Senegal, Sierra Leone, Togo

Section 3. Standard population in the Global Burden of Disease Study

Per GBD standards, age-standardisation was performed using the direct method, as described by Ahmad and colleagues for WHO in 2001.[1] That is to say, we aggregated year-age-sex-location-specific estimates to the age-sex distribution of a reference population, specifically, the GBD world population age standard. This world population age standard uses the non-weighted mean of GBD 2021's age-specific population proportional distributions for all national locations with a population greater than 5 million people in 2019 (non-pandemic year). The population estimates used for GBD 2021 will be reported in GBD 2021 Demographics Collaborators. Global age-sex-specific mortality, life expectancy, and population estimates in 204 countries and 811 subnational locations, 1950-2021: a comprehensive demographic analysis for the Global Burden of Disease Study 2021. *Lancet (accepted),*[2] but methods are broadly similar to GBD 2019.[3]

Section 4. Fatal estimation

4.1 Flowchart

The flowchart below describes the mortality and YLL estimation processes used for appendicitis in the Global Burden of Disease (GBD) study.

Figure S1. Flowchart of mortality and YLL estimation of appendicitis

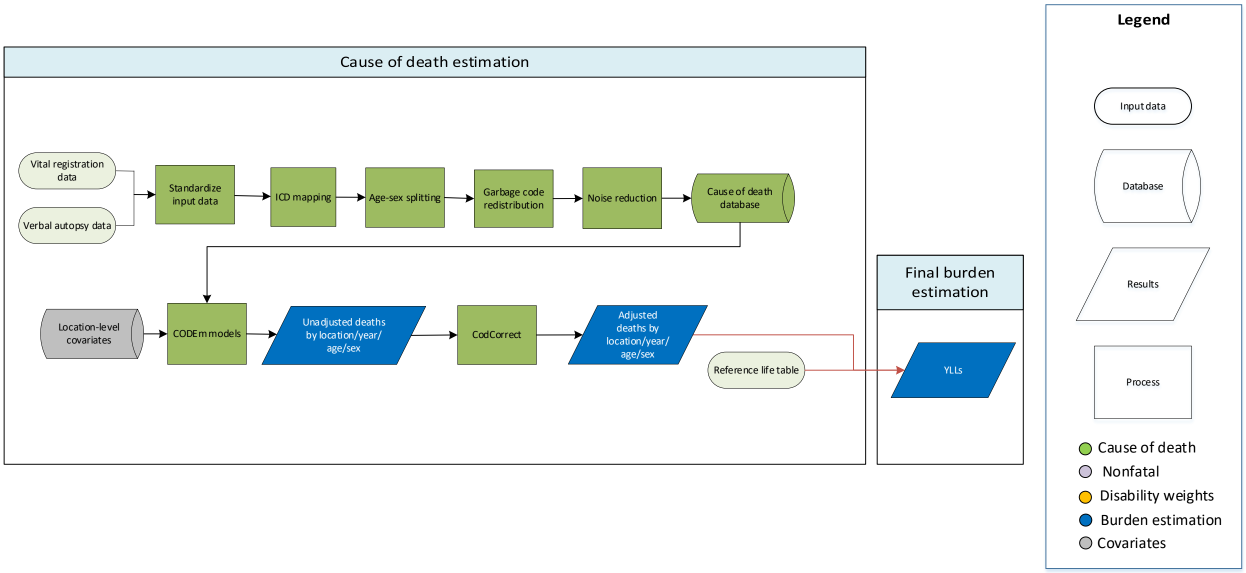

4.2 Data identification and processing

We used vital registration and verbal autopsy data to model appendicitis mortality. Specifically, causes of death (CoD) data were extracted from 870 sources covering 134 countries and territories, as shown in the coverage map in Figure S2. The International Classification of Diseases (ICD) codes mapped to appendicitis are shown in Table S3.

A detailed description of processing of CoD data for GBD has been published previously.[4,5] It follows the standard processing steps that are applicable to all GBD-defined causes, including ICD mapping, disaggregation of aggregated data, age-sex splitting, corrections for misclassification, redistribution of garbage codes, and noise reduction.

Figure S2. Data coverage for estimating mortality of appendicitis

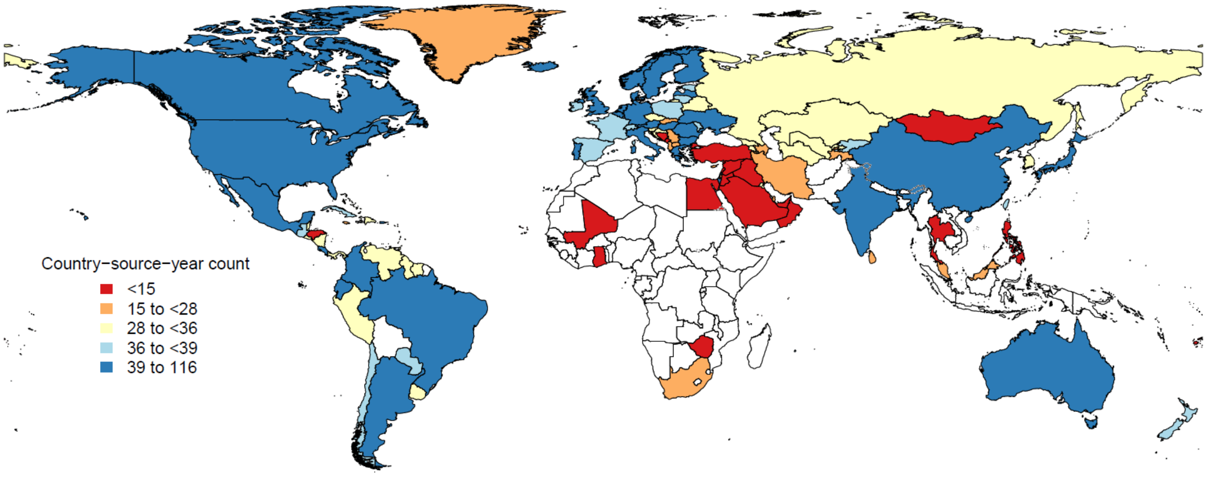

Table S3. International Classification of Diseases (ICD) codes mapped to Global Burden of Disease cause list for appendicitis mortality data

ICD system	ICD codes
10	K35 (K35, K35.0, K35.1, K35.2, K35.3, K35.8, K35.80, K35.89, K35.9)<br>K36 (K36, K36.0)<br>K37 (K37, K37.0, K37.9)<br>K38.3, K38.8, K39.9
9	540 (540, 540.0, 540.1, 540.9)<br>541 (541, 541.0, 541.1, 541.2, 541.3, 541.9)<br>542 (542, 542.0, 542.1, 542.9)

Outliers were identified by systematic examination of datapoints for all location-years. Data were excluded if they violated well-established age or time trends, and data in instances where garbage code redistribution and noise reduction, in combination with small sample sizes, resulted in unreasonable cause fractions. Methods for assigning outlier status were consistent across both vital registration and verbal autopsy data.

4.3 Modelling

To estimate appendicitis mortality, a standard causes of death ensemble model (CODEm) with location-level covariates was used.[4,6] CODEm, which uses an ensemble modelling method that involves generation and validation of submodels using the train-test 1-test 2 approach, weighting and testing of the model performance to select the best ensemble model with the highest out-of-sample predictive validity, has originally been described in a peer-reviewed publication by Foreman et al.[6] The updates to the method have been described in a series of publications reporting GBD cause of death results.[4,7–10]

As described in greater detail in Foreman et al, CODEm modelling starts with generation of a diverse family of potential submodels (i.e., component models) in four families: linear mixed effects regression (LMER) models of the natural log of the cause-specific death rate, LMER models of the logit of the cause fraction, spatiotemporal Gaussian process regression (ST-GPR) models of the natural logarithm of the

cause-specific death rate, and ST-GPR models of the logit of the cause fraction. In any given application, the analyst specifies all plausible covariates associated with a particular cause of death. For each covariate identified, the analyst specifies the expected direction (positive or negative) of its relationship to cause of death based on scientific evidence and classifies it into one of the three levels (Levels 1, 2, and 3) based on the plausibility and strength of evidence for causal association. The level of each indicator represents the level of association between the covariate and appendicitis mortality. It ranges from 1, indicating a strong biological link to outcome, to 3, indicating weak or unknown relationship to outcome. The direction of each indicator represents the expected direction of relationship to cause of death.

For appendicitis, we included six predictive covariates, as shown in Table S4, which were modelled and estimated as part of GBD. Details of each covariate's input data and modelling strategies are described elsewhere.[3,11–13]

Table S4. Covariates used in appendicitis mortality modelling

Level	Covariate	Direction
2	Age-sex-specific scaled exposure variable for low fruit consumption	+
	Age-sex-specific scaled exposure variable for low vegetable consumption	+
	Healthcare Access and Quality Index	-
3	Socio-demographic Index	-
	Education (years per capita)	-
	Log LDI ($I per capita)	-

Combinations of these covariates are tested for performance in submodels. The linear mixed effects submodels include fixed effects on covariates and age group and nested random effects on super-region, region, country, and age, and can be represented by:

$$ln(rate_{s,r,c,y,a}) = \sum_i \beta_i X_{i_{s,r,c,y,a}} + \beta_a + \pi_s + \pi_{s,r} + \pi_{s,r,a} + \pi_{s,r,a,c} + \varepsilon_{s,r,c,y,a}$$

$$logit(cause\ fraction_{s,r,c,y,a}) = \sum_i \beta_i X_{i_{s,r,c,y,a}} + \beta_a + \pi_s + \pi_{s,r} + \pi_{s,r,a} + \pi_{s,r,a,c} + \varepsilon_{s,r,c,y,a}$$

Where:
s = super-region index; r = region index; c = country index; y = year index; a = age index; [countries are nested within regions, which are nested within super-regions
β_i = coefficient on covariate i
$X_{i_{s,r,c,y,a}}$ = covariate i for observation s, r, c, y, a
β_a = age-specific intercept
π_s = random intercept on super-region
$\pi_{s,r}$ = random intercept on region (nested within super-region)
$\pi_{s,r,a}$ = random intercept on age (nested within region)
$\pi_{s,r,a,c}$ = random intercept on country (nested within region-age)
$\varepsilon_{s,r,c,y,a} \sim N(0, \sigma_\varepsilon \cdot I)$

The ST-GPR models begin with a linear prediction, per above, and then smooth over space, time, and age based on weighted residuals of adjacent data and then apply Gaussian process regression.

The process of covariate selection proceeds by level. We test submodels corresponding to all 2*n* - 1 combinations of level 1 covariates, where *n* is the number of covariates in level 1. All submodels where the coefficients for all covariates have the expected sign and are significant at the $p < 0.05$ level are retained. For each level 1 model that was retained, we create a list of 2*m* possible level 2 models (where *m* is the number of level 2 covariates). The first model, which has no level 2 covariates included, has already been tested and is retained. Next, each of the *m* possible models in which one covariate is added to the level 1 model is tested. If adding the level 2 covariate does not affect either the significance or the sign on any level 1 coefficients, and the level 2 covariate itself meets the criteria of direction and significance, then it is retained as another possible submodel. If the level 2 covariate does not fulfill the criteria or forces any of the level 1 covariates to violate their criteria, then the submodel is dropped; all other possible level 2 submodels that contain that covariate are also dropped. Next, we take each of the models resulting from the level 2 process and use the same process as described for level 2 on the level 3 covariates. Ultimately, we obtain a set of all possible covariate combinations that fulfill our expectations for covariate direction. We run the covariate selection for submodels using both logit cause fraction and natural log of cause-specific death rate and then create both LMER-only and ST-GPR models for each set of chosen covariates.

Submodels are fit on 70% of CoD data, with 30% holdouts of the data selected to mimic observed patterns of missingness for age groups, years, and locations. Out-of-sample predictive validity of each submodel is then tested using half of the excluded data (15% of the total). Performance tests include the root-mean-squared-error (RMSE) for the log of the cause-specific death rate, the direction of the predicted versus actual trend in the data, and the coverage of the predicted 95% uncertainty interval. The process of fitting submodels and calculating out-of-sample performance is repeated 20 times. Submodels are ranked according to their performance on these out-of-sample performance tests across the 20 repetitions. Submodels are then weighted to determine their contribution to the ensemble estimate. The relative weights are determined both by the submodel performance ranks and by a parameter ψ, whose value determines how quickly the weights taper off as rank decreases. Specifically, the relationship between weight and parameter ψ is specified using a monotonically decreasing function, given by,

$$weight_i = \frac{\psi^{N-rank_i}}{\sum_{j=1}^{N} \psi^{N-j}}$$

The analyst may specify the range of ψ values (usually between 1 and 1.2, as is true for the appendicitis model) to test in order to identify the best ψ value based in the cross-validation.

A set of ensemble models is then created by using the weights constructed from the combinations of ranks and ψ values. These ensembles are tested by using the predictive validity metrics described above on the remaining 15% of the data, and the ensemble with the best performance in out-of-sample trend and RMSE is chosen as the final model.

There are several model parameters that the analyst can set in CODEm, including the size of the data hold-outs, the maximum and minimum values for ψ, the linear floor rate (the lowest death rate per 100,000 in the linear predictor), as well as lambda, zeta, omega and GPR parameters. For appendicitis, we used the standard default settings for GBD.[10]

The entire ensemble modelling approach is conducted twice for GBD: once to fit a global CODEm model that uses data from, and makes predictions for, all locations in the GBD estimation framework; and once to fit a data-rich CODEm model, which uses data from, and makes predictions for, only the locations considered to be data-rich. The designation of data-rich locations is based on a "star" rating system (0–5 stars) to rate the quality of data for any given location and year, with 5 being the best and 0 being the

worst. The inputs that determine this star rating are the percentage of total deaths determined to be garbage coded (such as "All, Ill-defined"), the percentage of deaths determined to be an aggregated cause, and the level of completeness in the dataset. More detailed information about the causes of death data star rating calculation can be found on pages 45-48 of the Supplementary document of the GBD 2019 publication.[4]

We assumed that children under 1 year of age do not die from appendicitis; we kept the age restrictions for mortality estimation of appendicitis of 12 months for lower bound and 95+ years for upper bound. Separate models were conducted for male and female mortality in addition to the global and data-rich locations as mentioned above. We hybridised separate sex-specific global and data-rich models to acquire unadjusted results, which we adjusted using the cause of death correction (CoDCorrect) procedure[4] and compared to the reference life table to calculate final YLLs due to appendicitis.

4.4 Summary statistical metrics on model performance

The table below shows the overall predictive performance of sex-specific global and data-rich models of appendicitis.

Table S5. Summary statistical metrics on ensemble model performance of appendicitis

	Out-of-sample RMSE	Coverage	Test of trends	Mean error
Male, global	0.4102	0.9966	0.1547	6.6610
Male, data-rich	0.2735	1.000	0.1322	0.0159
Female, global	0.4647	0.9966	0.1702	6.0601
Female, data-rich	0.3030	0.9998	0.1787	0.0574

Section 5. Non-fatal estimation

5.1 Flowchart

The flowchart below describes the incidence, YLD, and DALY estimation processes used for appendicitis in the Global Burden of Disease (GBD) study.

Figure S3. Flow diagram of non-fatal health outcome estimation for appendicitis

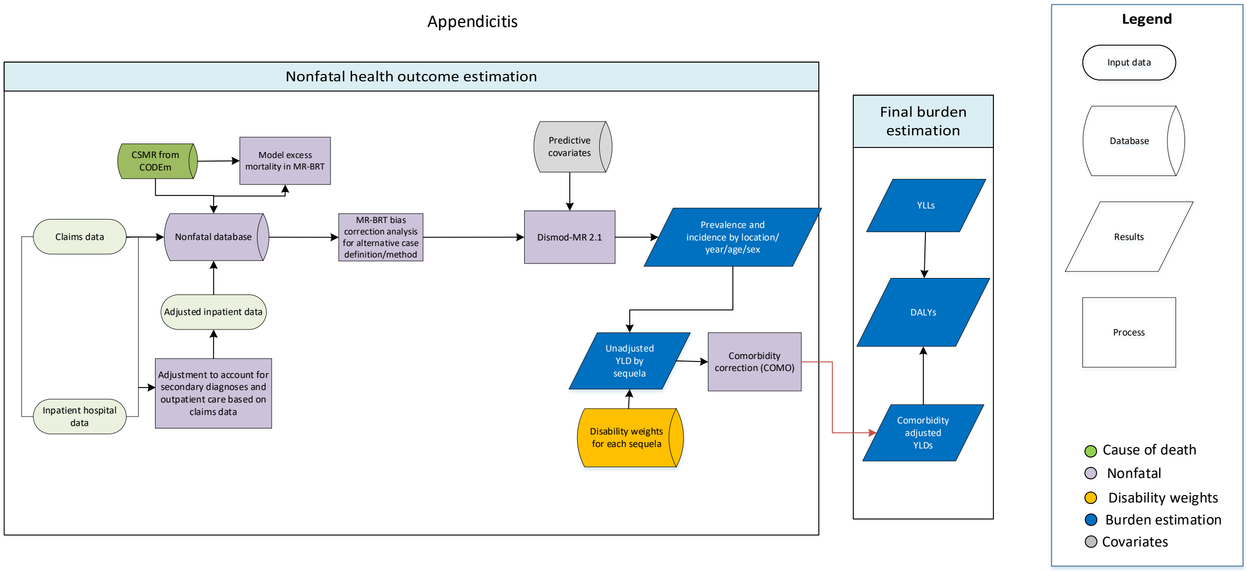

5.2 Data identification and processing

5.2.1 Incidence data

We used medical claims and hospital discharge data to model appendicitis incidence. Medical claims data come from three locations: USA, Poland, and Taiwan (province of China). The USA claims data were extracted from the Truven database of USA private health insurance, which contains more than 12 billion claims in 2000, and 2010–2017. Both Poland and Taiwan medical insurance claims data were extracted from the national insurance programs, which have over 90% of population coverage.[14–16] Hospital inpatient discharge data were extracted from 95 different sources in 50 countries, each covering one to 26 years of data from 1988. Figure S4 shows the data coverage map of these input sources.

Figure S4. Data coverage for estimating incidence of appendicitis

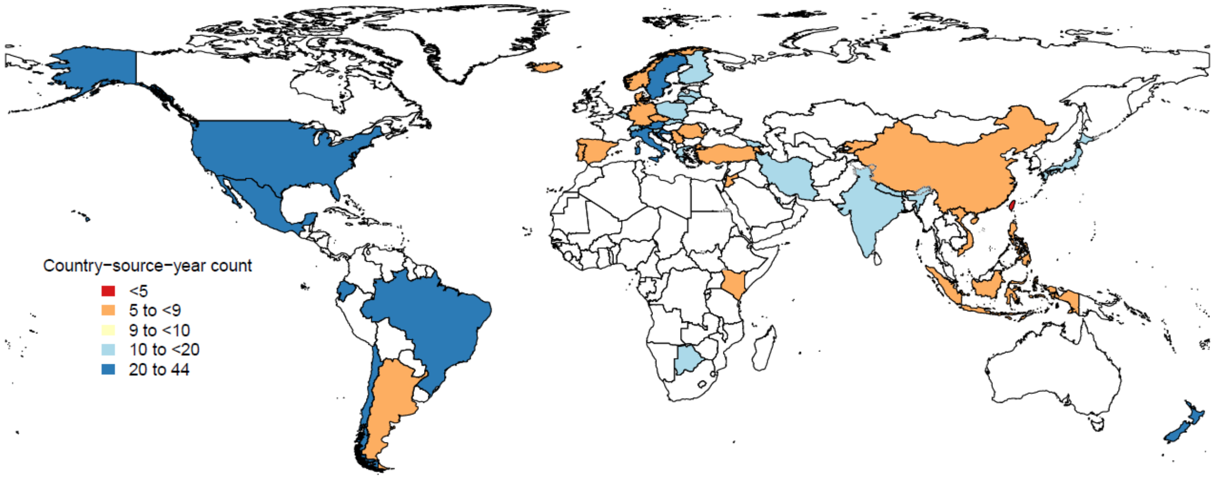

The biggest advantage of the three claims data sources we have is being able to link claims for all inpatient and outpatient encounters for a single individual using the unique identifier IDs (de-identified), regardless of whether appendicitis is coded as primary or secondary diagnosis. For appendicitis specifically, to capture cases that were diagnosed and/or treated in both inpatient and outpatient settings, an individual was extracted from claims data as an incident case if that individual had at least one inpatient or outpatient encounter with an appropriate ICD code related to appendicitis (Table S5) as any diagnosis within 28 days. The population sample from which those cases arose was considered to comprise all individuals enrolled in that insurance plan that calendar year.

Hospital discharge data provide observations about encounters, generally with only the primary diagnostic code for the encounter. For these data sources, we applied the inclusion criteria that an admission is an overnight stay of 24 hours or more; discharges with a length of stay (LOS) less than 24 hours are excluded from all inpatient sources wherever possible. This was done for databases known to include "hospital outpatient" and "observation" admissions, because several hospital databases in our dataset are known or suspected not to include these shorter encounters, and we wanted to construct like databases for comparison. For Truven's MarketScan database, we do not apply the same filtering for LOS <1 as in inpatient data sources because the MarketScan claims data are already organised in a way that separates hospital outpatient data from inpatient admissions – the hospital outpatient claims in the MarketScan database are included in the "outpatient services" file and are used for outpatient claims

estimates. In addition, in the MarketScan database, a claim must also meet certain inclusion criteria to be considered an inpatient admission, such as having a room and board claim. If these criteria are not met, the records are stored in the Outpatient Services Table and no admission record is created.

Hospital inpatient discharge data were adjusted to account for diagnosis and treatment across all admission types and levels of care through multiple data processing steps. Specifically, for each source, we calculated the fraction of all hospital admissions primarily due to appendicitis for a given year, age group, and sex. This fraction was then multiplied by the annual hospital utilisation rate for that specific year, age group, and sex to estimate the annual rates of primary appendicitis admission. To calculate the annual population estimates of appendicitis cases for a given year, age group, and sex, these annual rates of primary admission of appendicitis were adjusted using correction factors that were modelled from the ratio of inpatient discharge with appendicitis as primary diagnosis to all cases ascertained in any health care encounter (inpatient versus outpatient claims data, primary versus secondary diagnosis), using MarketScan claims data as a detailed source of data for all health care encounters for an individual for a year, and then applying that ratio to all inpatient discharge data for all locations. This makes the assumption that in other populations, those cases that either receive care outside of an inpatient encounter (with more than 24 hours of overnight stay) or never receive care at all (and thus are missing from our inpatient data for those populations) occur in approximately the same ratio to the inpatient encounters as in the MarketScan database. Thus, the ratio inflates the inpatient discharge data to account for those missing cases. Estimating the correction factors is done using a non-linear mixed-effects meta-regression tool called meta-regression—Bayesian, regularised, trimmed (MR-BRT). The details about the MR-BRT tool have been published previously.[4,17] Briefly, the MR-BRT program is a set of wrappers customised for global health problems that use the open-source mixed effects package limeTr (https://github.com/zhengp0/limetr). Specifically, the logs of these ratios were calculated and used as an input to MR-BRT using covariates for age and sex. The equation used for correction factor estimation is shown below (correction to account for inpatient and outpatient care, which provides inpatient admissions and outpatient visits by individuals for all diagnoses):

$$inpatient^{1°}_{admission} * \left(\frac{inpatient^{all}_{indiv} \cup outpatient^{all}_{indiv}}{inpatient^{1°}_{admissions}}\right) = inpatient|outpatient^{all}_{indiv}$$

Table S6. International Classification of Diseases (ICD) codes mapped to Global Burden of Disease cause list for appendicitis incidence data

ICD system	ICD codes
10	K35 (K35, K35.0, K35.1, K35.2, K35.3, K35.8, K35.80, K35.89, K35.9)<br>K36 (K36, K36.0)<br>K37 (K37, K37.0, K37.9)
9	540 (540, 540.0, 540.1, 540.9)<br>541 (541, 541.0, 541.1, 541.2, 541.3, 541.9)<br>542 (542, 542.0, 542.1, 542.9)

In our model, the reference standard for incidence input data was a combination of adjusted inpatient data and claims data from Poland and Taiwan. This reference serves as a benchmark for adjusting other data sources that are known to have a systematic difference. For appendicitis, specifically in the USA, we used two major sources of data: Truven MarketScan claims data and the Healthcare Cost and Utilization Project, or HCUP. HCUP is considered a highly reliable source of information about disease prevalence and health care utilisation. Our database includes HCUP data for 13 USA states. To produce estimates for all 50 states using available data and correcting for errors, we augmented the database with data from

Truven, which were available for all states. To ensure comparability and generalisability of the Truven database, adjusted data from USA MarketScan claims towards the reference (HCUP) to account for systematic differences in its incidence measure due to commercial insurance status in the enrollees. The process of adjusting for biases in non-reference data using MR-BRT with the logit-transformation method is described below:

1. Identify datapoints with overlapping year, age, sex, and location between commercial claims (non-reference data) and population-representative hospital discharges (reference data).
2. Logit transform overlapping datapoints of alternative and reference types.
3. Convert overlapping datapoints into a difference in logit space using the following equation: $logit(alternative) - logit(reference)$.
4. Use the delta method to compute standard errors of overlapping datapoints in logit space, then calculate standard error of logit difference using the following equation: $\sqrt{(variance\ of\ logit(alternative)) + (variance\ of\ logit(reference))}$
5. Using MR-BRT, conduct a random effects meta-regression to obtain the pooled logit difference of alternative to reference.
6. Apply the pooled logit difference to all datapoints of alternative case definitions using the following equation:
$$new_{estimate} = inverse.logit((logit(alternative)) - (pooled\ logit\ difference))$$
7. Calculate new standard errors using the delta method, accounting for gamma (between-study heterogeneity).

The table below shows bias correction factors estimated using MR-BRT.

Table S7. MR-BRT crosswalk adjustment factors for appendicitis

Data input	Reference or alternative data collection	Gamma	Covariate	Beta coefficient, logit (95% uncertainty interval)	Adjustment factor*
Hospital + non-USA claims	Ref	0.002		---	---
USA claims from year 2000	Alt		Age (continuous from 0 to 95+ years)	0.01 (–0.08 to 0.10)	1.01 (0.92 to 1.10)
			Sex (female to male)	0.11 (–0.07 to 0.28)	1.11 (0.94 to 1.33)
			Intercept	–0.76 (–1.01 to –0.52)	0.47 (0.37 to 0.60)
USA claims from 2010–2017	Alt		Age (continuous from 0 to 95+ years)	0.004 (–0.06 to 0.07)	1.00 (0.94 to 1.08)
			Sex (female to male)	0.14 (–0.30 to 0.58)	1.15 (0.74 to 1.78)
			Intercept	–0.825 (–1.40 to –0.25)	0.44 (0.25 to 0.78)

**MR-BRT crosswalk adjustments can be interpreted as the factor the alternative case definition is adjusted by to reflect what it would have been had it been measured using the reference case definition. If the log/logit beta coefficient is negative, then the alternative is adjusted up to the reference. If the log/logit beta coefficient is positive, then the alternative is adjusted down to the reference.*
***The adjustment factor column is the exponentiated beta coefficient. For log beta coefficients, this is the relative rate between the two case definitions. For logit beta coefficients, this is the relative odds between the two case definitions.*

To address the most substantial sources of heterogeneity, datapoints with an age-standardised incidence rate greater than two median absolute deviations from the median of the age-standardised incidence rate for all data were marked as outliers and excluded from analysis.

5.2.2 Excess mortality estimates

Excess mortality rate (EMR) is defined as the number of deaths due to appendicitis among those who have appendicitis. In previous rounds of GBD, EMR inputs were produced by matching prevalence datapoints with their corresponding CSMR values within the same age, sex, year, and location (by dividing CSMR by prevalence). For short-duration conditions (remission >1), the corresponding prevalence was derived by running an initial model and then applying the same CSMR/prevalence method. However, this method of producing EMR inputs demonstrated a rather unrealistic pattern of EMR compared to an expected pattern of decreasing EMR with greater access to quality health care. Such unexpected patterns often signal inconsistencies between CSMR estimates and the measures of prevalence and/or incidence. Thus, in an effort to provide greater guidance on the expected pattern of EMR, EMR data produced per above were modelled by age, sex and Healthcare Access and Quality (HAQ) Index using MR-BRT, with a prior on HAQ Index having a negative coefficient. The equation used for this regression is shown below.

$$\log(EMR) = \beta_0 + \beta_1 sex + \beta_2 HAQ + f(age) + u_{loc-study} + \epsilon_i$$

EMR predictions for each location, year, sex, and for ages 0, 10, 20….100 were used as inputs to our non-fatal model. Details on this new modelling method for EMR inputs used for DisMod is described on pages 465-6 of Appendix 1 of the GBD 2019 Diseases and Injuries Capstone.[4]

5.2.3 Cause-specific mortality rates

Cause-specific mortality rate (CSMR) estimates drawn from the fatal estimation process described above in Section 2 were used in estimating incidence.

5.3 Modelling

To estimate incidence of appendicitis, a standard DisMod-MR 2.1 model with location-level covariates was used. Details on DisMod have been published elsewhere.[4,18–20] Briefly, DisMod is a Bayesian mixed-effects meta-regression tool that uses a Bayesian compartmental model framework that solves differential equations for incidence, remission, mortality, and the resulting balance of prevalent cases. It modulates the relationships between a susceptible population, prevalent cases, and those who remit or die from the disease. DisMod solves these differential equations repeatedly down a geographical cascade, starting with global, then down to seven super-regions, 21 regions, 204 countries and territories, and subnational locations for some countries. At the global level, DisMod uses all available data to fit sex-specific estimates for all epidemiological parameters in a steady-state compartmental model with mixed-effects, non-linear regression. The model fit at the global stage is then combined with fixed effects from predictive covariates and location random effects and used as a Bayesian prior for seven models specific to each of the seven super-regions. This process repeats all the way down to the most detailed locations estimated in GBD. The final age-, sex-, location-, and year-specific burden estimates for each epidemiological parameter are produced by aggregating the estimates of disease frequency in the finest locations back up the geographical cascade.

For appendicitis, data inputs for DisMod-MR 2.1 included incidence data from the clinical administrative sources, cause-specific mortality rates estimated as described in Section 4 of this appendix, and excess mortality rates that were separately estimated using the Healthcare Access and Quality Index. A prior value was set on remission so that all cases remit within two weeks. The minimum coefficient of variation

at the regional, super-regional, and global level was set at 0.8. We included HAQ Index as a predictive covariate on EMR with a mean and standard deviation produced from the MR-BRT model described above. The fibre (g per day) consumption covariate was included as a predictive covariate on incidence. Betas and exponentiated values (which can be interpreted as odds ratios) of predictive covariates are shown in the table below.

Table S8. Summary of covariates used in the appendicitis DisMod-MR meta-regression model

Covariate	Parameter	Exponentiated beta (95% uncertainty interval)
Fibre unadjusted (g)	Incidence	0.99 (0.99 – 1.00)
Healthcare Access and Quality Index	Excess mortality rate	0.95 (0.95 – 0.95)

5.4 Summary statistical metrics on model performance

Table below shows the overall predictive performance of DisMod model of appendicitis.

Table S9. Summary statistical metrics on DisMod model performance of appendicitis

Measure	Type	Value
Incidence hazard	In-sample RMSE	0.6129972082301519
Incidence hazard	In-sample coverage (by integrand)	0.6713140355657751
Excess mortality rate	In-sample RMSE	0.7543284461022027
Excess mortality rate	In-sample coverage (by integrand)	0.7087152623459834
Cause-specific mortality rate	In-sample RMSE	1.2788348230100823
Cause-specific mortality rate	In-sample coverage (by integrand)	0.9825706039991754
All-cause mortality rate	In-sample RMSE	0.26528311175285146
All-cause mortality rate	In-sample coverage (by integrand)	1

5.5 Disability weight

GBD estimation of disability weights for estimation of years lived with disability (YLDs) has been previously described.[4,7,21,22] Briefly, disability weight represents the magnitude of health loss associated with the disease outcome. It ranges from 0 (perfect health) to 1 (death). Disability weights used for GBD are derived from a series of household and web-based surveys that were conducted between 2009 and 2013 in nine countries. The surveys included a series of lay descriptions of two hypothetical people with two distinct health states, and asked the survey respondents to choose which of the two was healthier. This pair-wise comparisons were analysed using probit regression analyses to infer distances between disability weight values for pairs of states, anchoring to a 0–1 scale. For appendicitis, given the acute nature of the disease, we assumed all incident cases experienced a single health state (severe abdominal pain). The health state, lay description, and disability weight used for appendicitis are shown in Table S10.

Table S10. Disability weight and health state of appendicitis

Health state	Lay description	Disability weight (95% uncertainty interval)
Severe abdominal pain	This person has severe pain in the belly and feels nauseated. The person is anxious and unable to carry out daily activities.	0.324 (0.219 – 0.442)

Section 6. Additional Figures

Figure S5. Global temporal trend of years of life lost (YLLs) of appendicitis between 1990 and 2021

The black line represents the total YLL counts in millions with the grey shade representing the 95% uncertainty intervals. The red line represents the total YLL counts in millions with the light red shade representing the 95% uncertainty intervals.

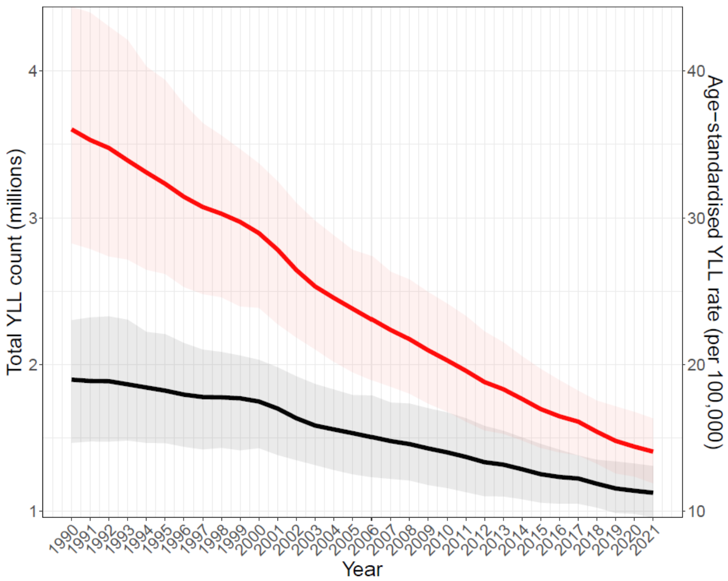

Figure S6. Global temporal trend of years lived with disability (YLDs) of appendicitis between 1990 and 2021

The black line represents the total YLD counts in millions with the grey shade representing the 95% uncertainty intervals. The red line represents the total YLD counts in millions with the light red shade representing the 95% uncertainty intervals.

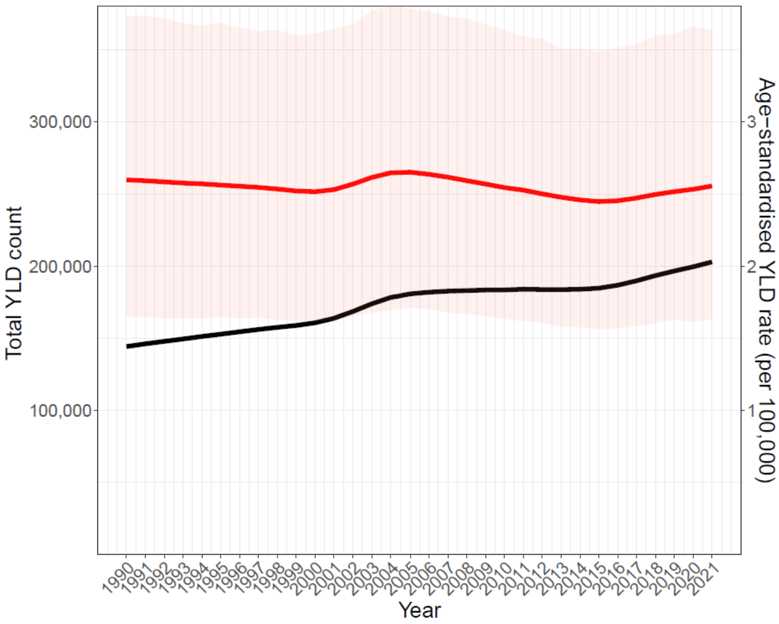

References

1 Omar B. Ahmad, Cynthia Boschi-Pinto, Alan D. Lopez, Christopher J.L. Murray, Rafael Lozano, Mie Inoue. Age standardization of rates: A new WHO standard. 2001. https://www.who.int/healthinfo/paper31.pdf (accessed Aug 17, 2019).

2 GBD 2021 Demographics Collaborators. Global age-sex-specific mortality, life expectancy, and population estimates in 204 countries and 811 subnational locations, 1950-2021: a comprehensive demographic analysis for the Global Burden of Disease Study 2021. *Lancet* (in drafting).

3 GBD 2019 Demographics Collaborators. Global age-sex-specific fertility, mortality, healthy life expectancy (HALE), and population estimates in 204 countries and territories, 1950-2019: a comprehensive demographic analysis for the Global Burden of Disease Study 2019. *Lancet* 2020; **396**: 1160–203.

4 GBD 2019 Diseases and Injuries Collaborators. Global burden of 369 diseases and injuries in 204 countries and territories, 1990-2019: a systematic analysis for the Global Burden of Disease Study 2019. *Lancet* 2020; **396**: 1204–22.

5 Naghavi M, Makela S, Foreman K, O'Brien J, Pourmalek F, Lozano R. Algorithms for enhancing public health utility of national causes-of-death data. *Population Health Metrics* 2010; **8**: 9.

6 Foreman KJ, Lozano R, Lopez AD, Murray CJ. Modeling causes of death: an integrated approach using CODEm. *Population Health Metrics* 2012; **10**: 1.

7 GBD 2016 Disease and Injury Incidence and Prevalence Collaborators. Global, regional, and national incidence, prevalence, and years lived with disability for 328 diseases and injuries for 195 countries, 1990-2016: a systematic analysis for the Global Burden of Disease Study 2016. *Lancet* 2017; **390**: 1211–59.

8 GBD 2017 Causes of Death Collaborators. Global, regional, and national age-sex-specific mortality for 282 causes of death in 195 countries and territories, 1980-2017: a systematic analysis for the Global Burden of Disease Study 2017. *Lancet* 2018; **392**: 1736–88.

9 GBD 2013 Mortality and Causes of Death Collaborators. Global, regional, and national age-sex specific all-cause and cause-specific mortality for 240 causes of death, 1990-2013: a systematic analysis for the Global Burden of Disease Study 2013. *Lancet* 2015; **385**: 117–71.

10 GBD 2021 Diseases and Injuries Collaborators. Global incidence, prevalence, years of life lived with disability (YLDs) and disability-adjusted life-years (DALYs), and healthy life expectancy (HALE) for 371 diseases and injuries in 204 countries and 811 subnational locations, 1990-2021: a systematic analysis for the Global Burden of Disease Study 2021. *Lancet* (in drafting).

11 Murray CJL, Aravkin AY, Zheng P, *et al.* Global burden of 87 risk factors in 204 countries and territories, 1990–2019: a systematic analysis for the Global Burden of Disease Study 2019. *The Lancet* 2020; **396**: 1223–49.

12 Friedman J, York H, Graetz N, *et al.* Measuring and forecasting progress towards the education-related SDG targets. *Nature* 2020; **580**: 636–9.

13 GBD 2015 Healthcare Access and Quality Collaborators. Healthcare Access and Quality Index based on mortality from causes amenable to personal health care in 195 countries and territories, 1990-2015: a novel analysis from the Global Burden of Disease Study 2015. *Lancet* 2017; **390**: 231–66.

14 Wu T-Y, Majeed A, Kuo KN. An overview of the healthcare system in Taiwan. *London J Prim Care (Abingdon)* 2010; **3**: 115–9.

15 衛生福利部中央健康保險署. National Health Insurance Administration Ministry of Health and Welfare-Unversal Health Coverage in Taiwan. 衛生福利部中央健康保險署. 2016; published online Oct 1. https://www.nhi.gov.tw/English/Content_List.aspx?n=4D7051840BF42F52&topn=ED4A30E51A609E49&Create=1 (accessed Nov 6, 2020).

16 Poland: Country Health Profile 2017 | en | OECD. https://www.oecd.org/poland/poland-country-health-profile-2017-9789264283510-en.htm (accessed Nov 6, 2020).

17 Zheng P, Barber R, Sorensen RJD, Murray CJL, Aravkin AY. Trimmed Constrained Mixed Effects Models: Formulations and Algorithms. *Journal of Computational and Graphical Statistics* 2021; **0**: 1–13.

18 Barendregt JJ, Van Oortmarssen GJ, Vos T, Murray CJ. A generic model for the assessment of disease epidemiology: the computational basis of DisMod II. *Popul Health Metr* 2003; **1**: 4.

19 Peterson HM, Flaxman AD. Meta-regression with DisMod-MR: how robust is the model? *The Lancet* 2013; **381**: S110.

20 Abraham D. Flaxman, Theo Vos, and Christopher J. L. Murray, editor. An Integrative Metaregression Framework for Descriptive Epidemiology. Seattle: University of Washington Press, 2015.

21 Salomon JA, Haagsma JA, Davis A, *et al.* Disability weights for the Global Burden of Disease 2013 study. *Lancet Glob Health* 2015; **3**: e712-723.

22 Haagsma JA, Maertens de Noordhout C, Polinder S, *et al.* Assessing disability weights based on the responses of 30,660 people from four European countries. *Popul Health Metr* 2015; **13**: 10.

Appendix 3: Authorship appendix to "Global, regional, national burden of appendicitis from 1990-2021: an analysis for the Global Burden of Disease 2021"

This appendix provides further authorship detail for "Global, regional, national burden of appendicitis from 1990-2021: an analysis for the Global Burden of Disease 2021."

Table of Contents

GBD 2021 Appendicitis Collaborators....18
Affiliations....19
Authors' Contributions....23
Managing the overall research enterprise....23
Writing the first draft of the manuscript....23
Primary responsibility for applying analytical methods to produce estimates....23
Primary responsibility for seeking, cataloguing, extracting, or cleaning data; designing or coding figures and tables....23
Providing data or critical feedback on data sources....23
Developing methods or computational machinery....24
Providing critical feedback on methods or results....24
Drafting the work or revising it critically for important intellectual content....24
Managing the estimation or publications process....25

GBD 2021 Appendicitis Collaborators

Hannah Han*, Ian D Letourneau, Yohannes Habtegiorgis Abate, Michael Abdelmasseh, Eman Abu-Gharbieh, Tigist Demssew Adane, Bright Opoku Ahinkorah, Aqeel Ahmad, Ali Ahmadi, Ayman Ahmed, Fadwa Naji Alhalaiqa, Salman Khalifah Al-Sabah, Yaser Mohammed Al-Worafi, Hubert Amu, Catalina Liliana Andrei, Amir Anoushiravani, Jalal Arabloo, Aleksandr Y Aravkin, Tahira Ashraf, Sina Azadnajafabad, Nayereh Baghcheghi, Sara Bagherieh, Berihun Bantie Bantie, Mainak Bardhan, Guido Basile, Nebiyou Simegnew Bayleyegn, Amir Hossein Behnoush, Alehegn Bekele, Vijayalakshmi S Bhojaraja, Ali Bijani, Antonio Biondi, Katrin Burkart, Dinh-Toi Chu, Isaac Sunday Chukwu, Natalia Cruz-Martins, Xiaochen Dai, Berecha Hundessa Demessa, Arkadeep Dhali, Daniel Diaz, Thanh Chi Do, Milad Dodangeh, Deepa Dongarwar, Haneil Larson Dsouza, Michael Ekholuenetale, Temitope Cyrus Ekundayo, Iman El Sayed, Muhammed Elhadi, Adeniyi Francis Fagbamigbe, Ildar Ravisovich Fakhradiyev, Pietro Ferrara, Getahun Fetensa, Florian Fischer, Mesfin Gebrehiwot, Melaku Getachew, Mahaveer Golechha, Vivek Kumar Gupta, Joseph R Habib, Najah R Hadi, Nils Haep, Teklehaimanot Gereziher Haile, Erin B Hamilton, Ikramul Hasan, Hamidreza Hasani, Sara Hassanzadeh, Johannes Haubold, Simon I Hay, Khezar Hayat, Olayinka Stephen Ilesanmi, Sumant Inamdar, Chidozie C D Iwu, Assefa N Iyasu, Umesh Jayarajah, Shubha Jayaram, Mohammad Jokar, Nabi Jomehzadeh, Abel Joseph, Nitin Joseph, Charity Ehimwenma Joshua, Ali Kabir, Himal Kandel, Joonas H Kauppila, Phillip M. Kemp Bohan, Himanshu Khajuria, Maseer Khan, Haitham Khatatbeh, Min Seo Kim, Adnan Kisa, Farzad Kompani, Hamid Reza Koohestani, Rakesh Kumar, Thao Thi Thu Le, Munjae Lee, Seung Won Lee, Ming-Chieh Li, Stephen S Lim, Chun-Han Lo, Raimundas Lunevicius, Kashish Malhotra, Andrea

Maugeri, Rishi P Mediratta, Tuomo J Meretoja, Tomislav Mestrovic, Mohammad Mirza-Aghazadeh-Attari, Nouh Saad Mohamed, Ali H Mokdad, Lorenzo Monasta, Mohammad Ali Moni, Maryam Moradi, Vincent Mougin, George Duke Mukoro, Efren Murillo-Zamora, Christopher J L Murray, Mukhammad David Naimzada, Hastyar Hama Rashid Najmuldeen, Zuhair S Natto, Ionut Negoi, Hien Quang Nguyen, Taxiarchis Konstantinos Nikolouzakis, Isaac Iyinoluwa Olufadewa, Jagadish Rao Padubidri, Ashok Pandey, Romil R Parikh, Hoang Tran Pham, Richard Charles G Pollok, Mehran Rahimi, Vafa Rahimi-Movaghar, Mosiur Rahman, Shayan Rahmani, Mohammad-Mahdi Rashidi, Salman Rawaf, Jennifer Rickard, Hamidreza Rouientan, Simanta Roy, Basema Ahmad Saddik, Umar Saeed, Mohamed A Saleh, Sana Salehi, Abdallah M Samy, Juan Sanabria, Senthilkumar Sankararaman, Austin E Schumacher, Subramanian Senthilkumaran, Pritik A Shah, Sina Shool, Migbar Mekonnen Sibhat, Negussie Boti Sidamo, Jasvinder A Singh, Bogdan Socea, Yonatan Solomon, Saraswathy Sreeram, Seyyed Mohammad Tabatabaei, Ker-Kan Tan, Seyed Mohammad Tavangar, Yibekal Manaye Tefera, Nikhil Kenny Thomas, Jansje Henny Vera Ticoalu, Guesh Mebrahtom Tsegay, Dejen Tsegaye, Sana Ullah, Abachebissa Nuru Usman, Rohollah Valizadeh, Massimiliano Veroux, Georgios-Ioannis Verras, Theo Vos, Mei Wang, Song Wang, Dakshitha Praneeth Wickramasinghe, Galal Yahya, Iman Zare, Armin Zarrintan, Zhi-Jiang Zhang, and M Ashworth Dirac**.

* Lead Author
** Senior Author

Affiliations

Institute for Health Metrics and Evaluation (H Han MSc, I D Letourneau BA, A Y Aravkin PhD, K Burkart PhD, X Dai PhD, E B Hamilton MPH, Prof S I Hay FMedSci, Prof S S Lim PhD, T Mestrovic PhD, A H Mokdad PhD, V Mougin BA, Prof C J L Murray DPhil, A E Schumacher PhD, Prof T Vos PhD, M Wang MPH, M A Dirac MD), Department of Applied Mathematics (A Y Aravkin PhD), Department of Health Metrics Sciences, School of Medicine (A Y Aravkin PhD, K Burkart PhD, X Dai PhD, Prof S I Hay FMedSci, Prof S S Lim PhD, A H Mokdad PhD, Prof C J L Murray DPhil, Prof T Vos PhD, M A Dirac MD), Department of Family Medicine (M A Dirac MD), University of Washington, Seattle, WA, USA; Department of Clinical Governance and Quality Improvement (Y H Abate MSc), Aleta Wondo Hospital, Aleta Wondo, Ethiopia; Department of Surgery (M Abdelmasseh MD, Prof J Sanabria MD), Marshall University, Huntington, WV, USA; Clinical Sciences Department (Prof E Abu-Gharbieh PhD), College of Medicine (Prof B A Saddik PhD, Prof M A Saleh PhD), University of Sharjah, Sharjah, United Arab Emirates; Department of Biopharmaceutics and Clinical Pharmacy (Prof E Abu-Gharbieh PhD), The University of Jordan, Amman, Jordan; Department of Clinical and Psychosocial Epidemiology (T D Adane MSc), University of Groningen, Groningen, Netherlands; School of Public Health (B O Ahinkorah MPhil), University of Technology Sydney, Sydney, NSW, Australia; Department of Medical Biochemistry (A Ahmad PhD), Shaqra University, Shaqra, Saudi Arabia; Department of Epidemiology and Biostatistics (A Ahmadi PhD), Shahrekord University of Medical Sciences, Shahrekord, Iran; Department of Epidemiology (A Ahmadi PhD), School of Medicine (S Rahmani MD), Social Determinants of Health Research Center (M Rashidi MD), Urology and Nephrology Research Center (H Rouientan MD), Department of Surgery (H Rouientan MD), Emergency Department (S Shool MD), Shahid Beheshti University of Medical Sciences, Tehran, Iran; Institute of Endemic Diseases (A Ahmed MSc), University of Khartoum, Khartoum, Sudan; Swiss Tropical and Public Health Institute (A Ahmed MSc), University of Basel, Basel, Switzerland; College of Nursing (Prof F A N Alhalaiqa PhD), Qatar University, Doha, Qatar; Psychological Sciences Association, Amman, Jordan (Prof F A N Alhalaiqa PhD); Department of Surgery (S K Al-Sabah MD), Kuwait University, Kuwait, Kuwait; Jaber Al Ahmad Al Sabah Hospital (S K Al-Sabah MD), Ministry of Health, Kuwait, Kuwait; Department of Medical Sciences (Prof Y M Al-Worafi PhD), Azal University for Human Development, Sana'a, Yemen; Department of Clinical Sciences (Prof Y M Al-Worafi PhD), University of Science and Technology of

Fujairah, Fujairah, United Arab Emirates; Department of Population and Behavioural Sciences (H Amu PhD), University of Health and Allied Sciences, Ho, Ghana; Department of Cardiology (Prof C Andrei PhD), Department of General Surgery (I Negoi PhD, B Socea PhD), Carol Davila University of Medicine and Pharmacy, Bucharest, Romania; Digestive Diseases Research Institute (A Anoushiravani MD), School of Medicine (A Behnoush BS), Children's Medical Center (Prof F Kompani MD), Sina Trauma and Surgery Research Center (Prof V Rahimi-Movaghar MD, S Shool MD), Non-communicable Diseases Research Center (S Rahmani MD, M Rashidi MD), Department of Pathology (Prof S Tavangar MD), Tehran University of Medical Sciences, Tehran, Iran; Health Management and Economics Research Center (J Arabloo PhD), School of Medicine (M Dodangeh MD), Department of Ophthalmology (H Hasani MD), Minimally Invasive Surgery Research Center (A Kabir MD), Iran University of Medical Sciences, Tehran, Iran (M Moradi MD); University Institute of Radiological Sciences and Medical Imaging Technology (T Ashraf MS), The University of Lahore, Lahore, Pakistan; Leeds Institute of Rheumatic and Musculoskeletal Medicine (S Azadnajafabad MD), University of Leeds, Leeds, UK; Department of Nursing (N Baghcheghi PhD), Social Determinants of Health Research Center (H Koohestani PhD), Saveh University of Medical Sciences, Saveh, Iran; School of Medicine (S Bagherieh BSc, S Hassanzadeh MD), Isfahan University of Medical Sciences, Isfahan, Iran; Department of Comprehensive Nursing (B B Bantie MSc), Debre Tabor University, Debre Tabor, Ethiopia; Miami Cancer Institute (M Bardhan MD), Baptist Health South Florida, Miami, FL, USA; Department of General Surgery and Medical-Surgical Specialties (Prof G Basile MD, Prof A Biondi PhD), Department of Medical and Surgical Sciences and Advanced Technologies "GF Ingrassia" (A Maugeri PhD, Prof M Veroux PhD), University of Catania, Catania, Italy; Department of Surgery (N S Bayleyegn MD), Jimma University, Jimma, Ethiopia; Non-Communicable Diseases Research Center (NCDRC), Tehran, Iran (A Behnoush BS); Department of Medical Anatomy (A Bekele MSc), School of Public Health (N Sidamo PhD), Arba Minch University, Arba Minch, Ethiopia; Department of Anatomy (V S Bhojaraja MD), Royal College of Surgeons in Ireland Medical University of Bahrain, Busaiteen, Bahrain; Social Determinants of Health Research Center (A Bijani PhD), Babol University of Medical Sciences, Babol, Iran; Center for Biomedicine and Community Health (D Chu PhD), Vietnam National University Hanoi (VNUIS), Hanoi, Vietnam; Department of Paediatric Surgery (I S Chukwu BMedSc), Federal Medical Centre, Umuahia, Nigeria; Department of Diagnostic and Therapeutic Technologies (Prof N Cruz-Martins PhD), Cooperativa de Ensino Superior Politécnico e Universitário (Polytechnic and University Higher Education Cooperative), Vila Nova de Famalicão, Portugal; Institute for Research and Innovation in Health (i3S) (Prof N Cruz-Martins PhD), University of Porto, Porto, Portugal; USAID-JSI Digital Health Activity (B H Demessa MPH), Jimma University, Addis Ababa, Ethiopia; Sheffield Teaching Hospitals NHS Foundation Trust, Sheffield, UK (A Dhali MBBS); Faculty of Science (Prof D Diaz PhD), National Autonomous University of Mexico, Mexico City, Mexico; Department of Medicine (T C Do MD), School of Medicine (H Pham MD), Pham Ngoc Thach University of Medicine, Ho Chi Minh City, Vietnam; Health Science Center (D Dongarwar MS), University of Texas, Houston, TX, USA; Manipal Academy of Higher Education (H L Dsouza MD), Department of Pathology (S Sreeram MD), Manipal Academy of Higher Education, Manipal, India; Department of Forensic Medicine and Toxicology (H L Dsouza MD), Kasturba Medical College Mangalore, Mangalore, India; Faculty of Science and Health (M Ekholuenetale PhD), University of Portsmouth, Hampshire, UK; Department of Microbiology (T C Ekundayo PhD), University of Medical Sciences, Ondo, Ondo, Nigeria; Biomedical Informatics and Medical Statistics Department (I El Sayed PhD), Alexandria University, Alexandria, Egypt; Faculty of Medicine (M Elhadi MD), University of Tripoli, Tripoli, Libya; Houston Methodist Hospital, Houston,, TX, USA (M Elhadi MD); Department of Epidemiology and Medical Statistics (A F Fagbamigbe PhD), Faculty of Public Health (I I Olufadewa MHS), University of Ibadan, Ibadan, Nigeria; Research Centre for Healthcare and Community (A F Fagbamigbe PhD), Coventry University, Coventry, UK; director of the scientific and technological park (I R Fakhradiyev PhD), Kazakh National Medical University, Almaty, Kazakhstan; Research Center on Public Health (P Ferrara MD), University of Milan Bicocca, Monza, Italy; Department of Nursing (G Fetensa MSc), Wollega University, Nekemte, Ethiopia; Institute of Public Health (F Fischer PhD), Charité Universitätsmedizin Berlin, Berlin, Germany; Department of

Environmental Health (M Gebrehiwot DSc), Wollo University, Dessie, Ethiopia; Department of Emergency and Critical Care Medicine (M Getachew MD), Haramaya University, Harar, Ethiopia; Department of Health Systems and Policy Research (M Golechha PhD), Indian Institute of Public Health, Gandhinagar, India; Faculty of Medicine Health and Human Sciences (Prof V K Gupta PhD), Macquarie University, Sydney, NSW, Australia; Department of Surgery (J R Habib MD), University of Maryland, New York, NY, USA; Department of Clinical Pharmacology and Medicine (Prof N R Hadi PhD), University of Kufa, Najaf, Iraq; Department of Surgery (N Haep MD), Other, Berlin, Germany; Clinician Scientist Program (N Haep MD), Berlin Institute of Health, Berlin, Germany; Department of Nursing (T G Haile MSc, A N Iyasu MSc, G M Tsegay MSc), Aksum University, Aksum, Ethiopia; Department of Pharmaceutical Technology (I Hasan MPharm), University of Dhaka, Dhaka, Bangladesh; Department of Diagnostic and Interventional Radiology and Neuroradiology (J Haubold MD), Institute of Artificial Intelligence in Medicine (J Haubold MD), University Hospital Essen, Essen, Germany; Institute of Pharmaceutical Sciences (K Hayat MS), University of Veterinary and Animal Sciences, Lahore, Pakistan; Department of Pharmacy Administration and Clinical Pharmacy (K Hayat MS), Xian Jiaotong University, Xian, China; Africa Centre for Disease Control and Prevention (O S Ilesanmi PhD), University of Ibadan, Abuja, Nigeria; Department of Community Medicine (O S Ilesanmi PhD), University College Hospital, Ibadan, Ibadan, Nigeria; Division of Gastroenterology and Hepatology (S Inamdar MD), University of Arkansas for Medical Sciences, Little Rock, AR, USA; School of Health Systems and Public Health (C C D Iwu MPH), University of Pretoria, Pretoria, South Africa; Postgraduate Institute of Medicine (U Jayarajah MD), Department of Surgery (D P Wickramasinghe MD), University of Colombo, Colombo, Sri Lanka; Department of Surgery (U Jayarajah MD), National Hospital, Colombo, Sri Lanka; Department of Biochemistry (Prof S Jayaram MD), Government Medical College, Mysuru, India; Zoonoses Research Center (M Jokar DVM), Islamic Azad University, Karaj, Iran; Jahrom University of Medical Sciences, (M Jokar DVM); Department of Microbiology (N Jomehzadeh PhD), Ahvaz Jundishapur University of Medical Sciences, Ahvaz, Iran; Department of Microbiology (N Jomehzadeh PhD), Abadan School of Medical Sciences, Abadan, Iran; Department of Gastroenterology and Hepatology (A Joseph MD), Stanford University, Stanford, CA, USA; Department of Community Medicine (N Joseph MD), Manipal Academy of Higher Education, Mangalore, India; Department of Economics (C E Joshua BSc), National Open University, Benin City, Nigeria; Save Sight Institute (H Kandel PhD), University of Sydney, Sydney, NSW, Australia; Sydney Eye Hospital (H Kandel PhD), South Eastern Sydney Local Health District, Sydney, NSW, Australia; Surgery Research Unit (Prof J H Kauppila MD), University of Oulu, Oulu, Finland; Department of Molecular Medicine and Surgery (Prof J H Kauppila MD), Karolinska Institute, Stockholm, Sweden; Department of Surgery (P M Kemp Bohan MD), Brooke Army Medical Center, Seattle, WA, USA; Amity Institute of Forensic Sciences (H Khajuria PhD), Amity University, Noida, India; Department of Epidemiology (M Khan MD), Jazan University, Jazan, Saudi Arabia; Faculty of Nursing (H Khatatbeh PhD), Jerash University, Jerash, Jordan; Broad Institute of MIT and Harvard, Cambridge, MA, USA (M Kim MD); School of Health Sciences (Prof A Kisa PhD), Kristiania University College, Oslo, Norway; Department of International Health and Sustainable Development (Prof A Kisa PhD), Tulane University, New Orleans, LA, USA; College of Public Health & Health Informatics (R Kumar PhD), University of Hail, Hail, Saudi Arabia; University of Medicine and Pharmacy at Ho Chi Minh City, Ho Chi Minh City, Vietnam (T T Le MD); Department of Medical Science (M Lee PhD), Ajou University School of Medicine, Suwon, South Korea; Department of Precision Medicine (Prof S W Lee MD), Sungkyunkwan University, Suwon-si, South Korea; Department of Health Promotion and Health Education (M Li PhD), National Taiwan Normal University, Taipei, Taiwan; Department of Internal Medicine (C Lo MD), Kirk Kerkorian School of Medicine at UNLV, Las Vegas, NV, USA; Department of Emergency General and Trauma Surgery (Prof R Lunevicius DSc), Liverpool University Hospitals NHS Foundation Trust, Liverpool, UK; Department of Surgery (Prof R Lunevicius DSc), University of Liverpool, Liverpool, UK; Rama Medical College Hospital and Research Centre, Uttar Pradesh, India (K Malhotra MBBS); Institute of Applied Health Research (K Malhotra MBBS), University of Birmingham, Birmingham, UK; School of Public Health (K Malhotra MBBS), University of Adelaide, Adelaide, SA, Australia; Division of Pediatric Hospital Medicine (R P Mediratta

MD), Stanford University, Palo Alto, CA, USA; Division of Breast Surgery (T J Meretoja MD), Helsinki University Hospital, Helsinki, Finland; University of Helsinki, Helsinki, Finland (T J Meretoja MD); University Centre Varazdin (T Mestrovic PhD), University North, Varazdin, Croatia; Department of Radiology (M Mirza-Aghazadeh-Attari MD, A Zarrintan MD), Cardiovascular Research Center (M Rahimi MD), Tabriz University of Medical Sciences, Tabriz, Iran; Social Determinants of Health Center (M Mirza-Aghazadeh-Attari MD), Urmia University of Medical Sciences, Urmia, Iran (R Valizadeh PhD); Molecular Biology Unit (N S Mohamed MSc), Bio-Statistical and Molecular Biology Department (N S Mohamed MSc), Sirius Training and Research Centre, Khartoum, Sudan; Clinical Epidemiology and Public Health Research Unit (L Monasta DSc), Burlo Garofolo Institute for Maternal and Child Health, Trieste, Italy; Charles Sturt University (M Moni PhD), Charles Sturt University, Bathurst, NSW, Australia; Department of Surgery (G D Mukoro MD), Ahmadu Bello University Teaching Hospital, Zaria, Nigeria; Clinical Epidemiology Research Unit (E Murillo-Zamora PhD), Mexican Institute of Social Security, Villa de Alvarez, Mexico; Postgraduate in Medical Sciences (E Murillo-Zamora PhD), Universidad de Colima, Colima, Mexico; Laboratory of Public Health Indicators Analysis and Health Digitalization (M Naimzada MD), Moscow Institute of Physics and Technology, Dolgoprudny, Russia; Experimental Surgery and Oncology Laboratory (M Naimzada MD), Kursk State Medical University, Kursk, Russia; Department of Medical Laboratory Analysis (H H Najmuldeen PhD), Cihan University Sulaymaniya, Sulaymaniyah, Iraq; Department of Dental Public Health (Z S Natto DrPH), King Abdulaziz University, Jeddah, Saudi Arabia; Department of Health Policy and Oral Epidemiology (Z S Natto DrPH), Harvard University, Boston, MA, USA; Department of General Surgery (I Negoi PhD), Emergency University Hospital Bucharest, Bucharest, Romania; Cardiovascular Research Department (H Q Nguyen MD), Methodist Hospital, Merrillville, IL, USA; Department of General Surgery (T K Nikolouzakis PhD), University Hospital of Heraklion, Heraklion, Greece; Laboratory of Toxicology (T K Nikolouzakis PhD), University of Crete, Heraklion, Greece; Slum and Rural Health Initiative Research Academy (I I Olufadewa MHS), Slum and Rural Health Initiative, Ibadan, Nigeria; Department of Forensic Medicine and Toxicology (Prof J Padubidri MD), Kasturba Medical College, Mangalore, India; Research Department (A Pandey MPH), Nepal Health Research Council, Kathmandu, Nepal; Research Department (A Pandey MPH), Public Health Research Society Nepal, Kathmandu, Nepal; Department of Epidemiology and Community Health (R R Parikh MD), Department of Surgery (J Rickard MD), University of Minnesota, Minneapolis, MN, USA; Institute of Infection and Immunity (R C G Pollok FRCP), St George's University of London, London, UK; Department of Population Science and Human Resource Development (Prof M Rahman DrPH), University of Rajshahi, Rajshahi, Bangladesh; Department of Primary Care and Public Health (Prof S Rawaf MD), Imperial College London, London, UK; Academic Public Health England (Prof S Rawaf MD), Public Health England, London, UK; Department of Surgery (J Rickard MD), University Teaching Hospital of Kigali, Kigali, Rwanda; Department of Epidemiology (S Roy MPH), Florida International University, Miami, FL, USA; School of Population Health (Prof B A Saddik PhD), University of New South Wales, Sydney, NSW, Australia; Clinical and Biomedical Research Center (Prof U Saeed PhD), Foundation University, Islamabad, Pakistan; International Center of Medical Sciences Research (ICMSR), Islamabad, Pakistan (Prof U Saeed PhD); Faculty of Pharmacy (Prof M A Saleh PhD), Mansoura University, Mansoura, Egypt; Mark and Mary Stevens Neuroimaging and Informatics Institute (S Salehi MD), University of Southern California, Los Angeles, CA, USA; Department of Entomology (A M Samy PhD), Medical Ain Shams Research Institute (MASRI) (A M Samy PhD), Ain Shams University, Cairo, Egypt; Department of Nutrition and Preventive Medicine (Prof J Sanabria MD), Department of Pediatrics (S Sankararaman MD), Case Western Reserve University, Cleveland, OH, USA; Department of Pediatrics (S Sankararaman MD), University Hospitals Rainbow Babies & Children's Hospital, Cleveland, OH, USA; Emergency Department (S Senthilkumaran PhD), Manian Medical Centre, Erode, India; Department of Microbiology (P A Shah MBBS), Rajiv Gandhi University of Health Sciences, Bangalore, India; Other, (P A Shah MBBS); Department of Pediatrics and Child Health Nursing (M M Sibhat MSc), Dilla University, Dilla, Ethiopia; School of Medicine (Prof J A Singh MD), University of Alabama at Birmingham, Houston, TX, USA; Medicine Service (Prof J A Singh MD), US Department of Veterans Affairs (VA), Houston, TX,

USA; Department of Surgery (B Socea PhD), "Sf. Pantelimon" Emergency Clinical Hospital Bucharest, Bucharest, Romania; Department of Nursing (Y Solomon MSc), Department of Public Health (Y M Tefera MPH), Dire Dawa University, Dire Dawa, Ethiopia; Department of Medical Informatics (S Tabatabaei PhD), Clinial Research Development Unit (S Tabatabaei PhD), Mashhad University of Medical Sciences, Mashhad, Iran; Department of Surgery (K Tan PhD), National University of Singapore, Singapore, Singapore; Department of Gastroenterology (N Thomas MD), St. Luke's Hospital, Patanamthitta, India; Faculty of Public Health (J H V Ticoalu MPH), Universitas Sam Ratulangi (Sam Ratulangi University), Manado, Indonesia; Department of Nursing (D Tsegaye MSc), Debre Markos University, Debre Markos, Ethiopia; Department of Biochemistry and Molecular Biology (S Ullah PhD), Department of Pathology (S Ullah PhD), The University of Texas Medical Branch at Galveston, Galveston, TX, USA; Department of Nursing (A N Usman MSc), Addis Ababa University, Addis Ababa, Ethiopia; Department of Surgery (G Verras MD), General University Hospital of Patras, Patras, Greece; College of Medicine and Veterinary Medicine (G Verras MD), University of Edinburgh, Edinburgh, UK; Department of Gastroenterology (S Wang PhD), Shanghai Jiao Tong University Affiliated Sixth People's Hospital, Shanghai, China; Department of Microbiology and Immunology (G Yahya PhD), Zagazig University, Zagazig, Egypt; Department of Cells and Tissues (G Yahya PhD), Molecular Biology Institute of Barcelona, Barcelona, Spain; Research and Development Department (I Zare BSc), Sina Medical Biochemistry Technologies, Shiraz, Iran; School of Public Health (Prof Z Zhang PhD), Wuhan University, Wuhan, China.

Authors' Contributions

Managing the overall research enterprise

M Ashworth Dirac

Writing the first draft of the manuscript

Hannah Han and M Ashworth Dirac

Primary responsibility for applying analytical methods to produce estimates

Hannah Han

Primary responsibility for seeking, cataloguing, extracting, or cleaning data; designing or coding figures and tables

Hannah Han

Providing data or critical feedback on data sources

Yohannes Habtegiorgis Abate, Bright Opoku Ahinkorah, Ali Ahmadi, Ayman Ahmed, Salman Khalifah Al-Sabah, Hubert Amu, Jalal Arabloo, Tahira Ashraf, Sara Bagherieh, Mainak Bardhan, Guido Basile, Nebiyou Simegnew Bayleyegn, Alehegn Bekele, Vijayalakshmi S Bhojaraja, Dinh-Toi Chu, Natalia Cruz-Martins, Xiaochen Dai, Berecha Hundessa Demessa, Arkadeep Dhali, M Ashworth Dirac, Thanh Chi Do, Milad Dodangeh, Haneil Larson Dsouza, Michael Ekholuenetale, Temitope Cyrus Ekundayo, Adeniyi Francis Fagbamigbe, Ildar Ravisovich Fakhradiyev, Melaku Getachew, Mahaveer Golechha, Joseph R Habib, Najah R Hadi, Nils Haep, Teklehaimanot Gereziher Haile, Erin B Hamilton, Johannes Haubold, Simon I Hay, Olayinka Stephen Ilesanmi, Chidozie C D Iwu, Shubha Jayaram, Nabi Jomehzadeh, Abel Joseph, Charity Ehimwenma Joshua, Himal Kandel, Himanshu Khajuria, Maseer Khan, Haitham Khatatbeh, Min Seo Kim, Adnan Kisa, Thao Thi Thu Le, Seung Won Lee, Munjae Lee, Stephen S Lim, Kashish Malhotra, Andrea Maugeri, Rishi P Mediratta, Ali H Mokdad, Lorenzo Monasta, Mohammad Ali Moni, Maryam Moradi, Vincent Mougin, George Duke Mukoro, Christopher J L Murray, Zuhair S Natto, Ionut Negoi, Hien Quang Nguyen, Taxiarchis Konstantinos Nikolouzakis, Jagadish Rao Padubidri, Romil R Parikh, Hoang Tran Pham, Vafa Rahimi-Movaghar, Salman Rawaf, Jennifer Rickard, Basema Ahmad Saddik, Umar Saeed, Abdallah M Samy, Juan Sanabria, Subramanian Senthilkumaran,

Pritik A Shah, Negussie Boti Sidamo, Jasvinder A Singh, Yonatan Solomon, Ker-Kan Tan, Yibekal Manaye Tefera, Nikhil Kenny Kenny Thomas, Dejen Tsegaye, Sana Ullah, Abachebissa Nuru Usman, Georgios-Ioannis Verras, Theo Vos, Song Wang, Dakshitha Praneeth Wickramasinghe, Galal Yahya, and Iman Zare.

Developing methods or computational machinery

Aleksandr Y Aravkin, Xiaochen Dai, M Ashworth Dirac, Erin B Hamilton, Hannah Han, Simon I Hay, Ali H Mokdad, Vincent Mougin, Christopher J L Murray, Austin E Schumacher, and Theo Vos.

Providing critical feedback on methods or results

Yohannes Habtegiorgis Abate, Michael Abdelmasseh, Eman Abu-Gharbieh, Tigist Demssew Adane, Bright Opoku Ahinkorah, Aqeel Ahmad, Ali Ahmadi, Ayman Ahmed, Salman Khalifah Al-Sabah, Yaser Mohammed Al-Worafi, Hubert Amu, Catalina Liliana Andrei, Amir Anoushiravani, Jalal Arabloo, Tahira Ashraf, Sina Azadnajafabad, Nayereh Baghcheghi, Sara Bagherieh, Berihun Bantie Bantie, Mainak Bardhan, Guido Basile, Nebiyou Simegnew Bayleyegn, Amir Hossein Behnoush, Alehegn Bekele, Vijayalakshmi S Bhojaraja, Ali Bijani, Katrin Burkart, Dinh-Toi Chu, Isaac Sunday Chukwu, Natalia Cruz-Martins, Xiaochen Dai, Berecha Hundessa Demessa, Daniel Diaz, M Ashworth Dirac, Thanh Chi Do, Milad Dodangeh, Deepa Dongarwar, Haneil Larson Dsouza, Michael Ekholuenetale, Temitope Cyrus Ekundayo, Iman El Sayed, Muhammed Elhadi, Adeniyi Francis Fagbamigbe, Ildar Ravisovich Fakhradiyev, Getahun Fetensa, Florian Fischer, Mesfin Gebrehiwot, Melaku Getachew, Mahaveer Golechha, Vivek Kumar Gupta, Joseph R Habib, Najah R Hadi, Nils Haep, Teklehaimanot Gereziher Haile, Ikramul Hasan, Hamidreza Hasani, Sara Hassanzadeh, Johannes Haubold, Simon I Hay, Khezar Hayat, Olayinka Stephen Ilesanmi, Sumant Inamdar, Chidozie C D Iwu, Umesh Jayarajah, Shubha Jayaram, Mohammad Jokar, Nabi Jomehzadeh, Nitin Joseph, Abel Joseph, Charity Ehimwenma Joshua, Ali Kabir, Himal Kandel, Joonas H Kauppila, Phillip M. Kemp Bohan, Himanshu Khajuria, Maseer Khan, Haitham Khatatbeh, Min Seo Kim, Adnan Kisa, Farzad Kompani, Hamid Reza Koohestani, Thao Thi Thu Le, Seung Won Lee, Munjae Lee, Ming-Chieh Li, Stephen S Lim, Chun-Han Lo, Raimundas Lunevicius, Kashish Malhotra, Andrea Maugeri, Rishi P Mediratta, Tuomo J Meretoja, Tomislav Mestrovic, Mohammad Mirza-Aghazadeh-Attari, Nouh Saad Mohamed, Ali H Mokdad, Mohammad Ali Moni, Maryam Moradi, George Duke Mukoro, Efren Murillo-Zamora, Christopher J L Murray, Hastyar Hama Rashid Najmuldeen, Zuhair S Natto, Ionut Negoi, Hien Quang Nguyen, Taxiarchis Konstantinos Nikolouzakis, Isaac Iyinoluwa Olufadewa, Jagadish Rao Padubidri, Ashok Pandey, Romil R Parikh, Hoang Tran Pham, Richard Charles G Pollok, Mehran Rahimi, Vafa Rahimi-Movaghar, Mosiur Rahman, Mohammad-Mahdi Rashidi, Salman Rawaf, Jennifer Rickard, Hamidreza Rouientan, Simanta Roy, Basema Ahmad Saddik, Umar Saeed, Mohamed A. Saleh, Sana Salehi, Abdallah M Samy, Juan Sanabria, Senthilkumar Sankararaman, Subramanian Senthilkumaran, Pritik A Shah, Sina Shool, Migbar Mekonnen Sibhat, Negussie Boti Sidamo, Jasvinder A Singh, Bogdan Socea, Yonatan Solomon, Saraswathy Sreeram, Seyyed Mohammad Tabatabaei, Ker-Kan Tan, Seyed Mohammad Tavangar, Yibekal Manaye Tefera, Nikhil Kenny Kenny Thomas, Jansje Henny Vera Ticoalu, Guesh Mebrahtom Tsegay, Dejen Tsegaye, Sana Ullah, Abachebissa Nuru Usman, Rohollah Valizadeh, Massimiliano Veroux, Georgios-Ioannis Verras, Theo Vos, Mei Wang, Song Wang, Dakshitha Praneeth Wickramasinghe, Galal Yahya, Armin Zarrintan, and Zhi-Jiang Zhang.

Drafting the work or revising it critically for important intellectual content

Yohannes Habtegiorgis Abate, Michael Abdelmasseh, Eman Abu-Gharbieh, Bright Opoku Ahinkorah, Ali Ahmadi, Ayman Ahmed, Fadwa Alhalaiqa Naji Alhalaiqa, Yaser Mohammed Al-Worafi, Hubert Amu, Catalina Liliana Andrei, Amir Anoushiravani, Jalal Arabloo, Sina Azadnajafabad, Sara Bagherieh, Mainak Bardhan, Guido Basile, Amir Hossein Behnoush, Alehegn Bekele, Vijayalakshmi S Bhojaraja, Antonio Biondi, Dinh-Toi Chu, Natalia Cruz-Martins, Arkadeep Dhali, Daniel Diaz, M Ashworth Dirac, Thanh Chi Do, Milad Dodangeh, Deepa Dongarwar, Haneil Larson Dsouza, Michael Ekholuenetale, Iman El Sayed, Muhammed Elhadi, Adeniyi Francis Fagbamigbe, Pietro Ferrara, Getahun Fetensa, Florian Fischer, Melaku Getachew, Vivek Kumar Gupta, Joseph R Habib, Najah R Hadi, Nils Haep,

Teklehaimanot Gereziher Haile, Hannah Han, Hamidreza Hasani, Sara Hassanzadeh, Johannes Haubold, Simon I Hay, Khezar Hayat, Olayinka Stephen Ilesanmi, Sumant Inamdar, Chidozie C D Iwu, Assefa N Iyasu, Umesh Jayarajah, Shubha Jayaram, Nabi Jomehzadeh, Nitin Joseph, Abel Joseph, Charity Ehimwenma Joshua, Ali Kabir, Himal Kandel, Joonas H Kauppila, Phillip M. Kemp Bohan, Himanshu Khajuria, Maseer Khan, Haitham Khatatbeh, Min Seo Kim, Adnan Kisa, Farzad Kompani, Rakesh Kumar, Thao Thi Thu Le, Chun-Han Lo, Raimundas Lunevicius, Kashish Malhotra, Andrea Maugeri, Rishi P Mediratta, Tuomo J Meretoja, Tomislav Mestrovic, Mohammad Mirza-Aghazadeh-Attari, Nouh Saad Mohamed, Ali H Mokdad, Lorenzo Monasta, Mohammad Ali Moni, Maryam Moradi, Efren Murillo-Zamora, Christopher J L Murray, Mukhammad David Naimzada, Zuhair S Natto, Ionut Negoi, Hien Quang Nguyen, Taxiarchis Konstantinos Nikolouzakis, Jagadish Rao Padubidri, Romil R Parikh, Hoang Tran Pham, Richard Charles G Pollok, Mehran Rahimi, Vafa Rahimi-Movaghar, Shayan Rahmani, Salman Rawaf, Jennifer Rickard, Hamidreza Rouientan, Basema Ahmad Saddik, Umar Saeed, Abdallah M Samy, Juan Sanabria, Pritik A Shah, Sina Shool, Negussie Boti Sidamo, Jasvinder A Singh, Bogdan Socea, Yonatan Solomon, Saraswathy Sreeram, Ker-Kan Tan, Seyed Mohammad Tavangar, Yibekal Manaye Tefera, Guesh Mebrahtom Tsegay, Dejen Tsegaye, Massimiliano Veroux, Georgios-Ioannis Verras, Theo Vos, Mei Wang, Dakshitha Praneeth Wickramasinghe, Galal Yahya, Iman Zare, and Armin Zarrintan.

Managing the estimation or publications process
M Ashworth Dirac, Erin B Hamilton, Hannah Han, Simon I Hay, Ali H Mokdad, and Christopher J L Murray.
